# Non-indicated vitamin B_12_- and D-testing among Dutch hospital clinicians: a cross-sectional analysis in data registries

**DOI:** 10.1136/bmjopen-2023-075241

**Published:** 2024-02-28

**Authors:** Joris LJM Müskens, Rudolf Bertijn Kool, Gert P Westert, Maarten Zaal, Hein Muller, Femke Atsma, S A van Dulmen

**Affiliations:** 1 IQ Health science department, Radboud University Medical Centre, Nijmegen, The Netherlands; 2 Dutch Hospital Data, Utrecht, The Netherlands

**Keywords:** Quality in health care, Hospitals, Public, Clinical Decision-Making, Retrospective Studies

## Abstract

**Objectives:**

To assess the extent of non-indicated vitamin B_12_- and D-testing among Dutch clinicians and its variation among hospitals.

**Design:**

Cross-sectional study using registration data from 2015 to 2019.

**Participants:**

Patients aged between 18 and 70 years who received a vitamin B_12_- or D-test.

**Primary and secondary outcome measures:**

The proportion of non-indicated vitamin B_12_- and D-testing among Dutch clinicians and its variation between hospitals (n=68) over 2015–2019.

**Results:**

Between 2015 and 2019, at least 79.0% of all vitamin B_12_-tests and 82.0% of vitamin D-tests lacked a clear indication. The number of vitamin B_12_-tests increased by 2.0% over the examined period, while the number of D-tests increased by 12.2%. The proportion of the unexplained variation in non-indicated vitamin B_12_- and D-tests that can be ascribed to differences between hospitals remained low. Intraclass correlation coefficients ranged between 0.072 and 0.085 and 0.081 and 0.096 for non-indicated vitamin B_12_- and D-tests, respectively. The included casemix variables patient age, gender, socioeconomic status and hospital size only accounted for a small part of the unexplained variation in non-indicated testing. Additionally, a significant correlation was observed in non-indicated vitamin B_12_- and D-testing among the included hospitals.

**Conclusion:**

Hospital clinicians order vitamin B_12_- and D-tests without a clear indication on a large scale. Only a small proportion of the unexplained variation could be attributed to differences between hospitals.

**STRENGTHS AND LIMITATIONS OF THIS STUDY**

- This study has examined the ordering and appropriateness of vitamin B_12_- and D-testing among hospital clinicians and its variation using data over several consecutive years.
- The use of a detailed nationally representative database allowed us to generate an accurate and reliable overview of the extent of (non-indicated) vitamin B_12_- and D-testing among exclusively hospital clinicians within the Dutch healthcare system.
- Additionally, the use of registration data derived directly from hospital registration systems enabled us to accurately distinguish appropriate from inappropriate vitamin testing using diagnosis codes rather than the proxy of testing interval.

## Introduction

Low-value diagnostic testing is a serious problem in most healthcare systems.^1^ Low-value care is defined as care that offers no net benefit for the patient and which can be associated with harmful outcomes and wasteful spending.^2–4^ Within diagnostic testing, studies show that both vitamin B_12_- and D-tests are frequently ordered within medical practice.^5–8^ However, there are only a few indications that justify the ordering of a vitamin B_12_- or D-test which are described in international guidelines. Among healthy adults, almost no indications for vitamin B_12_- and D-testing exist.^9 10^ International guidelines for hospital clinicians clearly state that vitamin B_12_- and D-tests should only be ordered in specific scenarios, such as in patients with Coeliac or Crohn’s disease, and not in patients with only vague complaints.^9 11–14^ Assessments indicate that as much as 77% of vitamin B_12_-tests and 91% of vitamin D-tests could lack an indication.^7 15–23^

Studies show that between 8% and 28% of vitamin B_12_-tests and between 6% and 91% of vitamin D-tests could be non-indicated. Most of these studies have been conducted among general practitioners (GPs).^17 19 21 23^ Only a few studies have clearly specified that their assessment of the volume of non-indicated vitamin testing concerned hospital clinicians alone. Two studies from Italy reported that on average 17% and 6% of vitamin B_12_- and D-tests were potentially non-indicated.^7 18^ However, these assessments used data from a single hospital and therefore do not provide a representative overview. Furthermore, these studies based their assessment of appropriateness on a proxy such as testing interval and not directly by examining the diagnosis for which the test was requested.

In order to address non-indicated vitamin B_12_- and D-testing, an accurate assessment of the magnitude and variation of non-indicated testing is needed. We therefore aimed to assess both the volume and proportions of patients aged between 18 and 70 years who potentially received non-indicated vitamin B_12_- and D-tests among Dutch hospital clinicians between 2015 and 2019 using clinical registration data. We also examined the hospital variation in non-indicated vitamin B_12_- and D-testing in order to identify opportunities for improvement. The proportion of outpatient visits receiving a non-indicated vitamin B_12_- or D-test among the hospitals included, and the diagnosis codes often associated with vitamin B_12_- and D-testing over 2019 were also explored. In doing so, we aimed to gain more insight into the volume of non-indicated vitamin testing among hospital clinicians and the diagnoses underlying such testing.

## Methods

### Design and database

We conducted a cross-sectional study using registration data among Dutch hospital clinicians between 2015 and 2019. Data were obtained from the Dutch National Basic Hospital Care Registration (*Landelijke Basisregistratie Ziekenhuiszorg, LBZ*).^24^ The LBZ contains medical, financial and administrative information from all patients undergoing treatment in any Dutch hospital. All vitamin B_12_- and D-tests ordered by clinicians in Dutch hospitals over the examined time period were extracted; including the associated diagnosis codes, patient age, gender, socioeconomic status (SES) and hospital size. After consulting paediatricians and geriatricians, we limited our analysis to patients aged 18–70 years. Both paediatricians and geriatricians indicated that for patients below 18 and above 70 years, there are many regional screening protocols which also often vary between hospitals. Patients were assigned to one of three age categories; 18–29, 30–49 and 50–70 years. SES scores were derived from a table containing SES scores on the level of four-digit postal codes as published by the Dutch Institute for Social Research in 2017.^25^ Patients were also assigned to one of six SES categories, based on quintiles calculated with the SES information from all Dutch neighbourhoods. Hospital size was operationalised by assigning a hospital to one of three categories (small/medium/large) based on tertiles calculated using the number of outpatient visits encountered over 2019.

### Patient and public involvement

No patients or members of the public were directly involved in the study. Owing to the nature of this study and data privacy constraints, no patients or members of the public were involved in the study design, analysis, interpretation of data or revision of the manuscript.

### Analysis of trends in proportions of non-indicated vitamin B_12_- and D-testing among hospital clinicians

For the assessment of the proportion of justified indication versus non-indicated vitamin B_12_- and D-testing, we used a service lens, as previously described by Chalmers *et al*.^26^ This entails that all vitamin B_12_- or D-tests ordered were included in our denominator and all vitamin B_12_- and D-tests ordered with no indication in our numerator. For our distinction of non-indicated vitamin testing, we followed several steps. First, all recommendations regarding vitamin B_12_- or D-testing were extracted from the relevant guidelines. Initially, we reviewed the Dutch guidelines of hospital clinicians published by the Federation of Medical Specialists for indications for vitamin B_12_- and D-testing.^10^ We managed to identify little to no recommendations concerning indications regarding the use of vitamin B_12_- or D-tests. We therefore chose to supplement these with indications derived from Dutch GP guidelines.^13 14^ Second, the ICD10 codes corresponding to the diagnoses which warrant the ordering of a vitamin B_12_- or D-test were collected from these recommendations. Third, the resulting list of ICD10 codes was reviewed by the involved experts to prevent missing relevant codes or diagnoses. We consulted two expert clinicians (an internal medicine physician and a haematologist) in the process of generating the list of indications justifying a vitamin B_12_- or D-test. Fourth, after completing the list of ICD10 codes, all Clinical Classification Software (CCS) codes associated with these ICD10 codes were extracted from the LBZ database. Subsequently, all Diagnosis-Treatment Combination (DTC) codes associated with the list of relevant CCS codes were extracted. The resulting list was, again, checked for completeness before starting with the assessment of indicated vitamin tests. This process was repeated until all researchers and clinicians agreed on the accuracy of the list of indications justifying vitamin B_12_- and D-testing. Online supplemental file 1 lists ICD10 and DTC codes used to determine the appropriateness of the identified vitamin B_12_- or D-tests. It also contains a description of how we identified and linked the identified ICD10 codes to each of the patients included. In an effort to provide potential handles for the design of interventions, we also examined which diagnosis codes were frequently associated with non-indicated tests.

10.1136/bmjopen-2023-075241.supp1 Supplementary data

#### Assessment of hospital variation in non-indicated vitamin B_12_- and D-testing

Hospital variation in non-indicated vitamin B_12_- and D- testing was assessed using a multilevel logistic regression analysis, with a random effect for hospital. Separate models per year were made to assess whether the variation in non-indicated vitamin B_12_- and D-testing was robust over time. Generalised variance inflation factors were calculated to test for collinearity among the included variables before multilevel analysis was conducted (online supplemental file 2). Models were adjusted for the casemix variables: patient age, gender, SES and hospital size. We corrected for patient age, gender and SES, as previous research showed that these affect the amount of care that patients require, receive and have access to.^27–31^ We included a proxy for hospital size (eg, the total number of outpatient visits in each year) while recent evidence shows that larger healthcare providers tend to provide more low-value care.^32^ Vitamin tests conducted in patients with a missing SES score or DTC code were excluded from the analysis. Intraclass correlation coefficients (ICCs) were calculated to assess which part of the unexplained variation in non-indicated vitamin B_12_- and D-testing could be ascribed to differences between the included hospitals, using the method of Snijders and Bosker to assess the error variance.^33^

10.1136/bmjopen-2023-075241.supp2 Supplementary data

#### Correlation in non-indicated vitamin testing over 2019

Additionally, we also examined whether a correlation existed between the proportions of outpatient visits that received a non-indicated vitamin B_12_- or D-test over 2019 among the hospitals included. Correlations were assessed using the Pearson correlation coefficient for normally distributed variables and the Spearman correlation coefficient for non-normally distributed variables. Normality was assessed using both density plots and the Shapiro-Wilk test.

## Results

### Volume vitamin tests among Dutch hospital clinicians

Table 1 provides a general overview of the population characteristics of the population included in our study. Between 2015 and 2019, the number of vitamin B_12_- and D-tests ordered by clinicians increased by 2.0% (from 275 032 to 280 522) and 12.2% (from 300 013 to 336 736), respectively. A similar trend was also observed in the proportion of patients who received at least one vitamin B_12_- or D-test, increasing by 2.5% and 11.3% over the examined period. The amount of vitamin B_12_- and D-tests ordered among women remained almost twice as high compared with men over the entire period examined. Table 2 provides an overview of the outcomes, and online supplemental file 3 contains a more detailed breakdown by gender, age and SES groups on the patient level. The number of patients with at least one vitamin test increases rapidly with age. The included patients, with at least one vitamin B_12_- or D-test, showed to be more equally distributed over the SES categories. Only in the highest SES category, a slight decrease in the number of patients with a vitamin determination was observed.

10.1136/bmjopen-2023-075241.supp3 Supplementary data

**Table 1:** Overview of the study population characteristics of the used population over the entire period examined (2015–2019)

General info regarding the used population between 2015 and 2019	Number or proportion	Min	Max	Median	IQR
Total number of unique patients	9 214 425	N.A.	N.A.	N.A.	N.A.
Gender (% female)	64.50%	N.A.	N.A.	N.A.	N.A.
Average number of patients among the hospitals (±SD)	126 722 (±169 110)	37	1 244 526	126 722	119 601.8
Average number of unique patients among the hospitals (±SD)	47 561 (±54 317.5)	32	415 673	36 561	38 590
Average age of the patients included (±SD)	47.91 (±14.60)	18	70	N.A.	N.A.
Average SES category of the patients included (±SD)	2.86 (±1.43)	1	6	N.A.	N.A.
Average no. of outpatient visits among the hospitals included	1 721 337 (±819 148)	470 117	4 659 172	N.A.	N.A.

SES, socioeconomic status.

**Table 2:** Overview of vitamin B_12_- and D-tests performed among hospital clinicians over the period examined (2015–2019)

Year	2015	2016	2017	2018	2019
No. of hospitals included	63	64	66	69	68
Total no. vitamin B_12_-tests	275 032	265 546	279 524	279 957	280 522
Total no. vitamin B_12_-tests in hospital with a registered DTC code	118 126	163 244	188 063	208 012	213 308
Vitamin B_12_-test with a registered DTC without clear indication (%)	79.0	78.0	78.2	78.3	78.1
Total no. of patients with (at least) one vitamin B_12_-test	233 541	226 999	239 033	240 063	239 351
Total no. of patients with (at least) one vitamin B_12_-test with a registered DTC code	103 540	141 507	162 900	178 933	183 204
Total no. of patients with (at least) one non-indicated vitamin B_12_-test (with a registered DTC code)	83 549	111 986	128 872	141 914	145 039
Patients who received at least one vitamin B_12_-test that was considered non-indicated	80.7	79.1	79.1	79.3	79.2
No. of hospitals included	62	61	63	65	65
Total no. vitamin D-tests	300 013	300 427	322 703	323 074	336 736
Total no. vitamin D-tests in hospital with a registered DTC code	121 892	179 271	208 361	230 620	242 040
Vitamin D-test with a registered DTC without clear indication (%)	82.5	82.9	82.7	82.5	82.0
Total no. of patients with (at least) one vitamin D-test	244 834	247 186	264 811	265 362	272 380
Total no. of patients with (at least) one vitamin D-test with a registered DTC code	101 932	148 660	172 342	189 423	196 452
Total no. of patients with (at least) one non-indicated vitamin D-test (with a registered DTC code)	84 414	124 162	143 408	157 431	162 345
Patients who received at least one vitamin B_12_-test that was considered non-indicated (%)	82.8	83.5	83.2	83.1	82.6

DTC, Diagnosis Treatment Combination.

### Non-indicated testing

Between 2015 and 2019, around 78% of the vitamin B_12_-tests conducted among patients aged between 18 and 70 years, with a registered DTC code, lacked an indication. In the case of vitamin D-testing, around 82% of determinations had no clear indication. Although the number of vitamin tests is higher among women, no large differences in proportions of non-indicated testing were observed between genders. In the case of both age and SES, the proportion of patients with a non-indicated vitamin B_12_- or D-test remains relatively constant across all groups over the study period (online supplemental file 3). With the proportion of non-indicated testing remaining around 80.0% for vitamin B_12_ and 83.5% for vitamin D across the different age and SES. Our analysis of diagnosis codes that are most often associated with non-indicated vitamin B_12_- and D-testing revealed that tests are ordered for various reasons. Similar diagnostic codes were associated with both non-indicated vitamin B_12_- and D-tests, including general malaise, fatigue without diagnosis and ulcerative colitis. Online supplemental file 4 contains an overview of the top 20 diagnosis codes for both vitamin B_12_- and D-tests.

10.1136/bmjopen-2023-075241.supp4 Supplementary data

### Hospital variation in non-indicated vitamin B_12_- and D-tests among Dutch hospital clinicians

**Figure 1:**
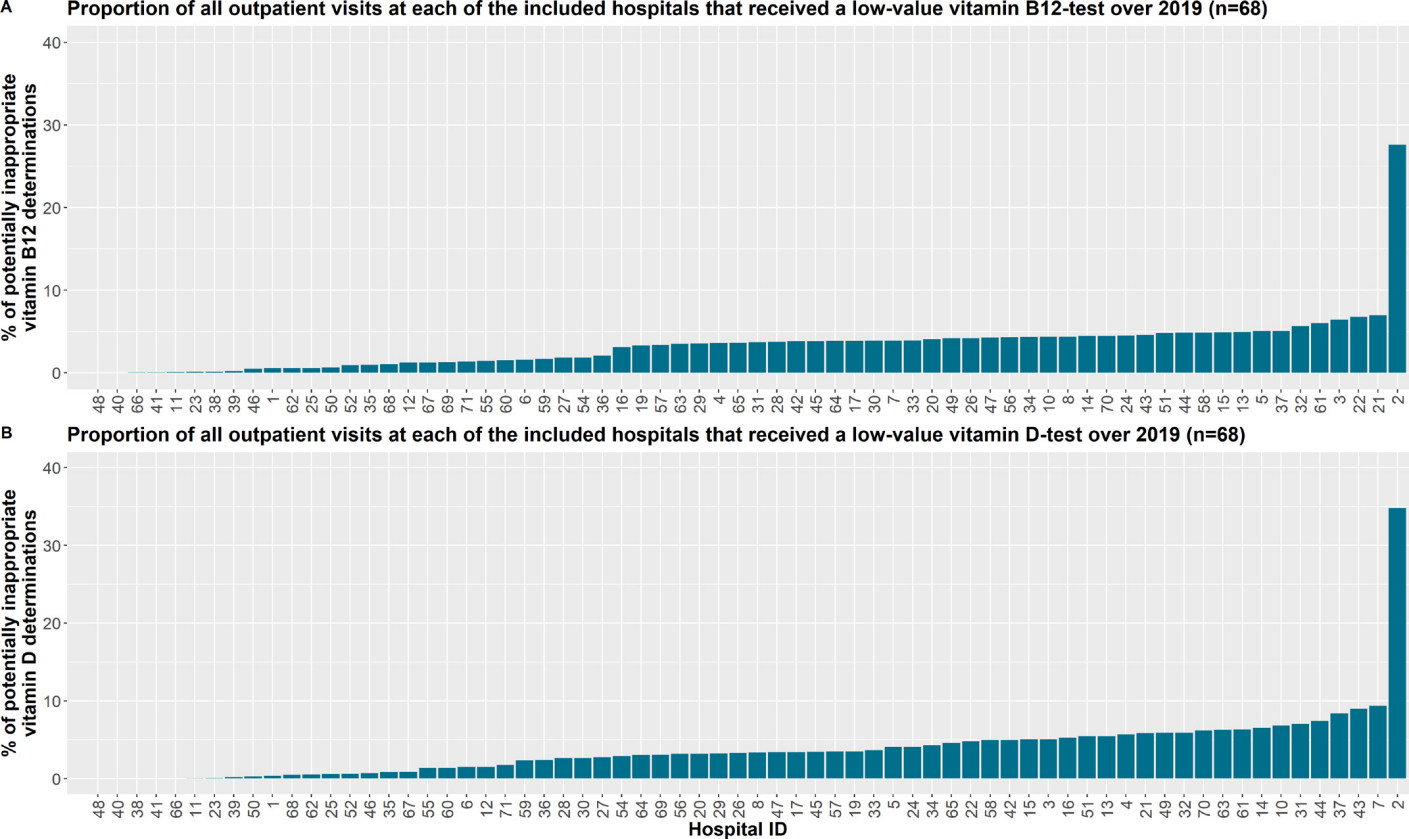
Proportion of all outpatient visits at each of the included hospitals that received a low-value vitamin (A) B_12_- or (B) D-test over 2019 (n=68).

The ICCs of the models uncorrected for casemix remained around 9% (ranging from 8.3% to 9.5%) and 9.5% (ranging from 8.5% to 10.1%) for the vitamin B_12_- and D-models over time. The ICCs of the casemix-corrected vitamin B_12_- and D-models remained stable around 8.0% (ranging between 7.2% and 8.5%) and 9% (ranging between 8.1% and 9.6%), respectively, throughout the examined period. Online supplemental file 5 contains the ICCs of all models. Casemix correction minimally impacted the calculated ICCs in the case of both the vitamin B_12_- and D-models. The proportion of outpatient visits receiving a non-indicated vitamin B_12_- or D-test over 2019 varied widely among the hospitals included, ranging from 0% to 27.6% for vitamin B_12_ and 0.02% to 34.8% for vitamin D (see figure 1 and online supplemental file 6).

10.1136/bmjopen-2023-075241.supp5 Supplementary data

10.1136/bmjopen-2023-075241.supp6 Supplementary data

### Correlation in proportions of non-indicated vitamin B_12_- and D-testing

Normality testing (and inspection of density plots) revealed that both the proportions of vitamin B_12_- and D-testing observed among the hospitals are non-normally distributed. The subsequent correlation analysis revealed the presence of a significant positive correlation (r=0.86, p<0.001) between the proportions of non-indicated vitamin B_12_- and D-testing among the included hospitals. Online supplemental file 7 contains the density plots, normality test results and correlation analysis outcomes for both non-indicated vitamin B_12_- and D-tests.

10.1136/bmjopen-2023-075241.supp7 Supplementary data

## Discussion

Between 2015 and 2019, around 78.0% and 82.0% of vitamin B_12_- and D-tests ordered by Dutch hospital clinicians in patients aged 18–70 lacked a clear indication. The total number of vitamin B_12_-tests ordered increased by 2.0%, while the total number of vitamin D-tests increased by 12.2%. Although the total number of vitamin determinations increased, the proportion of patients with at least one vitamin test remained relatively constant (around 80% for vitamin B_12_ and 83% for vitamin D). Women received approximately twice as many vitamin B_12_- and D-tests, as well as non-indicated tests, over the examined period compared with men. Our analysis of hospital variation in non-indicated vitamin B_12_- and D-testing revealed a moderate hospital variation. Furthermore, only a relatively small part of the unexplained variation in non-indicated vitamin B_12_- and D- testing could be ascribed to differences between the hospitals included, suggesting that the problem of non-indicated vitamin testing is present among all hospitals. Correlation analysis over 2019 revealed a fairly strong positive (r=0.86) correlation between the rates of non-indicated vitamin B_12_- and D-testing among the hospitals included.

### Comparison to existing literature

The proportion of non-indicated vitamin B_12_- and D-testing among clinicians found in our study (78.0% and 82.0%, respectively) is substantially larger than the proportions reported by other studies. For example, two studies from Italy conducted among hospital clinicians report that on average 17.0% and 6.0% of vitamin B_12_- and D-tests could potentially be non-indicated,^7 18^ which is substantially lower than the proportions observed in our study. Our findings are more in line with assessments that are not limited to hospital clinicians. Hence, studies conducted among GPs indicate that between 8.0% and 28.0% of vitamin B_12_-tests^7 15 16 18^ and between 7.0% and 91.0% of vitamin D-tests could be considered non-indicated.^7 15 19 21 23^ Among these studies, only Naugler *et al* and Gonzalez-Chica reported proportions of non-indicated vitamin D testing which is similar to ours. Naugler *et al*, found that following an intervention the prevalence of non-indicated vitamin D-testing decreased by 91.4%.^19^ While Gonzalez-Chica reported 76.5% of vitamin D-tests to be non-indicated after the introduction of new Medicare criteria for rebates. Besides population differences (eg, GPs vs hospital clinicians), varying definitions of non-indicated testing and methods used could also account for the large differences in assessment outcomes. For example, both our study and that of Naugler *et al* used diagnosis codes to distinguish the appropriateness of vitamin testing.^19^ Most assessments of the appropriateness of vitamin testing performed to date used testing intervals or laboratory results to discern non-indicated testing, as did Gonzalez-Chica.^23^ This methodological difference might explain the large differences in assessment outcomes. Furthermore, the studies that did not limit their assessment to vitamin testing among GPs were often conducted within a single hospital.^7 18^ Moreover, most studies that assessed vitamin B_12_- or D-testing do not specify which type of physicians (GP/hospital clinicians) or vitamin B_12_- and D-tests were included, making comparison to our study challenging.

The absence of clear recommendations regarding the ‘appropriate’ use of vitamin testing in clinician guidelines does not aid clinicians to appropriately order vitamin testing. The current guidelines for hospital clinicians offer little direction on the appropriate use of vitamin B_12_- or D-tests, leaving clinicians with little guidance when deciding whether to order such tests. These difficulties are magnified by the large knowledge gaps regarding the exact roles of vitamin B_12_ and D within the human body and its metabolism.^34–37^ The combination of these factors may provide an explanation for the high proportion of non-indicated testing. Hence, due to the lack of a clear understanding of the roles of vitamin B_12_ and D within the body and the fear of missing a diagnosis, clinicians may engage in defensive behaviour resulting in the ordering of vitamin tests.

### Strengths and limitations

A strength of this study is its novelty in examining the ordering and appropriateness of vitamin B_12_- and D-testing among hospital clinicians and its variation over several consecutive years. A second strength is that we used a detailed nationally representative database. This allowed us to generate an accurate and reliable overview of the extent of vitamin B_12_- and D-testing among solely hospital clinicians. Furthermore, it also enabled us to accurately distinguish appropriate from inappropriate vitamin testing using diagnosis codes rather than the proxy of testing interval.

However, our study is also prone to limitations. First, we based our distinction of appropriateness mainly on expert opinion and indications derived from the Dutch GP guidelines, as no universal guideline regarding vitamin testing among hospital clinicians exists. We therefore might have misclassified some of the tests from clinicians as being non-indicated. However, we tried to minimise the risk of misclassification by closely collaborating with the involved experts with respect to the creation of the list of indications. Additionally, some differences exist between international and Dutch guidelines regarding indications for vitamin B_12_- and D-testing. For example, guidelines from the USA indicate that vitamin B_12_-testing is considered indicated in patients with cognitive impairments or dementia.^38^ However, these are not considered as an indication according to the nationwide guidelines published by the Dutch federation of medical specialists.^10^ Potentially, such recommendations could exist in local protocols of Dutch hospitals, which unfortunately are not publicly available. This makes it difficult to compare international assessment outcomes to our study, while subtle differences between guidelines might cause large differences in the used criteria of appropriateness (and subsequently the reported outcomes).

Second, the use of DTC codes enabled us to accurately distinguish the appropriateness of vitamin tests but also has a drawback. DTC diagnosis codes are generally less specific compared with the ICD10 codes described in the used guidelines. We therefore might have misclassified some vitamin tests as being appropriate. Furthermore, although DTC diagnosis codes provide a lot of insight into the diagnosis associated with practices, their registration is prone to misregistration.^39^ Clinicians have a vast amount of (often similar) DTC codes to choose from when registering a diagnosis code, thereby adding another layer of complexity to the correct registration of diagnoses. Furthermore, DTC codes are updated as the patient passes through the healthcare system. The registered DTC code therefore does not necessarily represent the initial reason (or diagnosis) for which the vitamin determination was ordered but rather reflects the final diagnosis.

Third, some Dutch hospitals outsource their tests to external commercial laboratories, which are not registered in the LBZ. Our estimate of vitamin testing by clinicians therefore is not complete. However, according to the registration of the National Statistical Office, Statistics Netherlands, 82 hospitals were active in 2019. Since we were able to include data from the majority of the hospitals in the Netherlands in our study (68/82, eg, 83.0% of all hospitals), we do not expect to have missed much in our analysis.^40^

### Implications for research and practice

Our assessment reveals that a large proportion of vitamin B_12_- and D- tests are ordered without a clear indication justifying their use. The total volumes of vitamin B_12_- and D-tests have increased over the years and show no inclination of declining. Based on publicly available fares, we estimate that roughly €3.8 million has been spent on non-indicated vitamin B_12_- and D-tests in 2019 by Dutch hospital clinicians alone.^41^ This estimate of the potential savings, however, is very rough, while it only accounts for the cost price of a vitamin B_12_- or D-determination. The observed incidence rates of non-indicated vitamin B_12_- and D-testing, however, suggest that there is ample opportunity to reduce vitamin testing among Dutch clinicians. Especially since non-indicated vitamin B_12_- and D-tests are often ordered for similar diagnoses, and a positive correlation exists between the proportions of non-indicated testing within the same hospitals. We know that there are effective interventions to reduce inappropriate vitamin testing among GPs. A study among Dutch GPs showed that providing both education and feedback successfully reduced the amount of vitamin tests ordered by 20%–25%.^42^ Similar interventions might therefore also be effective among hospital clinicians to reduce (non-indicated) vitamin testing. Alternatively, more emphasis could be placed on the institution of fortification and supplementation guidelines to achieve adequate vitamin B_12_ and D intake among the Dutch population. Especially since, the implementation of such guidelines have shown to positively affect vitamin status among the population rendering vitamin testing obsolete in most cases.^43–45^ Future research could focus on further examination of patient and physicians’ characteristics associated with non-indicated vitamin B_12_- and D-testing. Unfortunately, information regarding the requesting physician (age, sex, etc) of non-indicated tests was not available to us in our study. Insight into physician characteristics associated with non-indicated testing could aid in the design of interventions aiming to address the problem of non-indicated vitamin testing.

### Conclusion

Our research shows that the number of vitamin B_12_-tests slightly increased over the examined time period, while the number of vitamin D tests substantially increased among hospital clinicians. Throughout the examined period, the proportion of B_12_- and D-tests without clear indication remained high and is substantially higher compared with similar (international) assessments. The observed difference in assessment outcome can potentially be explained by differences in methods and definitions used to identify and define non-indicated vitamin B_12_- and D-tests (eg, the use of associated diagnosis codes instead of test results or proxies such as testing interval). We also observed the presence of moderate hospital variation, but this variation could not be explained by the included patient and hospital characteristics age, sex, SES and hospital size. Hospitals hardly differ in the task they have to undertake: bring down the number of non-indicated B_12_- and D-tests.

## Supplementary Material

Reviewer comments

Author's manuscript

## Data Availability

Data are available upon reasonable request. The source data are not freely available. However, the data used to conduct our analysis are available upon reasonable request after consultation with the data supplier.

## References

[1] Müskens JLJM , Kool RB , van Dulmen SA , et al . Overuse of diagnostic testing in Healthcare: a systematic review. BMJ Qual Saf 2022;31:54–63. 10.1136/bmjqs-2020-012576 PMC8685650 33972387

[2] Brownlee S , Chalkidou K , Doust J , et al . Evidence for overuse of medical services around the world. Lancet 2017;390:156–68. 10.1016/S0140-6736(16)32585-5 28077234 PMC5708862

[3] Colla CH , Mainor AJ , Hargreaves C , et al . Interventions aimed at reducing use of low-value health services: A systematic review. Med Care Res Rev 2017;74:507–50. 10.1177/1077558716656970 27402662

[4] Scott IA , Duckett SJ . In search of professional consensus in defining and reducing low-value care. Med J Aust 2015;203:179–81. 10.5694/mja14.01664 26268286

[5] Essig S , Merlo C , Reich O , et al . Potentially inappropriate testing for vitamin D deficiency: a cross-sectional study in Switzerland. BMC Health Serv Res 2020;20:1097. 10.1186/s12913-020-05956-2 33246469 PMC7694269

[6] Crowe FL , Jolly K , MacArthur C , et al . Trends in the incidence of testing for vitamin D deficiency in primary care in the UK. BMJ Open 2019;9:e028355. 10.1136/bmjopen-2018-028355 PMC6561453 31167871

[7] Lanzoni M , Fornili M , Felicetta I , et al . Three-year analysis of repeated laboratory tests for the markers total cholesterol, Ferritin, vitamin D, vitamin B(12), and folate, in a large research and teaching hospital in Italy. J Eval Clin Pract 2017;23:654–61. 10.1111/jep.12696 28078809

[8] O’Sullivan JW , Stevens S , Hobbs FDR , et al . Temporal trends in use of tests in UK primary care, 2000-15: retrospective analysis of 250 million tests. BMJ 2018;363:k4666. 10.1136/bmj.k4666 30487169 PMC6260131

[9] American Society for Clinical Pathology . Thirty five things physicians and patients should question. 2021. Available: https://www.choosingwisely.org/clinician-lists/ascp-do-not-order-red-blood-cell-folate-levels/

[10] Richtlijnen database . Federatie Medisch Specialisten. 2022. Available: https://richtlijnendatabase.nl/

[11] Dutch College of Clinical Chemistry and Laboratory Medicine & College of medical immunologists . Verstandige Keuzes Bij Het Aanvragen En Interpreteren van Laboratoriumdiagnostiek 2015.

[12] Canadian Association of Pathologists . Choosingwisely Canada Pathology Recommendations. 2021.

[13] Nederlands Huisartsen Genootschap . Laboratoriumdiagnostiek Vitamine B12-Deficiëntie (LESA): NHG. 2018. Available: https://www.nhg.org/themas/publicaties/laboratoriumdiagnostiek-vitamine-b12-volledige-tekst

[14] Nederlands Huisartsen Genootschap . Vitamine D-Deficiëntie (LESA Laboratoriumdiagnostiek): NHG. 2018. Available: https://www.nhg.org/themas/publicaties/vitamine-d-deficientie-samenvatting?tmp-no-mobile=1]

[15] Chami N , Simons JE , Sweetman A , et al . Rates of inappropriate laboratory test utilization in Ontario. Clin Biochem 2017;50:822–7. 10.1016/j.clinbiochem.2017.05.004 28483406

[16] Morgen EK , Naugler C . Inappropriate repeats of six common tests in a Canadian city: a population cohort study within a laboratory Informatics framework. Am J Clin Pathol 2015;144:704–12. 10.1309/AJCPYXDAUS2F8XJY 26486733

[17] Gill P , Guo M , Lau CK , et al . Vitamin B12 test volume data before and after the implementation of an educational province-wide intervention to reduce redundant testing in Alberta. Data Brief 2019;27:104785. 10.1016/j.dib.2019.104785 31788514 PMC6880122

[18] Tamburrano A , Vallone D , Carrozza C , et al . Evaluation and cost estimation of laboratory test Overuse in 43 commonly ordered parameters through a computerized clinical decision support system (CCDSS) in a large University hospital. PLoS One 2020;15:e0237159. 10.1371/journal.pone.0237159 32760101 PMC7410244

[19] Naugler C , Hemmelgarn B , Quan H , et al . Implementation of an intervention to reduce population-based screening for vitamin D deficiency: a cross-sectional study. CMAJ Open 2017;5:E36–9. 10.9778/cmajo.20160073 PMC5378529 28401116

[20] Colla CH , Morden NE , Sequist TD , et al . Choosing wisely: prevalence and correlates of low-value health care services in the United States. J Gen Intern Med 2015;30:221–8. 10.1007/s11606-014-3070-z 25373832 PMC4314495

[21] Mafi JN , Reid RO , Baseman LH , et al . Trends in low-value health service use and spending in the US Medicare fee-for-service program, 2014-2018. JAMA Netw Open 2021;4:e2037328. 10.1001/jamanetworkopen.2020.37328 33591365 PMC7887655

[22] Zorginstituut Nederland . Screeningsrapport Endocriene Ziekten; voedings- en stofwisselingstoornissen. Zorginstituut Nederland, 2018.

[23] Gonzalez-Chica D , Stocks N . Changes to the frequency and appropriateness of vitamin D testing after the introduction of new Medicare criteria for rebates in Australian general practice: evidence from 1.5 million patients in the NPS Medicineinsight database. BMJ Open 2019;9:e024797. 10.1136/bmjopen-2018-024797 PMC6429877 30852539

[24] Dutch Hospital Data . Landelijke Basisregistratie Zorg (LBZ). Data DH,

[25] The Netherlands Institute for Social Research . The Netherlands Institute for Social Research (SCP). Netherlands: Statusscores, 2017.

[26] Chalmers K , Pearson SA , Elshaug AG . Quantifying low-value care: a patient-centric versus service-centric lens. BMJ Qual Saf 2017;26:855–8. 10.1136/bmjqs-2017-006678 28842517

[27] McMaughan DJ , Oloruntoba O , Smith ML . Socioeconomic status and access to Healthcare: interrelated drivers for healthy aging. Front Public Health 2020;8:231. 10.3389/fpubh.2020.00231 32626678 PMC7314918

[28] Arpey NC , Gaglioti AH , Rosenbaum ME . How socioeconomic status affects patient perceptions of health care: A qualitative study. J Prim Care Community Health 2017;8:169–75. 10.1177/2150131917697439 28606031 PMC5932696

[29] Asch SM , Kerr EA , Keesey J , et al . Who is at greatest risk for receiving poor-quality health care N Engl J Med 2006;354:1147–56. 10.1056/NEJMsa044464 16540615

[30] David JL , Kaplan HB . Gender, social roles and health care utilization. Applied Behavioral Science Review 1995;3:39–64. 10.1016/S1068-8595(95)80012-3

[31] Bertakis KD , Azari R , Helms LJ , et al . Gender differences in the utilization of health care services. J Fam Pract 2000;49:147–52. 10718692

[32] Segal JB , Sen AP , Glanzberg-Krainin E , et al . Factors associated with Overuse of health care within US health systems: A cross-sectional analysis of Medicare beneficiaries from 2016 to 2018. JAMA Health Forum 2022;3:e214543. 10.1001/jamahealthforum.2021.4543 35977230 PMC8903118

[33] Snijders TAB , Bosker RJ . Multilevel Analysis: An Introduction to Basic and Advanced Multilevel. Modeling: SAGE Publications, 2011.

[34] Holick MF . Vitamin D deficiency. N Engl J Med 2007;357:266–81. 10.1056/NEJMra070553 17634462

[35] Green R , Miller JW . Chapter fifteen - vitamin B12 deficiency. In: Litwack G , ed. Vitamins and Hormones. 119: Academic Press, 2022: 405–39. 10.1016/bs.vh.2022.02.003 35337628

[36] Rebelos E , Tentolouris N , Jude E . The role of vitamin D in health and disease: A narrative review on the mechanisms linking vitamin D with disease and the effects of supplementation. Drugs 2023;83:665–85. 10.1007/s40265-023-01875-8 37148471 PMC10163584

[37] Halczuk K , Kaźmierczak-Barańska J , Karwowski BT , et al . Vitamin B12 — Multifaceted in vivo functions and in vitro applications. Nutrients 2023;15:2734. 10.3390/nu15122734 37375638 PMC10305463

[38] Knopman DS , DeKosky ST , Cummings JL , et al . Practice parameter: diagnosis of dementia (an evidence-based review). Neurology 2001;56:1143–53. 10.1212/WNL.56.9.1143 11342678

[39] Marcotte LM , Schuttner L , Liao JM . Measuring low-value care: learning from the US experience measuring quality. BMJ Qual Saf 2020;29:154–6. 10.1136/bmjqs-2019-010191 31649163

[40] Statistics Netherlands (CBS) [Zorginstellingen; kerngetallen]. 2019.

[41] Diagnostiek voor u . Fares 2022-2023. 2023. Available: https://diagnostiekvooru.nl/tarieven

[42] Vugt S van , de Schepper E , van Delft S , et al . Effectiveness of professional and patient-oriented strategies in reducing vitamin D and B12 test ordering in primary care: a cluster randomised intervention study. BJGP Open 2021;5:BJGPO.2021.0113. 10.3399/BJGPO.2021.0113 34407963 PMC9447297

[43] Itkonen ST , Andersen R , Björk AK , et al . Vitamin D status and current policies to achieve adequate vitamin D intake in the Nordic countries. Scand J Public Health 2021;49:616–27. 10.1177/1403494819896878 31916497

[44] Jääskeläinen T , Itkonen ST , Lundqvist A , et al . The positive impact of general vitamin D food Fortification policy on vitamin D status in a representative adult Finnish population: evidence from an 11-Y follow-up based on standardized 25-Hydroxyvitamin D data. Am J Clin Nutr 2017;105:1512–20. 10.3945/ajcn.116.151415 28490516

[45] Das JK , Salam RA , Mahmood SB , et al . Food Fortification with multiple Micronutrients: impact on health outcomes in general population. Cochrane Database Syst Rev 2019;12:CD011400. 10.1002/14651858.CD011400.pub2 31849042 PMC6917586

