# Supplementary material for: Non-indicated vitamin B12- and D-testing among Dutch hospital clinicians: a cross-sectional analysis in data registries
Source: BMJ Open. 2024 Feb 28;14(2):e075241. doi: 10.1136/bmjopen-2023-075241 (PMC10910490; doi:10.1136/bmjopen-2023-075241)
Supplement: Supplementary data [file bmjopen-2023-075241supp001.pdf]

Supplementary file S1: Included International classification of disease (ICD10) codes and subsequent diagnose-treatment combination (DTC) codes.

The table below contains the codes which were used to distinguish indicated from non-indicated vitamin B12- and D-tests. In case of vitamin D-testing, tests were considered to be appropriate in patients older than 50 with a fracture. We therefore have also provided a table of the fractures that were included.

Vitamin B12			
**ICD-10 code**	**Description**	**DTC code**	**DTC-Diagnosis description**
D51	**Vitamin B_{12} deficiency anaemia**<br>*Excl.: Vitamin B_{12} deficiency (E53.8)*	- 313 – 701<br>- 313 – 702<br>- 313 – 709<br>- 316 – 6001<br>- 316 – 6003<br>- 335 – 232	- Iron-deficiency anemia (not specified)<br>- Pernicious anemia<br>- Other erytrocital deviations (not specified)<br>- Anemia, Iron-deficiency<br>- Anemia, remaining<br>- Anemia
D52	**Folate deficiency anaemia**		
D53	**Other nutritional anaemias**<br>*Incl.: megaloblastic anaemia unresponsive to vitamin B_{12} or folate therapy*		
E53.8	**Deficiency of other specified B group vitamins**<br>*Deficiency:*<br>• *Biotin*<br>• *Cyanocobalamin*<br>• *Folate*<br>• *Folic acid*<br>• *Panthothenic acid*<br>• *Vitamin B12*	- 318 – 207<br>- 330 – 301	- (mal)nutrition<br>- Vitamin-deficiencies
E56.8	**Deficiency of other vitamins**		
E56.9	**Vitamin deficiency, unspecified**		
G63.4	**Polyneuropathy in nutritional deficiency**	- 327 – 413<br>- 330 – 312<br>- 330 – 812<br>- 335 – 251	- Pheripheral nervedamage, nerve disorder<br>- Neurological complication systemic disease<br>- Polyneuropathy, other<br>- Diseases of the nerve system and senses
R20.2	**Paraesthesia of skin**<br>*Excl.: acroparaesthesia (I73.8)*		
R27.0	**Ataxia, unspecified**		
K29.4	**Chronic atrophic gastritis**	- 313 – 916<br>- 318 – 401	- Erosive gastritis and duodenitis<br>- Gastritis, miscellaneous
K50	**Crohn disease [regional enteritis]**<br>*Incl.: granulomatous enteritis*<br>*Excl.: ulcerative colitis (K51.-)*	- 303 – 326<br>- 313 – 922<br>- 318 – 601<br>- 316 - 3314	- Crohn disease (enteritis regionalis)<br>- Crohn disease<br>- Morbus Crohn<br>- Inflammatory bowldisease (colitis ulcerosa/Morbus crohn)
K52	**Other noninfective gastroenteritis and colitis**	- 313 – 932<br>- 313 – 938	- Chronic diarrhoea without infection<br>- Radiation enteritis
K90	**Intestinal malabsorption**<br>*Excl.”following gastrointestinal surgery (K91.2)*	- 318 - 409	- Coeliac disease
Z98.0	**Internal bypass and anastomosis status**	- 303 – 341<br>- 303 – 342	- Morbid obesity BMI < 45<br>- Morbid obesity BMI > 45

**Vitamin D**			
**ICD-10 code**	**Description**	**DTC code**	**DTC-Diagnosis description**
E20<br>E21	**Hypoparathyroidism**<br>**Hyperparathyroidism and other disorders of parathyroid gland**	- 307 – E00<br>- 307 – E11<br>- 313 – 231<br>- 313 – 232<br>- 316 – 7110<br>- 316 – 7199<br>- 362 – 304	- Emergency consult Endocrinology<br>- Endocrinology<br>- Hyperparathyroidism<br>- Hypoparathyroidism<br>- Small body length/deviant growth curve<br>- Endocrinology, not specified<br>- Parathyroid glands
E83.5	**Disorders of calcium metabolism**	- 313 – 239<br>- 313 – 299<br>- 316 - 7503<br>- 330 - 399	- Calcium metabolic disease, unspecified<br>- Endocrine and metabolic diseases, unspecified<br>- Metabolic disease<br>- Deficiencies, metabolic, nutritional, unspecified
E84.9	**Cystic fibrosis, unspecified**	- 316 – 3205<br>- 322 - 1403	- Cystic fibrosis<br>- Cystic fibrosis
E55	**Vitamin D deficiency**<br>*Excl.: adult osteomalacia (M83.-), osteoporosis (M80-M81), sequelae of rickets (E64.3)*	- 305 – 1062<br>- 335 – 221<br>- 318 – 207<br>- 335 – 224	- Rachitis and/or vitamin D resistance osteomalacia<br>- Endocrine and nutrition disorders<br>- (mal)nutrition<br>- Calory/protein malnutrition
M83	**Adult osteomalacia**<br>*Excl.: osteomalacia:*<br>• *infantile and juvenile (E55.0)*<br>• *vitamin-D-resistant (E83.3)*<br>*Renal osteodystrophy (N25.0)*<br>*Rickets (active) (E55.0)*<br>*Rickets (active)*<br>• *sequelae (E64.3)*<br>*vitamin-D-resistant (E83.3)*		
E83.3	**Disorders of phosphorus metabolism and phosphates**<br>Acid phosphatase deficiency<br>Familial hypophosphataemia<br>Hypophosphatasia<br>Vitamin-D-resistant:<br>• Osteomalacia<br>• rickets<br>*Excl.: adult osteomalacia (M83.-), osteoporosis (M80-M81)*	- 316 – 3328<br>- 316 – 7503	- Food related problems/disorders<br>- Metabolic disease
K50	**Crohn disease [regional enteritis]**<br>*Incl.: granulomatous enteritis*<br>*Excl.: ulcerative colitis (K51.-)*	- 303 – 326<br>- 313 – 922<br>- 318 – 601	- Crohn disease (enteritis regionalis)<br>- Crohn disease<br>- Morbus Crohn
K90	**Intestinal malabsorption**<br>*Excl."following gastrointestinal surgery (K91.2)*	- 313 – 920<br>- 318 – 409	- Coeliac disease / malabsorption<br>- Coeliac disease
M80	**Osteoporosis with pathological fracture**<br>*Incl.: osteoporotic vertebral collapse and wedging*<br>*Excl.: collapsed vertebra NOS (M48.5), pathological fracture NOS (M84.4), wedging of vertebra NOS (M48.5)*	- 305 – 1395<br>- 316 – 5105	- Osteoperotic collapse<br>- Pathological fractures

M81	**Osteoporosis without pathological fracture**<br>*Excl.: osteoporosis with pathological fracture (M80.-)*	- 313 – 233	- Osteoperosis, osteomalacia
M82	**Osteoporosis in diseases classified elsewhere**		
N25.0	**Renal osteodystrophy**<br>*Azotaemic osteodystrophy*<br>*Phosphate-losing tubular disorders*<br>*Renal:*<br>• *rickets*<br>• *short stature*	- 313 – 399<br>- 313 – 325	- Renal diseases, miscellaneous<br>- Chronic renal disease (eGFR <30 ml/min)

Additional DTC codes added after checking the resulting indications:

D	0316	3304	Celiac disease	6509
D	0316	4006	Chronic renal failure	1245
D	0318	753	Chronic pancreatitis	3415
B12	0316	3304	Celiac disease	6233
B12	0313	772	Polycythemia vera, essential trombocytosis	5434
B12	0313	763	Myelodysplastic syndrome no specified	4843
B12	0313	920	Celiac disease/malabsorption	4206
B12	0313	773	Chronic myelogenous leukemia (CMMoL)	312

Bone fractures above 50 included in our analysis, that justify the ordering of a vitamin D-test.

DTC specialism code	DTC diagnose code	Fracture description.
0305	3011	Radius head
0305	3014	Carpus
0305	3029	Metatarsal bones
0303	207	Humerus proximal and shaft
0305	3024	Tibia (with or without fibula, excluding ankle)
0303	210	Radius head
0304	451	Fracture of distal radius surgical
0303	219	Femur, remaining
0303	221	Tibial plateau
0303	222	Tibia (± fibula, excluding ankle)
0305	3023	Tibial plateau
0303	217	Pelvis / sacrum
0303	214	Metacarpal bones
0305	3008	Humerus proximal and shaft
0303	224	Ankle
0305	3026	Calcaneus
0305	3030	Phalanges of the feet
0308	2301	Trauma vertebral column: conservative treatment
0303	241	Talus
0305	3020	Femur, remaining
0303	237	Tarsus
0303	215	Phalanges of the hand
0305	3027	Talus
0304	420	Fracture / luxation carpalia conservative
0305	3017	Pelvis
0305	3005	Vertebral column with paraplegia
0303	220	Patella
0303	238	Metatarsal bones
0305	3004	Vertebral column
0305	3009	Distal humerus/(epi)condyl(len)
0305	3015	Metacarpal bones
0305	3022	Fibula
0305	3025	Ankle
0303	239	Phalanges of the feet
0303	209	Olecranon
0303	213	Carpus
0305	3013	Wrist
0308	2311	Trauma vertebral column: Surgical intervention including instrumentation c.q. spinal fusion
0304	450	Distal radius facture conservative
0304	8418	Traumatic Vertebral compression fracture
0303	211	Forearm, no further specification
0305	3021	Patella
0304	421	Fracture / luxation carpalia operative
0303	212	Wrist
0303	208	Distal humerus / (epi)condyl(en)
0303	203	Vertebral column
0303	204	Vertebral column with spinal lesion
0308	2305	Treatment of trauma to the vertebral column with external fixation or traction
0305	3010	Olecranon

0305	3028	Tarsus
0303	236	Calcaneus
0305	3012	Forearm
0305	3016	Phalanges of the hand
0303	218	Fracture of neck of femur (disorder)
0305	3019	Femur proximaal (+ collum)
