# Supplementary material for: Non-indicated vitamin B12- and D-testing among Dutch hospital clinicians: a cross-sectional analysis in data registries
Source: BMJ Open. 2024 Feb 28;14(2):e075241. doi: 10.1136/bmjopen-2023-075241 (PMC10910490; doi:10.1136/bmjopen-2023-075241)
Supplement: Supplementary data [file bmjopen-2023-075241supp002.pdf]

Supplementary file S2: overview calculated generalized variance inflation factors (GVIF).

Generalized Variation Inflation Factors (GVIF) calculated for the models made using DHD data.

Year	Vitamin test	Variable	GVIF	GVIF^(1/(2*DF))
2015	Vitamin B12	*Gender*	1.038	1.019
		Age category	1.040	1.010
		Socio-economic status category	1.017	1.0021
		Hospital size	1.016	1.0041
	Vitamin D	*Gender*	1.013	1.0065
		Age category	1.016	1.0039
		Socio-economic status category	1.029	1.0035
		Hospital size	1.028	1.0069
2016	Vitamin B12	*Gender*	1.036	1.018
		Age category	1.037	1.0092
		Socio-economic status category	1.01	1.0017
		Hospital size	1.013	1.0033
	Vitamin D	*Gender*	1.012	1.0060
		Age category	1.013	1.0033
		Socio-economic status category	1.019	1.0024
		Hospital size	1.020	1.0050
2017	Vitamin B12	*Gender*	1.035	1.017
		Age category	1.036	1.0089
		Socio-economic status category	1.017	1.0021
		Hospital size	1.017	1.0043
	Vitamin D	*Gender*	1.013	1.0064
		Age category	1.014	1.0037
		Socio-economic status category	1.023	1.0028
		Hospital size	1.023	1.0057
2018	Vitamin B12	*Gender*	1.038	1.019
		Age category	1.040	1.0097
		Socio-economic status category	1.025	1.0031
		Hospital size	1.024	1.0060
	Vitamin D	*Gender*	1.016	1.0078
		Age category	1.018	1.0044
		Socio-economic status category	1.029	1.0036
		Hospital size	1.030	1.0073
2019	Vitamin B12	*Gender*	1.035	1.0175
		Age category	1.037	1.0091
		Socio-economic status category	1.035	1.0043
		Hospital size	1.035	1.0085
	Vitamin D	*Gender*	1.015	1.0072
		Age category	1.017	1.0041
		Socio-economic status category	1.040	1.0049
		Hospital size	1.040	1.0098
