# Supplementary material for: Non-indicated vitamin B12- and D-testing among Dutch hospital clinicians: a cross-sectional analysis in data registries
Source: BMJ Open. 2024 Feb 28;14(2):e075241. doi: 10.1136/bmjopen-2023-075241 (PMC10910490; doi:10.1136/bmjopen-2023-075241)
Supplement: Supplementary data [file bmjopen-2023-075241supp003.pdf]

Supplementary file S3: Extended table 1; distribution of vitamin B12- and D-testing across the sex, age and SES categories.

Table 1: extended table 1 for vitamin B12.

1. Vitamin B12																
		**2015**			**2016**			**2017**			**2018**			**2019**		
	No. of hospitals included	63			64			66			69			68		
	Total no. of unique patients with (at least) one vitamin B12 test	233,541			226,999			239,033			240,063			239,351		
	Total no. of unique patients with (at least) one vitamin B12 test with an associated DBC code	103,540			141,507			162,900			178,933			183,204		
	Total no. of unique patients with (at least) one non-indicated vitamin B12 test	83,549			111,986			128,872			141,914			145,039		
		**Total**	**Without (clear) indication**	**% without (clear indication**	**Total**	**Without (clear) indication**	**% without (clear indication**	**Total**	**Without (clear) indication**	**% without (clear indication**	**Total**	**Without (clear) indication**	**% without (clear indication**	**Total**	**Without (clear) indication**	**% without (clear indication**
**Male**	**No. of male patients receiving at least one vitamin-B12 test**	38,308	31,731	82.8	52,360	42,758	81.7	60,511	49,222	81.3	66,724	54,567	81.8	67,869	55,148	81.3
Age cat.	**18-29**	3,152	2,461	78.1	4,473	3,421	76.5	5,262	4,094	77.8	5,921	4,579	77.3	6,468	4,886	75.5
	**30-49**	9,785	7,967	81.4	13,346	10,598	79.4	15,247	12,119	79.5	16,397	13,175	80.4	16,864	13,572	80.5
	**50-69**	25,371	21,303	84.0	34,541	28,739	83.2	40,002	33,009	82.5	44,406	36,813	82.9	44,537	36,690	82.4
**Neighbou**	***Lowest socio-economic status****	8,070	6,693	82.9	11,346	9,271	81.7	13,512	11,082	82.0	14,632	12,008	82.1	15,099	12,346	81.8
**SES cat.**	***Socio-economic status below average***	8,484	7,022	82.8	11,347	9,236	81.4	13,601	11,078	81.4	14,686	12,001	81.7	14,962	12,116	81.0
	Average socio-economic status	7,790	6,472	83.1	10,372	8,472	81.7	11,669	9,447	81.0	13,296	10,908	82.0	13,086	10,593	80.9
	Socio-economic status above average	8,067	6,698	83.0	10,481	8,576	81.8	11,738	9,480	80.8	12,914	10,527	81.5	13,150	10,687	81.3
	Highest socio-economic status	5,639	4,625	82.0	8,426	6,860	81.4	9,564	7,766	81.2	10,666	8,665	81.2	10,985	8,915	81.2
	Missing	258	221	85.7	388	343	88.4	427	369	86.4	530	458	86.4	587	491	83.6
**Female**	**No. of female patients receiving at least one vitamin-B12 test**	65,232	51,818	79.4	89,147	69,228	77.7	102,389	79,650	77.8	112,209	87,347	77.8	115,335	89,891	77.9
Age cat.	**18-29**	9,138	7,155	78.3	12,781	9,751	76.3	14,863	11,415	76.8	16,799	13,011	77.5	17,371	13,487	77.6
	**30-49**	24,905	18,991	76.3	34,030	25,313	74.4	38,672	28,996	75.0	42,186	31,649	75.0	43,276	32,826	75.9
	**50-69**	31,189	25,672	82.3	42,336	34,164	80.7	48,854	39,239	80.3	53,224	42,687	80.2	54,688	43,578	79.7
**Neighbou**	***Lowest socio-economic status****	14,351	11,223	78.2	19,935	15,267	76.6	23,709	18,370	77.5	25,526	19,832	77.7	26,005	20,190	77.6
**SES cat.**	***Socio-economic status below average***	14,520	11,513	79.3	19,865	15,328	77.2	23,072	17,820	77.2	25,157	19,555	77.7	25,860	20,112	77.8
	Average socio-economic status	13,035	10,453	80.2	17,656	13,737	77.8	19,669	15,213	77.3	21,936	17,000	77.5	22,334	17,366	77.8
	Socio-economic status above average	13,122	10,459	79.7	17,006	13,245	77.9	19,098	14,899	78.0	21,366	16,625	77.8	21,984	17,079	77.7
	Highest socio-economic status	9,850	7,871	79.9	14,226	11,277	79.3	16,294	12,914	79.3	17,601	13,813	78.5	18,532	14,643	79.0
	Missing	354	299	84.5	459	374	81.5	547	434	79.3	623	522	83.8	620	501	80.8

Table 1: extended table 1 for vitamin D.

c																
		**2015**			**2016**			**2017**			**2018**			**2019**		
	No. of hospitals included	62			61			63			65			65		
	Total no. of patients with (at least) one vitamin D test	244,834			247,186			264,811			265,362			272,380		
	Total no. of patients with (at least) one vitamin D test with an associated DBC code	101,932			148,660			172,342			189,423			196,452		
	Total no. of patients with (at least) one non-indicated vitamin D test	84,414			124,162			143,408			157,431			162,345		
		**Total**	**Without (clear) indication**	**% without (clear indication**	**Total**	**Without (clear) indication**	**% without (clear indication**	**Total**	**Without (clear) indication**	**% without (clear indication**	**Total**	**Without (clear) indication**	**% without (clear indication**	**Total**	**Without (clear) indication**	**% without (clear indication**
**Male**	**No. of male patients receiving at least one vitamin-D test**	31,660	26,363	83.3	47,794	40,211	84.1	56,211	47,313	84.2	61,749	51,845	84.0	63,295	52,656	83.2
Age cat.	**18-29**	2,689	2,220	82.6	4,201	3,426	81.6	5,185	4,179	80.6	5,992	4,779	79.8	6,529	5,052	77.4
	**30-49**	8,684	7,658	88.2	13,086	11,536	88.2	15,614	13,701	87.7	16,964	14,750	86.9	17,483	15,123	86.5
	**50-69**	20,287	16,485	81.3	30,507	25,249	82.8	35,412	29,433	83.1	38,793	32,316	83.3	39,283	32,481	82.7
**Neighbou**	***Lowest socio-economic status****	6,358	5,326	83.8	10,349	8,813	85.2	12,430	10,559	84.9	13,295	11,272	84.8	13,578	11,323	83.4
**SES cat.**	***Socio-economic status below average***	6,758	5,584	82.6	9,964	8,342	83.7	12,199	10,201	83.6	13,249	11,079	83.6	13,818	11,496	83.2
	Average socio-economic status	6,331	5,233	82.7	9,311	7,755	83.3	10,677	8,851	82.9	12,292	10,249	83.4	12,287	10,092	82.1
	Socio-economic status above average	6,855	5,746	83.8	9,476	7,955	83.9	10,833	9,090	83.9	11,678	9,739	83.4	12,134	10,134	83.5
	Highest socio-economic status	5,164	4,309	83.4	8,405	7,092	84.4	9,735	8,315	85.4	10,825	9,151	84.5	11,025	9,238	83.8
	Missing	194	165	85.1	289	254	87.9	337	297	88.1	410	355	86.6	453	373	82.3
**Female**	**No. of female patients receiving at least one vitamin-D test**	70,272	58,051	82.6	100,866	83,951	83.2	116,131	96,095	82.7	127,674	105,586	82.7	133,157	109,689	82.4
Age cat.	**18-29**	8,105	7,198	88.8	11,485	10,030	87.3	14,038	12,120	86.3	16,091	13,864	86.2	17,271	14,855	86.0
	**30-49**	25,045	22,802	91.0	35,377	32,119	90.8	40,982	37,028	90.4	45,932	41,407	90.1	47,884	42,896	89.6
	**50-69**	37,122	28,051	75.6	54,004	41,802	77.4	61,111	46,947	76.8	65,651	50,315	76.6	68,002	51,938	76.4
**Neighbou**	***Lowest socio-economic status****	15,024	12,710	84.6	22,104	18,873	85.4	26,254	22,286	84.9	28,219	23,903	84.7	29,182	24,613	84.3
**SES cat.**	***Socio-economic status below average***	15,087	12,344	81.8	21,509	17,770	82.6	25,087	20,641	82.3	27,646	22,868	82.7	28,971	23,760	82.0
	Average socio-economic status	13,987	11,419	81.6	19,561	16,032	82.0	22,374	18,184	81.3	24,947	20,169	80.8	25,582	20,690	80.9
	Socio-economic status above average	14,302	11,739	82.1	19,405	15,976	82.3	21,663	17,576	81.1	24,007	19,542	81.4	25,303	20,528	81.1
	Highest socio-economic status	11,581	9,608	83.0	17,823	14,923	83.7	20,248	17,011	84.0	22,235	18,618	83.7	23,500	19,606	83.4
	Missing	291	231	79.4	464	377	81.3	505	397	78.6	620	486	78.4	619	492	79.5
