# Supplementary material for: Non-indicated vitamin B12- and D-testing among Dutch hospital clinicians: a cross-sectional analysis in data registries
Source: BMJ Open. 2024 Feb 28;14(2):e075241. doi: 10.1136/bmjopen-2023-075241 (PMC10910490; doi:10.1136/bmjopen-2023-075241)
Supplement: Supplementary data [file bmjopen-2023-075241supp004.pdf]

Supplementary file S4: top 20 diagnose codes associated with non-indicated vitamin B12- and D-testing.

Table 1: Top 20 of diagnose codes associated with non-indicated vitamin B12-tests.

No. of non-indicated vitamin B12-tests	DTC specialism code	DTC diagnose code	DTC specialism description	DTC diagnose description
31,055	313	3	Internal medicine	Analysis general malaise/fatigue without diagnosis
30,807	313	222	Internal medicine	Diabetes mellitus with secondary complications
26,591	313	283	Internal medicine	Adiposity (obesity)
25,268	318	602	Gastro-enterology	Ulcerative colitis
20,667	313	221	Internal medicine	Diabetes mellitus without secondary complications
20,118	307	Z41	Obstetrics and gynaecology	Miscellaneous counselling during pregnancy
18,747	318	205	Gastro-enterology	Irritable bowel syndrome, diverticulosis
12,136	313	211	Internal medicine	Hypothyroidism
11,765	330	531	Neurology	Multiple sclerosis
11,168	313	6	Internal medicine	Analysis stomach complaints without diagnosis
10,003	313	223	Internal medicine	Diabetes mellitus chronic pump therapy
8,358	318	203	Gastro-enterology	Blood loss Gastrointestinal tract, unknown cause
8,057	324	701	Rheumatology	Arthralgia and/or myalgia
7,822	313	4	Internal medicine	Analysis anorexia, emaciation without diagnosis
7,770	318	204	Gastro-enterology	Chronic stomach-ache
7,425	330	402	Neurology	Miscellaneous cognitive and memory impairments
6,841	313	311	Internal medicine	Hypertension
6,758	324	101	Rheumatology	Rheumatoid arthritis
6,688	313	324	Internal medicine	Chronic kidney insufficiency eGFR 30-60 ml/min
6,339	313	299	Internal medicine	Miscellaneous endocrine and metabolic conditions

Table 2: Top 20 of diagnose codes associated with non-indicated vitamin D-tests

No. of non-indicated vitamin D-tests	DTC specialism code	DTC diagnose code	DTC specialism description	DTC diagnose description
38,213	303	342	General surgery	Morbid obesity BMI >45
34,842	313	283	Internal medicine	Adiposity (obesity)
30,175	307	Z41	Obstetrics and gynaecology	Miscellaneous counselling during pregnancy
29,857	324	701	Rheumatology	Arthralgia and/or myalgia
25,072	318	602	Gastro-enterology	Ulcerative colitis
23,263	313	3	Internal medicine	Analysis general malaise/fatigue without diagnosis
21,723	313	222	Internal medicine	Diabetes mellitus with secondary complications
21,076	324	101	Rheumatology	Rheumatoid arthritis
19,013	313	811	Internal medicine	Malignancy mama
18,346	330	531	Neurology	Multiple sclerosis
17,208	313	324	Internal medicine	Chronic kidney insufficiency eGFR 30-60 ml/min
15,050	313	461	Internal medicine	HIV infection with treatment indication
12,718	303	341	General surgery	Morbid obesity BMI <45
12,354	313	76	Internal medicine	Recipient kidney transplant
12,155	324	707	Rheumatology	Fibromyalgia
12,025	313	221	Internal medicine	Diabetes mellitus without secondary complications
11,182	313	211	Internal medicine	Hypothyroidism
10,926	313	311	Internal medicine	Hypertension
9,960	322	1201	Respiratory medicine	Asthma
8,639	318	205	Gastro-enterology	Irritable bowel syndrome, diverticulosis
