# Supplementary material for: Non-indicated vitamin B12- and D-testing among Dutch hospital clinicians: a cross-sectional analysis in data registries
Source: BMJ Open. 2024 Feb 28;14(2):e075241. doi: 10.1136/bmjopen-2023-075241 (PMC10910490; doi:10.1136/bmjopen-2023-075241)
Supplement: Supplementary data [file bmjopen-2023-075241supp005.pdf]

Supplementary file S5: Hospital variation; overview of Intraclass correlation coefficient across years (ICC) of both the casemix adjusted and unadjusted models for vitamin B12- and D-testing included in our analysis (2015 – 2019).

Table 3: Hospital variation; overview of Intraclass correlation coefficient across years (ICC) of both the casemix adjusted and unadjusted models for vitamin B12- and D-testing included in our analysis (2015 – 2019). Patient age, gender, socioeconomic status and an proxy for hospital size were included as casemix variables in the adjusted models.

	2015		2016		2017		2018		2019	
	Unadjusted	*Adjusted*	*Unadjusted*	*Adjusted*	*Unadjusted*	*Adjusted*	*Unadjusted*	*Adjusted*	*Unadjusted*	*Adjusted*
**Vitamin-B12**	0.083	0.072	0.094	0.085	0.088	0.081	0.089	0.079	0.095	0.082
**Vitamin-D**	0.101	0.095	0.096	0.083	0.085	0.081	0.095	0.090	0.099	0.095
