# Supplementary material for: Non-indicated vitamin B12- and D-testing among Dutch hospital clinicians: a cross-sectional analysis in data registries
Source: BMJ Open. 2024 Feb 28;14(2):e075241. doi: 10.1136/bmjopen-2023-075241 (PMC10910490; doi:10.1136/bmjopen-2023-075241)
Supplement: Supplementary data [file bmjopen-2023-075241supp006.pdf]

Supplementary file S6: overview of the raw data and proportions of the total outpatient visits that received a vitamin B12- and D- test for each of the included hospitals over 2019.

Table 1: Proportion (%) of outpatient visits that received a vitamin B12-tests without a clear indication,

No,	Hospital ID	Total no, of outpatient visits recorded in 2019	Total no, of vitamin B12 tests without a clear indication	Proportion (%) of outpatient visits that received a vitamin B12 test without clear indication
1	48	362,887	2	0.00
2	40	580,678	8	0.00
3	66	127,984	52	0.04
4	41	576,063	263	0.05
5	11	345,279	407	0.12
6	23	568,860	716	0.13
7	38	289,822	400	0.14
8	39	565,276	1,094	0.19
9	46	358,602	1,693	0.47
10	1	344,543	1,953	0.57
11	62	227,934	1,324	0.58
12	25	165,640	985	0.59
13	50	436,838	2,808	0.64
14	52	508,460	4,723	0.93
15	35	156,620	1,485	0.95
16	68	133,631	1,387	1.04
17	12	494,601	6,081	1.23
18	67	153,812	1,915	1.25
19	69	318,615	4,021	1.26
20	71	408,355	5,680	1.39
21	55	194,373	2,792	1.44
22	60	421,743	6,422	1.52
23	6	384,801	6,032	1.57
24	59	395,402	6,608	1.67
25	27	389,827	7,214	1.85

26	54	383,123	7,109	1.86
27	36	363,166	7,477	2.06
28	16	163,322	5,133	3.14
29	19	131,553	4,361	3.32
30	57	280,541	9,441	3.37
31	63	427,252	15,000	3.51
32	29	216,550	7,672	3.54
33	4	315,289	11,366	3.60
34	65	237,265	8,670	3.65
35	31	309,391	11,457	3.70
36	28	113,439	4,239	3.74
37	42	268,758	10,220	3.80
38	45	384,873	14,745	3.83
39	64	342,101	13,120	3.84
40	17	155,415	6,008	3.87
41	30	370,550	14,413	3.89
42	7	384,199	14,944	3.89
43	33	606,974	23,806	3.92
44	20	147,262	5,997	4.07
45	49	259,604	10,903	4.20
46	26	182,212	7,660	4.20
47	47	185,195	7,875	4.25
48	56	192,410	8,244	4.28
49	34	429,614	18,618	4.33
50	10	276,454	12,102	4.38
51	8	223,140	9,775	4.38
52	14	567,264	25,389	4.48
53	70	253,998	11,381	4.48
54	24	654,329	29,546	4.52
55	43	536,979	24,622	4.59
56	51	443,539	21,314	4.81
57	44	356,220	17,236	4.84

58	58	154,585	7,521	4.87
59	15	165,151	8,097	4.90
60	13	466,598	23,040	4.94
61	5	401,794	20,260	5.04
62	37	171,709	8,665	5.05
63	32	337,152	19,183	5.69
64	61	174,166	10,466	6.01
65	3	113,329	7,276	6.42
66	22	251,812	17,093	6.79
67	21	186,579	13,004	6.97
68	2	379,408	104,750	27.61

Table 2: Proportion (%) of outpatient visits that received a vitamin D tests without a clear indication,

No,	Hospital ID	Total no, of outpatient visits recorded in 2019	Total no, of vitamin D determinations without a clear indication	Proportion (%) of outpatient visits that received a vitamin D determination without clear indication
1	48	362,887	0	0.00
2	40	580,678	0	0.00
3	38	289,822	0	0.00
4	41	576,063	576,063	0.02
5	66	127,984	127,984	0.02
6	11	345,279	345,279	0.06
7	23	568,860	568,860	0.08
8	39	565,276	565,276	0.20
9	50	436,838	436,838	0.28
10	1	344,543	344,543	0.38
11	68	133,631	133,631	0.48
12	62	227,934	227,934	0.54
13	25	165,640	165,640	0.59
14	52	508,460	508,460	0.64
15	46	358,602	358,602	0.71
16	35	156,620	156,620	0.84
17	67	153,812	153,812	0.89
18	55	194,373	194,373	1.35
19	60	421,743	421,743	1.35
20	6	384,801	384,801	1.49
21	12	494,601	494,601	1.52
22	71	408,355	408,355	1.75
23	59	395,402	395,402	2.36
24	36	363,166	363,166	2.39
25	28	113,439	113,439	2.64
26	30	370,550	370,550	2.65
27	27	389,827	389,827	2.75

28	54	383,123	383,123	2.88
29	64	342,101	342,101	3.04
30	69	318,615	318,615	3.07
31	56	192,410	192,410	3.20
32	20	147,262	147,262	3.20
33	29	216,550	216,550	3.23
34	26	182,212	182,212	3.30
35	8	223,140	223,140	3.39
36	47	185,195	185,195	3.41
37	17	155,415	155,415	3.41
38	45	384,873	384,873	3.45
39	57	280,541	280,541	3.50
40	19	131,553	131,553	3.50
41	33	606,974	606,974	3.67
42	5	401,794	401,794	4.05
43	24	654,329	654,329	4.06
44	34	429,614	429,614	4.30
45	65	237,265	237,265	4.58
46	22	251,812	251,812	4.80
47	58	154,585	154,585	4.97
48	42	268,758	268,758	5.00
49	15	165,151	165,151	5.07
50	3	113,329	113,329	5.07
51	16	163,322	163,322	5.27
52	51	443,539	443,539	5.44
53	13	466,598	466,598	5.46
54	4	315,289	315,289	5.68
55	21	186,579	186,579	5.84
56	49	259,604	259,604	5.91
57	32	337,152	337,152	5.91
58	70	253,998	253,998	6.17
59	63	427,252	427,252	6.31

60	61	174,166	174,166	6.33
61	14	567,264	567,264	6.50
62	10	276,454	276,454	6.83
63	31	309,391	309,391	7.04
64	44	356,220	356,220	7.42
65	37	171,709	171,709	8.37
66	43	536,979	536,979	9.00
67	7	384,199	384,199	9.37
68	2	379,408	379,408	34.79
