# Supplementary material for: Non-indicated vitamin B12- and D-testing among Dutch hospital clinicians: a cross-sectional analysis in data registries
Source: BMJ Open. 2024 Feb 28;14(2):e075241. doi: 10.1136/bmjopen-2023-075241 (PMC10910490; doi:10.1136/bmjopen-2023-075241)
Supplement: Supplementary data [file bmjopen-2023-075241supp007.pdf]

Supplementary file S7: Density plots and Shapiro-Wilk normality test outcomes and the correlation test outcomes (with and without the outlierincluded)

Table 1: Normality testing outcomes

**Variable**	**Shapiro-Wilk normality test**	**P-value (significance p<0.05)**	**Outcome**
Rate of non-indicated vitamin-B12 tests	0.59	1.46e-12	Not normally distributed
Rate of non-indicated vitamin-D tests	0.58	1.19e -12	Not normally distributed

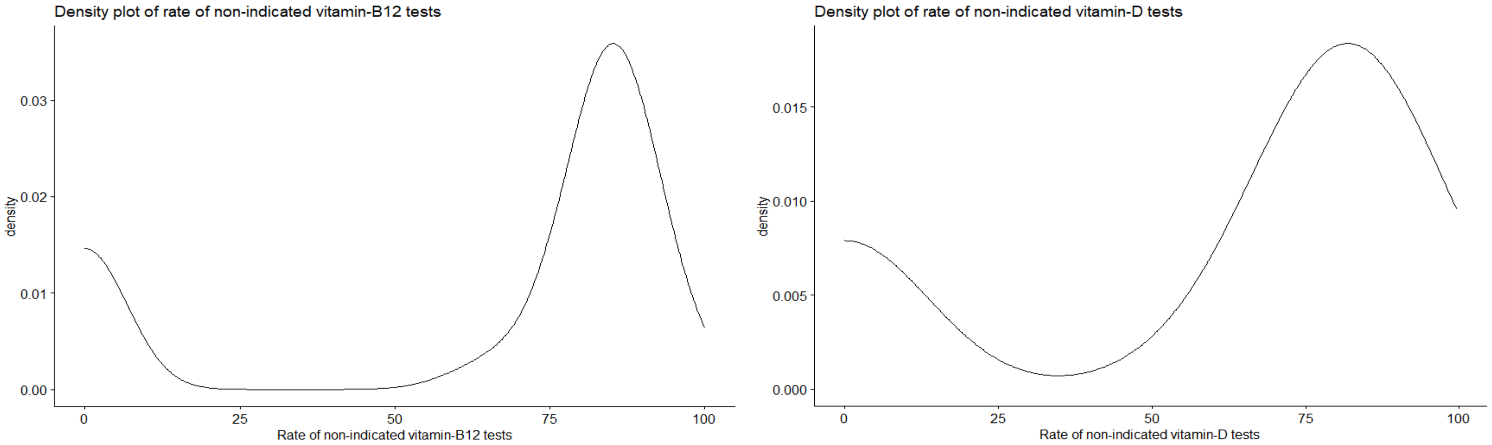

Figure 1: Density plots of rates of vitamin B12- and D-testing among the hospitals included

Table 2: Spearman's rank correlation Rho test outcomes

**Rho**	**P-value**
0.86	P < 2.2e-16

Correlation analysis outcome before removal of the outlier

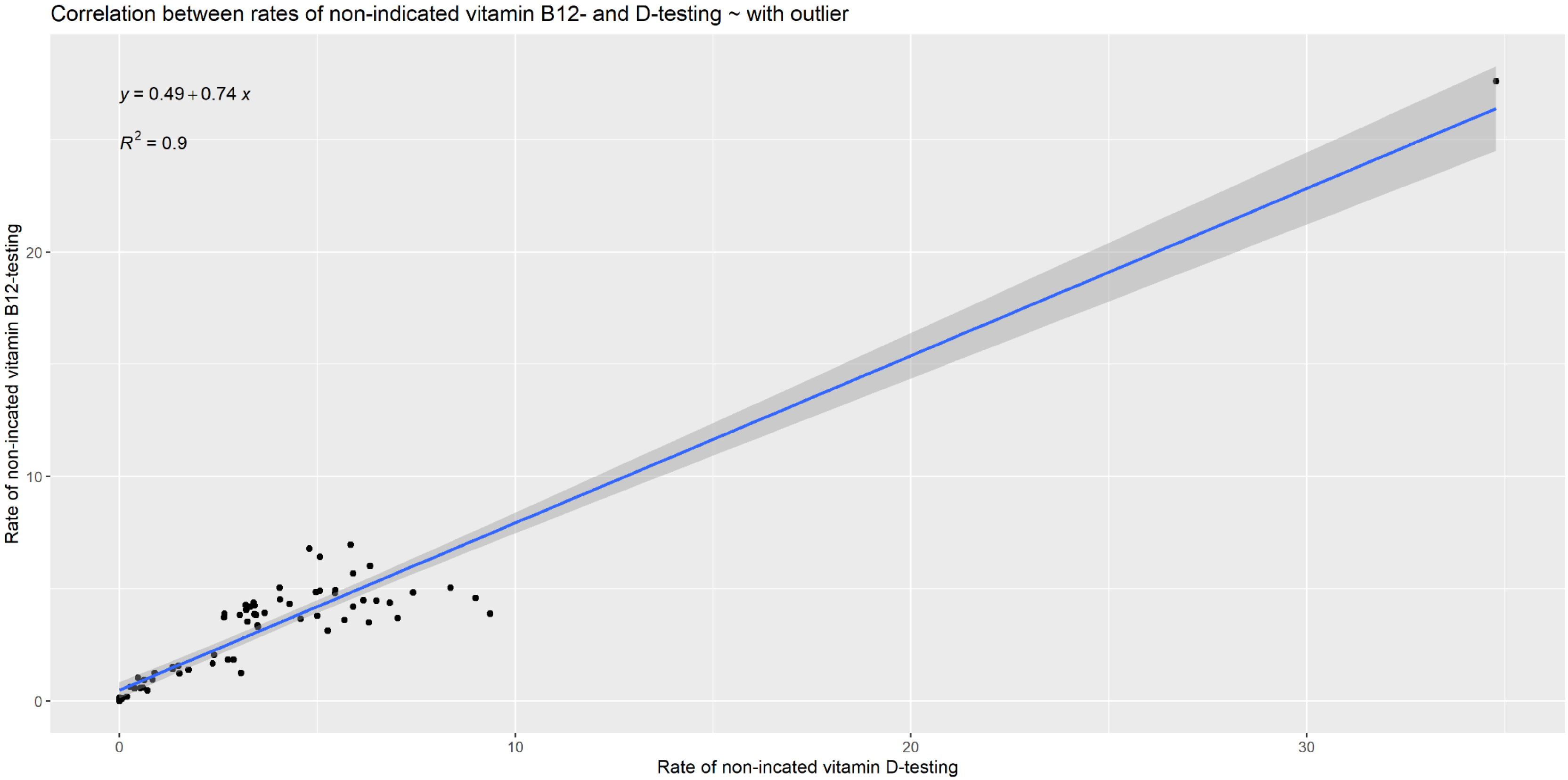

Figure 2: rates of non-indicated vitamin B12- and D-testing before removal of the outlier
