# Supplementary material for: Non-indicated vitamin B12- and D-testing among Dutch hospital clinicians: a cross-sectional analysis in data registries
Source: BMJ Open. 2024 Feb 28;14(2):e075241. doi: 10.1136/bmjopen-2023-075241 (PMC10910490; doi:10.1136/bmjopen-2023-075241)
Supplement: Author's manuscript [file bmjopen-2023-075241.draft_revisions.pdf]

BMJ Open is committed to open peer review. As part of this commitment we make the peer review history of every article we publish publicly available.

When an article is published we post the peer reviewers' comments and the authors' responses online. We also post the versions of the paper that were used during peer review. These are the versions that the peer review comments apply to.

The versions of the paper that follow are the versions that were submitted during the peer review process. They are not the versions of record or the final published versions. They should not be cited or distributed as the published version of this manuscript.

BMJ Open is an open access journal and the full, final, typeset and author-corrected version of record of the manuscript is available on our site with no access controls, subscription charges or pay-per-view fees (http://bmjopen.bmj.com).

If you have any questions on BMJ Open's open peer review process please email info.bmjopen@bmj.com

BMJ Open

Non-indicated vitamin B12- and D-testing among Dutch hospital clinicians: a cross-sectional cohort study

Journal:	*BMJ Open*
Manuscript ID	bmjopen-2023-075241
Article Type:	Original research
Date Submitted by the Author:	01-May-2023

Complete List of Authors:	Müskens, Joris; Radboudumc IQ healthcare, IQ healthcare<br>Kool, Rudolf; Radboud university medical centre, Scientific Institute for Quality of Healthcare (IQ Healthcare)<br>Westert, Gert; Radboud university medical centre, Scientific Institute for Quality of Healthcare (IQ Healthcare)<br>Zaal, Maarten; Information analyst at Dutch Hospital Data<br>Muller, Hein; Dutch Hospital Data<br>Atsma, Femke; Radboud University Nijmegen Medical Centre, Scientific Institute for Quality of Healthcare<br>van Dulmen, SA; Radboud University Medical Center, IQ healthcare

Keywords:	Quality in health care < HEALTH SERVICES ADMINISTRATION & MANAGEMENT, Hospitals, Public, Clinical Decision-Making, Retrospective Studies

SCHOLARONE™
Manuscripts

I, the Submitting Author has the right to grant and does grant on behalf of all authors of the Work (as defined in the below author licence), an exclusive licence and/or a non-exclusive licence for contributions from authors who are: i) UK Crown employees; ii) where BMJ has agreed a CC-BY licence shall apply, and/or iii) in accordance with the terms applicable for US Federal Government officers or employees acting as part of their official duties; on a worldwide, perpetual, irrevocable, royalty-free basis to BMJ Publishing Group Ltd ("BMJ") its licensees and where the relevant Journal is co-owned by BMJ to the co-owners of the Journal, to publish the Work in this journal and any other BMJ products and to exploit all rights, as set out in our licence.

The Submitting Author accepts and understands that any supply made under these terms is made by BMJ to the Submitting Author unless you are acting as an employee on behalf of your employer or a postgraduate student of an affiliated institution which is paying any applicable article publishing charge ("APC") for Open Access articles. Where the Submitting Author wishes to make the Work available on an Open Access basis (and intends to pay the relevant APC), the terms of reuse of such Open Access shall be governed by a Creative Commons licence – details of these licences and which Creative Commons licence will apply to this Work are set out in our licence referred to above.

Other than as permitted in any relevant BMJ Author's Self Archiving Policies, I confirm this Work has not been accepted for publication elsewhere, is not being considered for publication elsewhere and does not duplicate material already published. I confirm all authors consent to publication of this Work and authorise the granting of this licence.

For peer review only

Non-indicated vitamin B12- and D-testing among Dutch hospital clinicians: a cross-sectional cohort study

1. Joris L.J.M. Müskens, PhD student at Radboud Institute for Health Sciences, IQ healthcare, Radboud University Medical Centre, Nijmegen, The Netherlands
2. Rudolf B. Kool, PhD MD, Professor of Appropriate Care and program manager of "To do or not to do?" at Radboud Institute for Health Sciences, IQ healthcare, Radboud University Medical Centre, Nijmegen, The Netherlands
3. Gert P. Westert, PhD, Professor of Health services research and director of IQ healthcare at Radboud Institute for Health Sciences, IQ healthcare, Radboud University Medical Centre, Nijmegen, The Netherlands
4. Maarten Zaal, PhD, Information analyst at Dutch Hospital Data, Utrecht, The Netherlands
5. Hein Muller, MD, PhD MD, FACP
6. Femke Atsma, PhD, Senior epidemiologist and methodologist, IQ healthcare, Radboud University Medical Centre, Nijmegen, The Netherlands
7. Simone A. van Dulmen, PhD, senior researcher and project manager at Radboud Institute for Health Sciences, IQ healthcare, Radboud University Medical Centre, Nijmegen, The Netherlands

Corresponding author: Joris L.J.M. Müskens, Joris.muskens@radboudumc.nl

Postal address: Radboud Institute for Health Sciences, Scientific Center for Quality of Healthcare (IQ healthcare), Postbus 9101, 6500 HB Nijmegen (route 160), The Netherlands

Word count: 3,576

For peer review only

Abstract

Objectives: to assess the extent of non-indicated vitamin B12- and D-testing among Dutch clinicians and its variation among hospitals.

Design: retrospective cohort study using registration data from 2015-2019.

Participants: patients aged 18-70 years between that received a vitamin B12- or D-test.

Primary and secondary outcome measures: the proportion of non-indicated vitamin B12- and D-testing among Dutch clinicians and its variation between hospitals (n=68) over 2015 to 2019.

Results: Between 2015-2019 at least 79.0% of all vitamin B12- and 82% of vitamin D-tests lacked a clear indication. The number of vitamin B12-tests increased by 2% over the examined period, while the number of D-tests increased by 12.2%. The proportion of the unexplained variation in non-indicated vitamin B12- and D-tests that can be ascribed to differences between hospitals remained low. ICCs ranged between 0.072-0.085 and 0.081-0.096 for non-indicated vitamin-B12 and D tests respectively. The included casemix variables patient age, Socioeconomic Status and hospital size only accounted for a small part of the unexplained variation in non-indicated testing. Additionally, a significant correlation was observed in non-indicated vitamin B12- and D-testing among the included hospitals.

Conclusion: Hospital clinicians order vitamin B12- and D-tests without a clear indication on a large scale. Only a small proportion of the unexplained variation could be attributed to differences between hospitals.

Key words: Low-value care, Diagnostic Techniques and Procedures, quality of healthcare

For peer review only

Strengths and limitations:

- This study has examined the ordering and appropriateness of vitamin B12- and D-testing among hospital clinicians and its variation using data over several consecutive years.
- The use of a detailed nationally representative database, allowed us to generate an accurate and reliable overview of the extent of (non-indicated) vitamin B12- and D-testing among exclusively hospital clinicians within the Dutch healthcare system.
- Additionally, the use of registration data derived directly from hospital registration systems, enabled us to accurately distinguish appropriate from inappropriate vitamin testing using diagnose codes rather than the proxy of testing interval.

For peer review only

Introduction

Low-value diagnostic testing is a serious problem in most health care systems [1]. Low-value care is defined as care that offers no net benefit for the patient and which can be associated with harmful outcomes and wasteful spending. [2-4] Within diagnostic testing, studies show that both vitamin B12- and D-tests are frequently ordered within medical practice [5-8]. However, few indications justify the ordering of a vitamin B12- or D-test are described in international guidelines. Especially, among healthy adults, almost no indications for vitamin B12- and D-testing exist. [9, 10] international guidelines for hospital clinicians clearly state that vitamin B12- and D-tests should only be ordered in specific scenarios, such as in patients with Coeliac or Crohn disease, and not in patients with only vague complaints. [9, 11-14]. Assessments indicate that as much as 77% of vitamin B12- and 91% of vitamin D-tests could lack an indication. [7, 15-23]

Studies show that between 8% and 28% of vitamin B12-tests, and between 6% and 91% of vitamin D-tests could be non-indicated. Most of these studies have been conducted among general practitioners (GPs). [17, 19, 21, 23] Only a few studies have clearly specified that their assessment of the volume of non-indicated vitamin testing concerned hospital clinicians alone. Two studies from Italy reported that on average 17% and 6% of vitamin B12- and D-tests were potentially non-indicated. [7, 18] However, these assessments used data from a single hospital and therefore do not provide a representative overview. Furthermore, these studies based their assessment of appropriateness on a proxy such as testing interval, and not directly by examining the diagnose for which the test was requested.

In order to address non-indicated vitamin B12- and D-testing, an accurate assessment of the magnitude and variation of non-indicated testing is needed. We therefore aimed to assess both the volume and proportions of patients aged between 18 and 70 years old that potentially received non-indicated vitamin B12- and D-tests among Dutch hospital clinicians between 2015 and 2019 using clinical registration data. We also examined the hospital variation in non-indicated vitamin B12- and

For peer review only

D-testing in order to identify opportunities for improvement. The proportion of outpatient visits receiving a non-indicated vitamin B12- or D-test among the hospitals included, and the diagnose codes often associated with vitamin B12- and D-testing over 2019 were also explored. In doing so, we aimed to gain more insight into the volume of non-indicated vitamin testing among hospital clinicians and the diagnoses underlying such testing.

Methods

Design and database

We conducted a retrospective cross-sectional study among Dutch hospital clinicians between 2015 and 2019. Data were obtained from the Dutch National Basic Hospital Care Registration (*Landelijke Basisregistratie Ziekenhuiszorg, LBZ)*. [24] The LBZ contains medical, financial and administrative information from all patients undergoing treatment in any Dutch hospital. All vitamin B12- and D-tests ordered by clinicians in Dutch hospitals over the examined time period were extracted; including the associated diagnosis codes, patient age, gender and Socioeconomic Status (SES) and hospital size. After consulting paediatricians and geriatricians, we limited our analysis to patients aged 18 to 70 years old. Both paediatricians and geriatricians indicated that for patients below 18 and above 70, there are many regional screenings protocols which also often vary between hospitals. Patients were assigned to one of three age categories; 18-29, 30-49, 50-70. SES scores were derived from a table containing SES-scores on the level of four-digit postal codes as published by the Dutch Institute for Social Research (SCP) in 2017. [25] Patients were also assigned to one of six SES categories, based on quintiles calculated with the SES information from all Dutch neighbourhoods. Hospital size was operationalized by assigning a hospital to one of three categories (small/medium/large) based on tertiles calculated using the amount of outpatient visits encountered over 2019.

Patient and public involvement

No patients or members of the public were directly involved in the study. Owing to the nature of this study and data privacy constraints, no patients or members of the public were involved in the study design, analysis, interpretation of data, or revision of the manuscript.

Analysis of trends in proportions of non-indicated vitamin B12- and D-testing among hospital clinicians.

For the assessment of the proportion of justified indication versus non-indicated vitamin B12- and D-testing, we used a service lens, as previously described by Chalmers et al. [26] This entails that all vitamin B12- or D-tests ordered were included in our denominator, and all vitamin B12- and D-tests ordered with no indication in our numerator. For our distinction of non-indicated vitamin testing, we followed several steps. First, all recommendations regarding vitamin B12- or D-testing were extracted from the relevant guidelines. Initially, we reviewed the Dutch guidelines of hospital clinicians published by the federation of medical specialists for indications for vitamin B12- and D-testing. [10] We managed to identify little to no recommendations concerning indications regarding the use of vitamin B12- or D-tests. We therefore chose to supplement these with indications derived from Dutch general practitioners guidelines. [13, 14] Second, the ICD10 codes corresponding to the diagnoses which warrant the ordering of a vitamin B12- or D-test were collected from these recommendations. Third, the resulting list of ICD10 codes was reviewed by the involved experts to prevent missing relevant codes or diagnoses. We consulted two expert clinicians (an internal medicine physician and haematologist) in the process of generating the list of indications justifying a vitamin B12- or D-test. Fourth, after completion the list of ICD10 codes, all Clinical Classification Software (CCS) codes associated to these ICD10 codes were extracted from the LBZ database. Subsequently, all Diagnosis-Treatment Combination (DTC) codes associated to the list of relevant CCS codes were extracted. The resulting list was, again, checked for completeness before starting with the assessment of indicated vitamin tests. This process was repeated until all researchers and clinicians agreed on the accuracy of the list of indications justifying vitamin B12- and D-testing.

Supplementary file S1 lists of ICD10 and DTC codes used to determine the appropriateness of the identified vitamin B12- or D-tests. It also contains a description of how we identified and linked the identified ICD10 codes to each of the patients included. In an effort to provide potential handles for the design of interventions, we also examined which diagnosis codes were frequently associated with non-indicated tests.

Assessment of hospital variation in non-indicated vitamin B12- and D-testing.

Hospital variation in non-indicated vitamin B12- and D- testing was assessed using a multilevel logistic regression analysis, with a random effect for hospital. Separate models per year were made to assess whether the variation in non-indicated vitamin B12- and D-testing was robust over time. Generalized variance inflation factors (GVIF) were calculated to test for collinearity among the included variables before multilevel analysis was conducted (supplementary file S2). Models were adjusted for the casemix variables: patient age, gender, Socioeconomic Status (SES), and hospital size. We corrected for patient age, gender and SES, as previous research showed that these affect the amount of care that patients require, receive and have access to. [27-31] We included a proxy for hospital size (e.g. the total number of outpatient visits in each year) while recent evidence shows that larger healthcare providers tend to provide more low-value care. [32] Vitamin tests conducted in patients with a missing SES score or DTC code were excluded from the analysis. Intraclass correlation coefficients (ICCs) were calculated to assess which part of the unexplained variation in non-indicated vitamin B12- and D-testing could be ascribed to differences between the included hospitals, using the method of Snijders and Bosker to assess the error variance. [33]

Correlation in non-indicated vitamin testing over 2019

Additionally, we also examined whether a correlation existed between the proportions of outpatient visits that received a non-indicated vitamin B12- or D-test over 2019 among the hospitals included. Correlations were assessed using the Pearson correlation coefficient for normally distributed

variables and the Spearman correlation coefficient for non-normally distributed variables. Normality was assessed using both density plots and the Shapiro-Wilk test.

For peer review only

Results

Volume vitamin tests among Dutch hospital clinicians.

Table 1 provides a general overview of the population characteristics of the population included in our study. Between 2015 and 2019, the number of vitamin B12- and D-tests ordered by clinicians increased by 2.0% (from 275,032 to 280,522) and 12.2% (from 300,013 to 336,736) respectively. A similar trend was also observed in the proportion of patients that received at least one vitamin B12- or D-test, increasing by 2.5% and 11.3% over the examined period. The amount of vitamin B12- and D-tests ordered among women remained almost twice as high compared to men over the entire period examined. Table 2 provides an overview of the outcomes, supplementary file S3 contains a more detailed breakdown by gender, age- and SES-groups on the patient level. The number of patients with at least one vitamin test increases rapidly with age. The included patients, with at least one vitamin B12 or D-test, showed to be more equally distributed over the SES categories. Only in the highest SES category a slight decrease in the number of patients with a vitamin determination was observed.

Table 1: overview of the study population characteristics

ew only

Non-indicated testing

Between 2015 and 2019 around 78% of the vitamin B12- tests conducted among patients between age 18 and 70, with an registered DTC code, lacked an indication. In case of vitamin D-testing, around 82% of determinations had no clear indication. Although the number of vitamin tests is higher among women, no large differences in proportions of non-indicated testing were observed between genders. In case of both age and SES, the proportion of patients with a non-indicated vitamin B12- or D-test remains relatively constant across all groups over the study period (supplementary file S3). With the proportion of non-indicated testing remaining around 80.0% for vitamin B12 and 83.5% for vitamin D across the different age and SES. Our analysis of diagnose codes that are most often associated with non-indicated vitamin B12- and D-testing revealed that tests are ordered for various reasons. Similar diagnostic codes were associated with both non-indicated vitamin B12 and D tests, including general malaise, fatigue without diagnosis, and ulcerative colitis. Supplementary file S4 contains an overview of the top 20 diagnose codes for both vitamin B12- and D-tests.

Hospital variation in non-indicated vitamin B12- and D-tests among Dutch hospital clinicians

The ICCs of the models uncorrected for casemix remained around 9% (ranging from 8,3% to 9,5%) and 9,5% (ranging from 8,5% to 10,1%) for the vitamin B12- and D-models over time. The ICCs of the casemix corrected vitamin B12- and D-models remained stable around 8% (ranging between 7,2 and 8,5%) and 9% (ranging between 8,1% and 9.6%), respectively, throughout the examined period. Table 3 contains the ICCs of all models. Casemix correction minimally impacted the calculated ICCs in case of both the vitamin B12- and D-models The proportion of outpatient visits receiving a non-indicated vitamin B12- or D-test over 2019 varied widely among the hospitals included, ranging from 0.0% to 27.6% for vitamin B12 and 0.02% to 34.8% for vitamin D (see figure 1 and supplementary file S5).

Figure 1: proportion of all outpatient visits at each of the included hospitals that received a low-value vitamin B12- or D- test over 2019.

For peer review only

Correlation in proportions of non-indicated vitamin B12- and D-testing

Normality testing (and inspection of density plots) revealed that both the proportions of vitamin B12- and D-testing observed among the hospitals are non-normally distributed. The subsequent correlation analysis revealed the presence of a significant positive correlation (Rho: 0.86, $p < 0.001$) between the proportions of non-indicated vitamin B12- and D-testing among the included hospitals. Supplementary file S6 contains the density plots, normality tests results and correlation analysis outcomes for both non-indicated vitamin B12- and D-tests.

For peer review only

1 *Table 2: Overview of vitamin B12 and D tests performed among hospital clinicians.*

2 *Table 3: Hospital variation; overview of Intraclass correlation coefficient across years (ICC) of both the casemix adjusted and unadjusted models for vitamin B12- and D-testing.*

For peer review only

Discussion

Between 2015 and 2019, around 78% and 82% of vitamin B12- and D-tests ordered by Dutch hospital clinicians in patients aged 18-70 lacked a clear indication. The total number of vitamin B12-tests ordered increased by 2.0%, while the total number of vitamin D-tests increased by 12.2%. Although the total number of vitamin determinations increased, the proportion of patients with at least one vitamin test remained relatively constant (around 80% for B12 and 83% for D). Women received approximately twice as many vitamin B12- and D-tests, as well as non-indicated tests, over the examined period compared to men. Our analysis of hospital variation in non-indicated vitamin B12- and D-testing revealed a moderate hospital variation. Furthermore, only a relatively small part of the unexplained variation in non-indicated vitamin B12- and D- testing could be ascribed to differences between the hospitals included. Suggesting that the problem of non-indicated vitamin testing is present among all hospitals. Correlation analysis over 2019 revealed a fairly strong positive (Rho: 0.86) correlation between the rates of non-indicated vitamin B12- and D-testing among the hospitals included.

Comparison to existing literature.

The proportion of non-indicated vitamin B12- and D-testing among clinicians found in our study (78% and 82% respectively) are substantially larger than the proportions reported by other studies. For example, two studies from Italy conducted among hospital clinicians report that on average 17% and 6% of vitamin B12- and D-tests could potentially be non-indicated. [7, 18] Which is substantially lower than the proportions observed in our study. Our findings are more in line with assessments that are not limited to hospital clinicians. Hence, studies conducted among general practitioners indicate that between 8% and 28% of vitamin B12- [7, 15, 16, 18] and between 7% and 91% of vitamin D-tests could be considered non-indicated. [7, 15, 19, 21, 23] Among these studies, only Naugler et al. and Gonalez-Chicas reported proportions of non-indicated vitamin D testing which is similar to ours. Naugler et al., found that following an intervention the prevalence of non-indicated

vitamin D-testing decreased by 91.4%. [19] While Gonzalez-Chica reported 76,5% of vitamin D-tests to be non-indicated after introduction of new Medicare criteria for rebates. Besides population differences (e.g. GPs versus hospital clinicians), varying definitions of non-indicated testing and methods used could also account for the large differences in assessment outcomes. For example, both our study and that of Naugler et al. utilized diagnosis codes to distinguish the appropriateness of vitamin testing. [19] Most assessments of the appropriateness of vitamin testing performed to date used testing intervals or laboratory results to discern non-indicated testing, as did Gonzalez-Chica. [23] This methodological difference might explain the large differences in assessment outcomes. Furthermore, the studies that did not limit their assessment to vitamin testing among GPs, were often conducted within a single hospital. [7, 18] Moreover, most studies that assessed vitamin B12- or D-testing do not specify which type of physicians (GP/hospital clinicians) or vitamin B12- and D-tests were included, making comparison to our study challenging.

The absence of clear recommendations regarding the *"appropriate"* use of vitamin testing in clinician guidelines, does not aid clinicians to appropriately order vitamin testing. The current guidelines for hospital clinicians offer little direction on the appropriate use of vitamin B12 or D tests, leaving clinicians with little guidance when deciding whether to order such tests. These difficulties are magnified by the large knowledge gaps regarding the exact roles of vitamin B12 and D within the human body and its metabolism. [34, 35] The combination of these factors may provide an explanation for the high proportion of non-indicated testing. Hence, due to the lack of a clear understanding of the roles of vitamin B12 and D within the body and the fear of missing a diagnosis, clinicians may engage in defensive behaviour resulting in the ordering of vitamin tests

Strengths and limitations.

A strength of this study is its novelty in examining the ordering and appropriateness of vitamin B12- and D-testing among hospital clinicians and its variation over several consecutive years. A second strength is that we used a detailed nationally representative database. This allowed us to generate

an accurate and reliable overview of the extent of vitamin B12- and D-testing among solely hospital clinicians. Furthermore, it also enabled us to accurately distinguish appropriate from inappropriate vitamin testing using diagnose codes rather than the proxy of testing interval.

However, our study is also prone to limitations. First, we based our distinction of appropriateness mainly on expert opinion and indications derived from the Dutch GP guidelines, as no universal guideline regarding vitamin testing among hospital clinicians exists. We therefore might have misclassified some of the tests from clinicians as being non-indicated. However, we tried to minimize the risk of misclassification by closely collaborating with the involved experts with respect to the creation of the list of indications. Additionally, some differences exist between international and Dutch guidelines regarding indications for vitamin B12- and D-testing. For example, guidelines from the US indicate that vitamin B12-testing is considered indicated in patients with cognitive impairments or dementia. [36] However, these are not considered as an indication according to the nationwide guidelines published by the Dutch federation of medical specialists. [10] Potentially, such recommendations could exist in local protocols of Dutch hospitals, which unfortunately are not publicly available. This makes it difficult to compare international assessment outcomes to our study, while subtle differences between guidelines might cause large differences in the used criteria of appropriateness (and subsequently the reported outcomes).

Second, the use of DTC codes enabled us to accurately distinguish the appropriateness of vitamin tests, but also has a drawback. DTC diagnosis codes are generally less specific compared to the ICD10 codes described in the used guidelines. We therefore might have misclassified some vitamin tests as being appropriate. Furthermore, although DTC diagnosis codes provide a lot of insight into the diagnosis associated with practices, their registration is prone to misregistration. [37] Clinicians have a vast amount of (often similar) DTC-codes to choose from when registering a diagnosis code, thereby adding another layer of complexity to the correct registration of diagnoses. Furthermore, DTC codes are updated as the patient passes through the healthcare system. The registered DTC

For peer review only - http://bmjopen.bmj.com/site/about/guidelines.xhtml

code therefore does not necessarily represent the initial reason (or diagnosis) for which the vitamin determination was ordered, but rather reflects the final diagnosis.

Third, some Dutch hospitals outsource their tests to external commercial laboratories, which are not registered in the LBZ. Our estimate of vitamin testing by clinicians therefore is not complete. However, according to the registration of the national statistical office, Statistics Netherlands (CBS), 82 hospitals were active in 2019. Since we were able to include data from the majority of the hospitals in the Netherlands in our study (68/82, e.g. 83% of all hospitals), we do not expect to have missed much in our analysis. [38]

Implications for research and practice

Our assessment reveals that a large proportion of vitamin B12- and D- tests is ordered without a clear indication justifying their use. The total volumes of vitamin B12- and D-tests have increased over the years and show no inclination of declining. Based on publicly available fares, we estimates that roughly 3.8 million euro has been spent on non-indicated vitamin B12- and D-tests in 2019 by Dutch hospital clinicians alone. [39] This estimate of the potential savings, however, is very rough, while it only accounts for the cost price of a vitamin B12- or D-determination. The observed incidence rates of non-indicated vitamin B12- and D-testing, however, suggest that there is ample opportunity to reduce vitamin testing among Dutch clinicians. Especially since non-indicated vitamin B12- and D-tests are often ordered for similar diagnoses, and a positive correlation exists between the proportions of non-indicated testing within the same hospitals. We know that there are effective interventions to reduce inappropriate vitamin testing among GPs. A study among Dutch GPs showed that providing both education and feedback successfully reduced the amount of vitamin tests ordered with 20-25%. [40] Similar interventions might therefore also be effective among hospital clinicians to reduce (non-indicated) vitamin testing. Future research could focus on further examination of patient and physicians' characteristics associated with non-indicated vitamin B12- and D-testing. Unfortunately, information regarding the requesting physician (age, sex etc.) of non-

For peer review only - http://bmjopen.bmj.com/site/about/guidelines.xhtml

indicated tests was not available to us in our study. Insight into physician characteristics associated with non-indicated testing could aid in the design of interventions aiming to address the problem of non-indicated vitamin testing.

Conclusion

Our research shows that the number of vitamin B12-tests slightly increased over the examined time period, while the number of vitamin D tests substantially increased among hospital clinicians. Throughout the examined period the proportion of B12- and D-tests without clear indication remained high, and are substantially higher compared to similar (international) assessments. The observed difference in assessment outcome can potentially be explained by differences in methods and definitions used to identify and define non-indicated vitamin B12- and D-tests (e.g. the use of associated diagnose codes instead of test results or proxy's such as testing interval). We also observed the presence of moderate hospital variation, but that this variation could not be explained by the included patient and hospital characteristics age, sex, SES and hospital size. Hospitals hardly differ in the task they have to undertake: bring down the number of non-indicated B12- and D-tests.

For peer review only

Additional information requested at submission

Financial disclosure statement

This work was supported by ZonMw, the Dutch organization for Health Research and Development (grant number 839205002). The funder had no role in the design and conduct of the study**.**

Conflict of interest interests

None to declare.

Acknowledgements:

The authors would like to thank Prof. dr. P. Lips, Professor Emeritus, APH - Aging & Later Life, Professor Emeritus of Internal medicine, Visiting Fellow Internal medicine from the Amsterdam UMC, dr. L. de Boer and dr. A. Dittrich for their critical review and contributions to the paper.

Related manuscripts

The authors indicate that they do not have a related or duplicate manuscript under consideration (or accepted) for publication elsewhere.

For peer review only

130 **Author contributions:**

131 *Conceptualization:* Müskens, Kool, van Dulmen, Westert.

132 *Data Curation:* Zaal

133 *Formal Analysis*: Müskens, Atsma

134 *Funding Acquisition*: Kool, van Dulmen

135 *Investigation:* Müskens, Kool, van Dulmen, Muller

136 *Methodology*: Müskens, Atsma.

137 *Project Administration*: Müskens

138 *Resources*: Zaal

139 *Software*: Müskens

140 *Supervision*: Kool, van Dulmen, Westert

141 *Validation*: Müskens, Kool, van Dulmen

142 *Visualization*: Müskens

143 *Writing – original draft preparation*: Müskens, Kool, van Dulmen

144 *Writing – Review & Editing*: Müskens, Kool, van Dulmen, Zaal, Muller, Atsma, Westert

For peer review only

References

1. Müskens JLJM, Kool RB, van Dulmen SA, Westert GP. Overuse of diagnostic testing in healthcare: a systematic review. BMJ Qual Saf. 2022;31(1):54-63.
2. Brownlee S, Chalkidou K, Doust J, Elshaug AG, Glasziou P, Heath I, et al. Evidence for overuse of medical services around the world. Lancet. 2017;390(10090):156-68.
3. Colla CH, Mainor AJ, Hargreaves C, Sequist T, Morden N. Interventions Aimed at Reducing Use of Low-Value Health Services: A Systematic Review. Med Care Res Rev. 2017;74(5):507-50.
4. Scott IA, Duckett SJ. In search of professional consensus in defining and reducing low-value care. Med J Aust. 2015;203(4):179-81.
5. Essig S, Merlo C, Reich O, Trottmann M. Potentially inappropriate testing for vitamin D deficiency: a cross-sectional study in Switzerland. BMC Health Services Research. 2020;20(1):1097.
6. Crowe FL, Jolly K, MacArthur C, Manaseki-Holland S, Gittoes N, Hewison M, et al. Trends in the incidence of testing for vitamin D deficiency in primary care in the UK: a retrospective analysis of The Health Improvement Network (THIN), 2005–2015. BMJ Open. 2019;9(6):e028355.
7. Lanzoni M, Fornili M, Felicetta I, Maiavacca R, Biganzoli E, Castaldi S. Three-year analysis of repeated laboratory tests for the markers total cholesterol, ferritin, vitamin D, vitamin B(12) , and folate, in a large research and teaching hospital in Italy. J Eval Clin Pract. 2017;23(3):654-61.
8. O'Sullivan JW, Stevens S, Hobbs FDR, Salisbury C, Little P, Goldacre B, et al. Temporal trends in use of tests in UK primary care, 2000-15: retrospective analysis of 250 million tests. Bmj. 2018;363:k4666.
9. American Society for Clinical Pathology. Thirty Five Things Physicians and Patients Should Question 2021 [Available from: https://www.choosingwisely.org/clinician-lists/ascp-do-not-order-red-blood-cell-folate-levels/].
10. Richtlijnen database [Internet]. Federatie Medisch Specialisten,. 2022 [cited 12-12-2022]. Available from: https://richtlijnendatabase.nl/.
11. Dutch College of Clinical Chemistry and Laboratory Medicine & College of medical immunologists. Verstandige keuzes bij het aanvragen en interpreteren van laboratoriumdiagnostiek. 2015.
12. Canadian Association of Pathologists. Choosingwisely Canada Pathology Recommendations,. 2021.
13. Nederlands Huisartsen Genootschap. Laboratoriumdiagnostiek Vitamine B12-deficiëntie (LESA): NHG; 2018 [Available from: https://www.nhg.org/themas/publicaties/laboratoriumdiagnostiek-vitamine-b12-volledige-tekst.
14. Nederlands Huisartsen Genootschap. Vitamine D-deficiëntie (LESA Laboratoriumdiagnostiek): NHG; 2018 [Available from: https://www.nhg.org/themas/publicaties/vitamine-d-deficientie-samenvatting?tmp-no-mobile=1].
15. Chami N, Simons JE, Sweetman A, Don-Wauchope AC. Rates of inappropriate laboratory test utilization in Ontario. Clin Biochem. 2017;50(15):822-7.
16. Morgen EK, Naugler C. Inappropriate repeats of six common tests in a Canadian city: a population cohort study within a laboratory informatics framework. Am J Clin Pathol. 2015;144(5):704-12.
17. Gill P, Guo M, Lau CK, Naugler C. Vitamin B12 test volume data before and after the implementation of an educational province-wide intervention to reduce redundant testing in Alberta. Data Brief. 2019;27:104785.

For peer review only

18. Tamburrano A, Vallone D, Carrozza C, Urbani A, Sanguinetti M, Nicolotti N, et al. Evaluation and cost estimation of laboratory test overuse in 43 commonly ordered parameters through a Computerized Clinical Decision Support System (CCDSS) in a large university hospital. PLoS One. 2020;15(8):e0237159.
19. Naugler C, Hemmelgarn B, Quan H, Clement F, Sajobi T, Thomas R, et al. Implementation of an intervention to reduce population-based screening for vitamin D deficiency: a cross-sectional study. CMAJ Open. 2017;5(1):E36-e9.
20. Colla CH, Morden NE, Sequist TD, Schpero WL, Rosenthal MB. Choosing wisely: prevalence and correlates of low-value health care services in the United States. J Gen Intern Med. 2015;30(2):221-8.
21. Mafi JN, Reid RO, Baseman LH, Hickey S, Totten M, Agniel D, et al. Trends in Low-Value Health Service Use and Spending in the US Medicare Fee-for-Service Program, 2014-2018. JAMA Network Open. 2021;4(2):e2037328-e.
22. Zorginstituut Nederland. Screeningsrapport Endocriene Ziekten; voedings- en stofwisselingstoornissen,. Zorginstituut Nederland; 2018.
23. Gonzalez-Chica D, Stocks N. Changes to the frequency and appropriateness of vitamin D testing after the introduction of new Medicare criteria for rebates in Australian general practice: evidence from 1.5 million patients in the NPS MedicineInsight database. BMJ Open. 2019;9(3):e024797.
24. Dutch Hospital Data. Landelijke Basisregistratie Zorg (LBZ). In: Data DH, editor.
25. The Netherlands Institute for Social Research. Statusscores 2017. Netherlands: The Netherlands Institute for Social Research (SCP); 2017.
26. Chalmers K, Pearson SA, Elshaug AG. Quantifying low-value care: a patient-centric versus service-centric lens. BMJ Qual Saf. 2017;26(10):855-8.
27. McMaughan DJ, Oloruntoba O, Smith ML. Socioeconomic Status and Access to Healthcare: Interrelated Drivers for Healthy Aging. Front Public Health. 2020;8:231.
28. Arpey NC, Gaglioti AH, Rosenbaum ME. How Socioeconomic Status Affects Patient Perceptions of Health Care: A Qualitative Study. J Prim Care Community Health. 2017;8(3):169-75.
29. Asch SM, Kerr EA, Keesey J, Adams JL, Setodji CM, Malik S, McGlynn EA. Who is at greatest risk for receiving poor-quality health care? N Engl J Med. 2006;354(11):1147-56.
30. David JL, Kaplan HB. Gender, social roles and health care utilization. Applied Behavioral Science Review. 1995;3(1):39-64.
31. Bertakis KD, Azari R, Helms LJ, Callahan EJ, Robbins JA. Gender differences in the utilization of health care services. J Fam Pract. 2000;49(2):147-52.
32. Segal JB, Sen AP, Glanzberg-Krainin E, Hutfless S. Factors Associated With Overuse of Health Care Within US Health Systems: A Cross-sectional Analysis of Medicare Beneficiaries From 2016 to 2018. JAMA Health Forum. 2022;3(1):e214543-e.
33. Snijders TAB, Bosker RJ. Multilevel Analysis: An Introduction to Basic and Advanced Multilevel Modeling: SAGE Publications; 2011.
34. Holick MF. Vitamin D deficiency. N Engl J Med. 2007;357(3):266-81.
35. Green R, Miller JW. Chapter Fifteen - Vitamin B12 deficiency. In: Litwack G, editor. Vitamins and Hormones. 119: Academic Press; 2022. p. 405-39.
36. Knopman DS, DeKosky ST, Cummings JL, Chui H, Corey-Bloom J, Relkin N, et al. Practice parameter: diagnosis of dementia (an evidence-based review). Report of the Quality Standards Subcommittee of the American Academy of Neurology. Neurology. 2001;56(9):1143-53.
37. Marcotte LM, Schuttner L, Liao JM. Measuring low-value care: learning from the US experience measuring quality. BMJ Qual Saf. 2020;29(2):154-6.
38. Statistics Netherlands (CBS). [Zorginstellingen; kerngetallen]. 2019.
39. Diagnostiek voor u. Fares 2022 - 2023 2023 [Available from: https://diagnostiekvooru.nl/tarieven].

For peer review only

246 40. Vugt SV, de Schepper E, van Delft S, Zuithoff N, de Wit N, Bindels P. Effectiveness of professional and patient-oriented strategies in reducing vitamin D and B12 test ordering in primary care: a cluster randomised intervention study. BJGP Open. 2021;5(6).

For peer review only

250 *Table 3: overview of the study population characteristics*

251

General info regarding the used population between 2015 - 2019	Number or proportion	Min	Max
Total number of unique patients	9,214,425	N.A.	N.A.
Gender (% female)	64.50%	N.A.	N.A.
Average number of unique patients among the hospitals (+SD)	133,542 (± 169,110)	37	1,244,526
Average age of the patients included (+SD)	47.91 (± 14.60)	18	70
Average SES category of the patients included (+SD)	2.86 (± 1.43)	1	6
Average no. of outpatient visits among the hospitals included	1,721,337 (± 819,148)	470,117	4,659,172

For peer review only

Table 4: Overview of vitamin B12 and D tests performed among hospital clinicians.

Year	2015	2016	2017	2018	2019
**No. of hospitals included**	63	64	66	69	68
**Total no. B12-tests**	275,032	265,546	279,524	279,957	280,522
**Total no. B12-tests in hospital with a registered DTC code**	118,126	163,244	188,063	208,012	213,308
% of B12-test with a registered DTC without clear indication	79.0%	78.0%	78.2%	78.3%	78.1%
**Total no. of patients with (at least) one vitamin B12-test**	233,541	226,999	239,033	240.063	239.351
**Total no. of patients with (at least) one vitamin B12-test with a registered DTC code**	103,540	141,507	162,900	178,933	183,204
**Total no. of patients with (at least) one non-indicated vitamin B12-test (with a registered DTC code)**	83,549	111,986	128,872	141,914	145,039
% patients that received at least one B12-test that was considered non-indicated	80.7%	79.1%	79.1%	79.3%	79.2%
**No. of hospitals included**	62	61	63	65	65
**Total no. D tests**	300,013	300,427	322,703	323,074	336,736
**Total no. D tests in hospital with a registered DTC code**	121,892	179,271	208,361	230,620	242,040
% of D-test with a registered DTC without clear indication	82.5%	82.9%	82.7%	82.5%	82.0%
**Total no. of patients with (at least) one vitamin D-test**	244,834	247,186	264,811	265,362	272,380
**Total no. of patients with (at least) one vitamin D-test with a registered DTC code**	101,932	148,660	172,342	189,423	196,452
**Total no. of patients with (at least) one non-indicated vitamin D-test (with a registered DTC code)**	84,414	124,162	143,408	157,431	162,345
% patients that received at least one B12-test that was considered non-indicated	82.8%	83.5%	83.2%	83.1%	82.6%

For peer review only

254 *Table 3: Hospital variation; overview of Intraclass correlation coefficient across years (ICC) of both the casemix adjusted and unadjusted models for vitamin B12- and D-testing.*

	**2015**		**2016**		**2017**		**2018**		**2019**	
	Unadjusted	*Adjusted*	*Unadjusted*	*Adjusted*	*Unadjusted*	*Adjusted*	*Unadjusted*	*Adjusted*	*Unadjusted*	*Adjusted*
**Vitamin-B12**	0.083	0.072	0.094	0.085	0.088	0.081	0.089	0.079	0.095	0.082
**Vitamin-D**	0.101	0.095	0.096	0.083	0.085	0.081	0.095	0.090	0.099	0.095

255

For peer review only

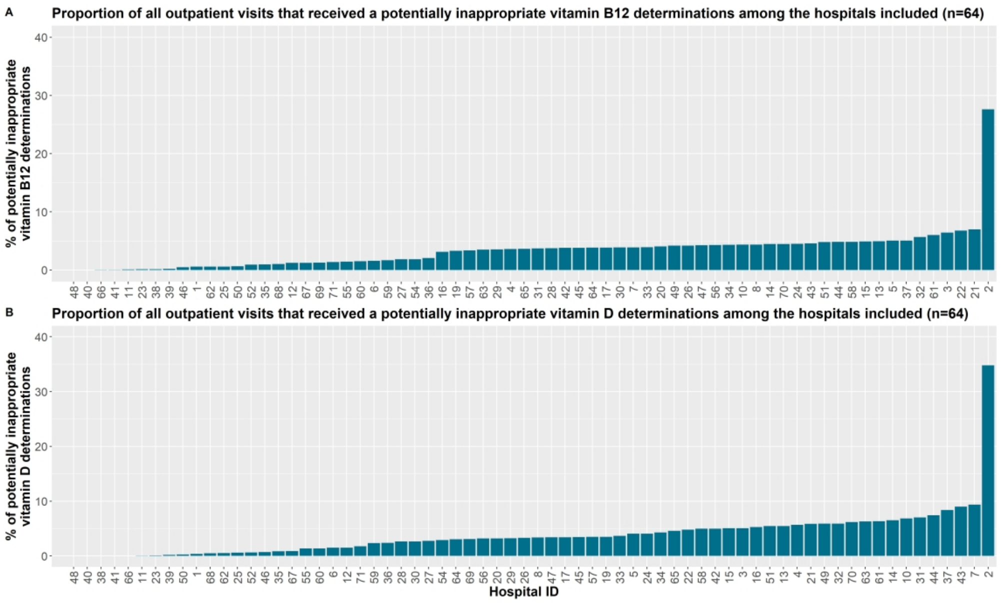

Figure 1: proportion of all outpatient visits at each of the included hospitals that received a low-value vitamin B12- or D- test over 2019.

423x254mm (300 x 300 DPI)

Supplementary file S1: Included International classification of disease (ICD10) codes and subsequent diagnose-treatment combination (DTC) codes.

The table below contains the codes which were used to distinguish indicated from non-indicated vitamin B12- and D-tests. In case of vitamin D-testing, tests were considered to be appropriate in patients older than 50 with a fracture. We therefore have also provided a table of the fractures that were included.

**Vitamin B12**			
**ICD-10 code**	**Description**	**DTC code**	**DTC-Diagnosis description**
D51	**Vitamin B_{12} deficiency anaemia**<br>*Excl.: Vitamin B_{12} deficiency (E53.8)*	- 313 – 701<br>- 313 – 702<br>- 313 – 709<br>- 316 – 6001<br>- 316 – 6003<br>- 335 – 232	- Iron-deficiency anemia (not specified)<br>- Pernicious anemia<br>- Other erytrocital deviations (not specified)<br>- Anemia, Iron-deficiency<br>- Anemia, remaining<br>- Anemia
D52	**Folate deficiency anaemia**		
D53	**Other nutritional anaemias**<br>*Incl.: megaloblastic anaemia unresponsive to vitamin B_{12} or folate therapy*		
E53.8	**Deficiency of other specified B group vitamins**<br>*Deficiency:*<br>• *Biotin*<br>• *Cyanocobalamin*<br>• *Folate*<br>• *Folic acid*<br>• *Panthothenic acid*<br>• *Vitamin B12*	- 318 – 207<br>- 330 – 301	- (mal)nutrition<br>- Vitamin-deficiencies
E56.8	**Deficiency of other vitamins**		
E56.9	**Vitamin deficiency, unspecified**		
G63.4	**Polyneuropathy in nutritional deficiency**	- 327 – 413<br>- 330 – 312<br>- 330 – 812<br>- 335 – 251	- Pheripheral nervedamage, nerve disorder<br>- Neurological complication systemic disease<br>- Polyneuropathy, other<br>- Diseases of the nerve system and senses
R20.2	**Paraesthesia of skin**<br>*Excl.: acroparaesthesia (I73.8)*		
R27.0	**Ataxia, unspecified**		
K29.4	**Chronic atrophic gastritis**	- 313 – 916<br>- 318 – 401	- Erosive gastritis and duodenitis<br>- Gastritis, miscellaneous
K50	**Crohn disease [regional enteritis]**<br>*Incl.: granulomatous enteritis*<br>*Excl.: ulcerative colitis (K51.-)*	- 303 – 326<br>- 313 – 922<br>- 318 – 601<br>- 316 - 3314	- Crohn disease (enteritis regionalis)<br>- Crohn disease<br>- Morbus Crohn<br>- Inflammatory bowldisease (colitis ulcerosa/Morbus crohn)
K52	**Other noninfective gastroenteritis and colitis**	- 313 – 932<br>- 313 – 938	- Chronic diarrhoea without infection<br>- Radiation enteritis
K90	**Intestinal malabsorption**<br>*Excl."following gastrointestinal surgery (K91.2)*	- 318 - 409	- Coeliac disease
Z98.0	**Internal bypass and anastomosis status**	- 303 – 341<br>- 303 – 342	- Morbid obesity BMI < 45<br>- Morbid obesity BMI > 45

For peer review only

Vitamin D			
**ICD-10 code**	**Description**	**DTC code**	**DTC-Diagnosis description**
E20<br>E21	**Hypoparathyroidism**<br>**Hyperparathyroidism and other disorders of parathyroid gland**	- 307 – E00<br>- 307 – E11<br>- 313 – 231<br>- 313 – 232<br>- 316 – 7110<br>- 316 – 7199<br>- 362 – 304	- Emergency consult Endocrinology<br>- Endocrinology<br>- Hyperparathyroidism<br>- Hypoparathyroidism<br>- Small body length/deviant growth curve<br>- Endocrinology, not specified<br>- Parathyroid glands
E83.5	**Disorders of calcium metabolism**	- 313 – 239<br>- 313 – 299<br>- 316 - 7503<br>- 330 - 399	- Calcium metabolic disease, unspecified<br>- Endocrine and metabolic diseases, unspecified<br>- Metabolic disease<br>- Deficiencies, metabolic, nutritional, unspecified
E84.9	**Cystic fibrosis, unspecified**	- 316 – 3205<br>- 322 - 1403	- Cystic fibrosis<br>- Cystic fibrosis
E55	**Vitamin D deficiency**<br>*Excl.: adult osteomalacia (M83.-), osteoporosis (M80-M81), sequelae of rickets (E64.3)*	- 305 – 1062<br>- 335 – 221<br>- 318 – 207<br>- 335 – 224	- Rachitis and/or vitamin D resistance osteomalacia<br>- Endocrine and nutrition disorders<br>- (mal)nutrition<br>- Calory/protein malnutrition
M83	**Adult osteomalacia**<br>*Excl.: osteomalacia:*<br>• *infantile and juvenile (E55.0)*<br>• *vitamin-D-resistant (E83.3)*<br>*Renal osteodystrophy (N25.0)*<br>*Rickets (active) (E55.0)*<br>*Rickets (active)*<br>• *sequelae (E64.3)*<br>*vitamin-D-resistant (E83.3)*		
E83.3	**Disorders of phosphorus metabolism and phosphates**<br>Acid phosphatase deficiency<br>Familial hypophosphataemia<br>Hypophosphatasia<br>Vitamin-D-resistant:<br>• Osteomalacia<br>• rickets<br>*Excl.: adult osteomalacia (M83.-), osteoporosis (M80-M81)*	- 316 – 3328<br>- 316 – 7503	- Food related problems/disorders<br>- Metabolic disease
K50	**Crohn disease [regional enteritis]**<br>*Incl.: granulomatous enteritis*<br>*Excl.: ulcerative colitis (K51.-)*	- 303 – 326<br>- 313 – 922<br>- 318 – 601	- Crohn disease (enteritis regionalis)<br>- Crohn disease<br>- Morbus Crohn
K90	**Intestinal malabsorption**<br>*Excl."following gastrointestinal surgery (K91.2)*	- 313 – 920<br>- 318 – 409	- Coeliac disease / malabsorption<br>- Coeliac disease
M80	**Osteoporosis with pathological fracture**<br>*Incl.: osteoporotic vertebral collapse and wedging*<br>*Excl.: collapsed vertebra NOS (M48.5), pathological fracture NOS (M84.4), wedging of vertebra NOS (M48.5)*	- 305 – 1395<br>- 316 – 5105	- Osteoperotic collapse<br>- Pathological fractures

For peer review only

M81	**Osteoporosis without pathological fracture**<br>*Excl.: osteoporosis with pathological fracture (M80.-)*	- 313 – 233	- Osteoperosis, osteomalacia
M82	**Osteoporosis in diseases classified elsewhere**		
N25.0	**Renal osteodystrophy**<br>*Azotaemic osteodystrophy*<br>*Phosphate-losing tubular disorders*<br>*Renal:*<br>• *rickets*<br>• *short stature*	- 313 – 399<br>- 313 – 325	- Renal diseases, miscellaneous<br>- Chronic renal disease (eGFR <30 ml/min)

Additional DTC codes added after checking the resulting indications:

D	0316	3304	Celiac disease	6509
D	0316	4006	Chronic renal failure	1245
D	0318	753	Chronic pancreatitis	3415
B12	0316	3304	Celiac disease	6233
B12	0313	772	Polycythemia vera, essential trombocytosis	5434
B12	0313	763	Myelodysplastic syndrome no specified	4843
B12	0313	920	Celiac disease/malabsorption	4206
B12	0313	773	Chronic myelogenous leukemia (CMMoL)	312

For peer review only

Bone fractures above 50 included in our analysis, that justify the ordering of a vitamin D-test.

DTC specialism code	DTC diagnose code	Fracture description.
0305	3011	Radius head
0305	3014	Carpus
0305	3029	Metatarsal bones
0303	207	Humerus proximal and shaft
0305	3024	Tibia (with or without fibula, excluding ankle)
0303	210	Radius head
0304	451	Fracture of distal radius surgical
0303	219	Femur, remaining
0303	221	Tibial plateau
0303	222	Tibia (± fibula, excluding ankle)
0305	3023	Tibial plateau
0303	217	Pelvis / sacrum
0303	214	Metacarpal bones
0305	3008	Humerus proximal and shaft
0303	224	Ankle
0305	3026	Calcaneus
0305	3030	Phalanges of the feet
0308	2301	Trauma vertebral column: conservative treatment
0303	241	Talus
0305	3020	Femur, remaining
0303	237	Tarsus
0303	215	Phalanges of the hand
0305	3027	Talus
0304	420	Fracture / luxation carpalia conservative
0305	3017	Pelvis
0305	3005	Vertebral column with paraplegia
0303	220	Patella
0303	238	Metatarsal bones
0305	3004	Vertebral column
0305	3009	Distal humerus/(epi)condyl(len)
0305	3015	Metacarpal bones
0305	3022	Fibula
0305	3025	Ankle
0303	239	Phalanges of the feet
0303	209	Olecranon
0303	213	Carpus
0305	3013	Wrist
0308	2311	Trauma vertebral column: Surgical intervention including instrumentation c.q. spinal fusion
0304	450	Distal radius facture conservative
0304	8418	Traumatic Vertebral compression fracture
0303	211	Forearm, no further specification
0305	3021	Patella
0304	421	Fracture / luxation carpalia operative
0303	212	Wrist
0303	208	Distal humerus / (epi)condyl(en)
0303	203	Vertebral column
0303	204	Vertebral column with spinal lesion
0308	2305	Treatment of trauma to the vertebral column with external fixation or traction
0305	3010	Olecranon

For peer review only

0305	3028	Tarsus
0303	236	Calcaneus
0305	3012	Forearm
0305	3016	Phalanges of the hand
0303	218	Fracture of neck of femur (disorder)
0305	3019	Femur proximaal (+ collum)

For peer review only

Supplementary file S2: overview calculated generalized variance inflation factors (GVIF).

Generalized Variation Inflation Factors (GVIF) calculated for the models made using DHD data.

Year	Vitamin test	Variable	GVIF	GVIF^(1/(2*DF))
2015	Vitamin B12	*Gender*	1.038412	1.019025
		Age category	1.039533	1.009740
		Socio-economic status category	1.016783	1.002083
		Hospital size	1.016361	1.004065
	Vitamin D	*Gender*	1.013052	1.006505
		Age category	1.015735	1.003911
		Socio-economic status category	1.028674	1.003540
		Hospital size	1.028064	1.006943
2016	Vitamin B12	*Gender*	1.036084	1.017882
		Age category	1.037416	1.009225
		Socio-economic status category	1.013498	1.001677
		Hospital size	1.013126	1.003266
	Vitamin D	*Gender*	1.011964	1.005964
		Age category	1.013244	1.003295
		Socio-economic status category	1.019246	1.002386
		Hospital size	1.019576	1.004858
2017	Vitamin B12	*Gender*	1.034843	1.017272
		Age category	1.036149	1.008917
		Socio-economic status category	1.017334	1.002150
		Hospital size	1.017386	1.004319
	Vitamin D	*Gender*	1.013020	1.006489
		Age category	1.014772	1.003673
		Socio-economic status category	1.022610	1.002799
		Hospital size	1.022912	1.005679
2018	Vitamin B12	*Gender*	1.037842	1.018745
		Age category	1.039502	1.009733
		Socio-economic status category	1.024565	1.003038
		Hospital size	1.024302	1.006021
	Vitamin D	*Gender*	1.015657	1.007798
		Age category	1.017710	1.004398
		Socio-economic status category	1.029201	1.003604
		Hospital size	1.029685	1.007340
2019	Vitamin B12	*Gender*	1.035305	1.017499
		Age category	1.036884	1.009096
		Socio-economic status category	1.034798	1.004285
		Hospital size	1.034633	1.008548
	Vitamin D	*Gender*	1.014550	1.007249
		Age category	1.016820	1.004179
		Socio-economic status category	1.039715	1.004880
		Hospital size	1.039941	1.009839

For peer review only

Supplementary file S3: Extended table 1; distribution of vitamin B12- and D-testing across the sex, age and SES categories.

Table 1: extended table 1 for vitamin B12.

	**1. Vitamin B12**															
		**2015**			**2016**			**2017**			**2018**			**2019**		
	No. of hospitals included	63			64			66			69			68		
	Total no. of unique patients with (at least) one vitamin B12 test	233,541			226,999			239,033			240,063			239,351		
	Total no. of unique patients with (at least) one vitamin B12 test with an associated DBC code	103,540			141,507			162,900			178,933			183,204		
	Total no. of unique patients with (at least) one non-indicated vitamin B12 test	83,549			111,986			128,872			141,914			145,039		
		**Total**	**Without (clear) indication**	**% without (clear indication**	**Total**	**Without (clear) indication**	**% without (clear indication**	**Total**	**Without (clear) indication**	**% without (clear indication**	**Total**	**Without (clear) indication**	**% without (clear indication**	**Total**	**Without (clear) indication**	**% without (clear indication**
**Male**	**No. of male patients receiving at least one vitamin-B12 test**	38,308	31,731	82.8	52,360	42,758	81.7	60,511	49,222	81.3	66,724	54,567	81.8	67,869	55,148	81.3
Age cat.	**18-29**	3,152	2,461	78.1	4,473	3,421	76.5	5,262	4,094	77.8	5,921	4,579	77.3	6,468	4,886	75.5
	**30-49**	9,785	7,967	81.4	13,346	10,598	79.4	15,247	12,119	79.5	16,397	13,175	80.4	16,864	13,572	80.5
	**50-69**	25,371	21,303	84.0	34,541	28,739	83.2	40,002	33,009	82.5	44,406	36,813	82.9	44,537	36,690	82.4
Neighbou	***Lowest socio-economic status****	8,070	6,693	82.9	11,346	9,271	81.7	13,512	11,082	82.0	14,632	12,008	82.1	15,099	12,346	81.8
**SES cat.**	***Socio-economic status below average***	8,484	7,022	82.8	11,347	9,236	81.4	13,601	11,078	81.4	14,686	12,001	81.7	14,962	12,116	81.0
	Average socio-economic status	7,790	6,472	83.1	10,372	8,472	81.7	11,669	9,447	81.0	13,296	10,908	82.0	13,086	10,593	80.9
	Socio-economic status above average	8,067	6,698	83.0	10,481	8,576	81.8	11,738	9,480	80.8	12,914	10,527	81.5	13,150	10,687	81.3
	Highest socio-economic status	5,639	4,625	82.0	8,426	6,860	81.4	9,564	7,766	81.2	10,666	8,665	81.2	10,985	8,915	81.2
	Missing	258	221	85.7	388	343	88.4	427	369	86.4	530	458	86.4	587	491	83.6
**Female**	**No. of female patients receiving at least one vitamin-B12 test**	65,232	51,818	79.4	89,147	69,228	77.7	102,389	79,650	77.8	112,209	87,347	77.8	115,335	89,891	77.9
Age cat.	**18-29**	9,138	7,155	78.3	12,781	9,751	76.3	14,863	11,415	76.8	16,799	13,011	77.5	17,371	13,487	77.6
	**30-49**	24,905	18,991	76.3	34,030	25,313	74.4	38,672	28,996	75.0	42,186	31,649	75.0	43,276	32,826	75.9
	**50-69**	31,189	25,672	82.3	42,336	34,164	80.7	48,854	39,239	80.3	53,224	42,687	80.2	54,688	43,578	79.7
Neighbou	***Lowest socio-economic status****	14,351	11,223	78.2	19,935	15,267	76.6	23,709	18,370	77.5	25,526	19,832	77.7	26,005	20,190	77.6
SES cat.	***Socio-economic status below average***	14,520	11,513	79.3	19,865	15,328	77.2	23,072	17,820	77.2	25,157	19,555	77.7	25,860	20,112	77.8
	Average socio-economic status	13,035	10,453	80.2	17,656	13,737	77.8	19,669	15,213	77.3	21,936	17,000	77.5	22,334	17,366	77.8
	Socio-economic status above average	13,122	10,459	79.7	17,006	13,245	77.9	19,098	14,899	78.0	21,366	16,625	77.8	21,984	17,079	77.7
	Highest socio-economic status	9,850	7,871	79.9	14,226	11,277	79.3	16,294	12,914	79.3	17,601	13,813	78.5	18,532	14,643	79.0
	Missing	354	299	84.5	459	374	81.5	547	434	79.3	623	522	83.8	620	501	80.8

For peer review only

Table 1: extended table 1 for vitamin D.

		c														
		**2015**			**2016**			**2017**			**2018**			**2019**		
	No. of hospitals included	62			61			63			65			65		
	Total no. of patients with (at least) one vitamin D test	244,834			247,186			264,811			265,362			272,380		
	Total no. of patients with (at least) one vitamin D test with an associated DBC code	101,932			148,660			172,342			189,423			196,452		
	Total no. of patients with (at least) one non-indicated vitamin D test	84,414			124,162			143,408			157,431			162,345		
		**Total**	**Without (clear) indication**	**% without (clear indication**	**Total**	**Without (clear) indication**	**% without (clear indication**	**Total**	**Without (clear) indication**	**% without (clear indication**	**Total**	**Without (clear) indication**	**% without (clear indication**	**Total**	**Without (clear) indication**	**% without (clear indication**
**Male**	**No. of male patients receiving at least one vitamin-D test**	31,660	26,363	83.3	47,794	40,211	84.1	56,211	47,313	84.2	61,749	51,845	84.0	63,295	52,656	83.2
Age cat.	**18-29**	2,689	2,220	82.6	4,201	3,426	81.6	5,185	4,179	80.6	5,992	4,779	79.8	6,529	5,052	77.4
	**30-49**	8,684	7,658	88.2	13,086	11,536	88.2	15,614	13,701	87.7	16,964	14,750	86.9	17,483	15,123	86.5
	**50-69**	20,287	16,485	81.3	30,507	25,249	82.8	35,412	29,433	83.1	38,793	32,316	83.3	39,283	32,481	82.7
**Neighbou SES cat.**	***Lowest socio-economic status****	6,358	5,326	83.8	10,349	8,813	85.2	12,430	10,559	84.9	13,295	11,272	84.8	13,578	11,323	83.4
	Socio-economic status below average	6,758	5,584	82.6	9,964	8,342	83.7	12,199	10,201	83.6	13,249	11,079	83.6	13,818	11,496	83.2
	Average socio-economic status	6,331	5,233	82.7	9,311	7,755	83.3	10,677	8,851	82.9	12,292	10,249	83.4	12,287	10,092	82.1
	Socio-economic status above average	6,855	5,746	83.8	9,476	7,955	83.9	10,833	9,090	83.9	11,678	9,739	83.4	12,134	10,134	83.5
	Highest socio-economic status	5,164	4,309	83.4	8,405	7,092	84.4	9,735	8,315	85.4	10,825	9,151	84.5	11,025	9,238	83.8
	Missing	194	165	85.1	289	254	87.9	337	297	88.1	410	355	86.6	453	373	82.3
**Female**	**No. of female patients receiving at least one vitamin-D test**	70,272	58,051	82.6	100,866	83,951	83.2	116,131	96,095	82.7	127,674	105,586	82.7	133,157	109,689	82.4
Age cat.	**18-29**	8,105	7,198	88.8	11,485	10,030	87.3	14,038	12,120	86.3	16,091	13,864	86.2	17,271	14,855	86.0
	**30-49**	25,045	22,802	91.0	35,377	32,119	90.8	40,982	37,028	90.4	45,932	41,407	90.1	47,884	42,896	89.6
	**50-69**	37,122	28,051	75.6	54,004	41,802	77.4	61,111	46,947	76.8	65,651	50,315	76.6	68,002	51,938	76.4
**Neighbou SES cat.**	***Lowest socio-economic status****	15,024	12,710	84.6	22,104	18,873	85.4	26,254	22,286	84.9	28,219	23,903	84.7	29,182	24,613	84.3
	Socio-economic status below average	15,087	12,344	81.8	21,509	17,770	82.6	25,087	20,641	82.3	27,646	22,868	82.7	28,971	23,760	82.0
	Average socio-economic status	13,987	11,419	81.6	19,561	16,032	82.0	22,374	18,184	81.3	24,947	20,169	80.8	25,582	20,690	80.9
	Socio-economic status above average	14,302	11,739	82.1	19,405	15,976	82.3	21,663	17,576	81.1	24,007	19,542	81.4	25,303	20,528	81.1
	Highest socio-economic status	11,581	9,608	83.0	17,823	14,923	83.7	20,248	17,011	84.0	22,235	18,618	83.7	23,500	19,606	83.4
	Missing	291	231	79.4	464	377	81.3	505	397	78.6	620	486	78.4	619	492	79.5

For peer review only

Supplementary file S4: top 20 diagnose codes associated with non-indicated vitamin B12- and D-testing.

Table 1: Top 20 of diagnose codes associated with non-indicated vitamin B12-tests.

No. of non-indicated vitamin B12-tests	DTC specialism code	DTC diagnose code	DTC specialism description	DTC diagnose description
31,055	313	3	Internal medicine	Analysis general malaise/fatigue without diagnosis
30,807	313	222	Internal medicine	Diabetes mellitus with secondary complications
26,591	313	283	Internal medicine	Adiposity (obesity)
25,268	318	602	Gastro-enterology	Ulcerative colitis
20,667	313	221	Internal medicine	Diabetes mellitus without secondary complications
20,118	307	Z41	Obstetrics and gynaecology	Miscellaneous counselling during pregnancy
18,747	318	205	Gastro-enterology	Irritable bowel syndrome, diverticulosis
12,136	313	211	Internal medicine	Hypothyroidism
11,765	330	531	Neurology	Multiple sclerosis
11,168	313	6	Internal medicine	Analysis stomach complaints without diagnosis
10,003	313	223	Internal medicine	Diabetes mellitus chronic pump therapy
8,358	318	203	Gastro-enterology	Blood loss Gastrointestinal tract, unknown cause
8,057	324	701	Rheumatology	Arthralgia and/or myalgia
7,822	313	4	Internal medicine	Analysis anorexia, emaciation without diagnosis
7,770	318	204	Gastro-enterology	Chronic stomach-ache
7,425	330	402	Neurology	Miscellaneous cognitive and memory impairments
6,841	313	311	Internal medicine	Hypertension
6,758	324	101	Rheumatology	Rheumatoid arthritis
6,688	313	324	Internal medicine	Chronic kidney insufficiency eGFR 30-60 ml/min
6,339	313	299	Internal medicine	Miscellaneous endocrine and metabolic conditions

For peer review only

Table 2: Top 20 of diagnose codes associated with non-indicated vitamin D-tests

**No. of non-indicated vitamin D-tests**	**DTC specialism code**	**DTC diagnose code**	**DTC specialism description**	**DTC diagnose description**
38,213	303	342	General surgery	Morbid obesity BMI >45
34,842	313	283	Internal medicine	Adiposity (obesity)
30,175	307	Z41	Obstetrics and gynaecology	Miscellaneous counselling during pregnancy
29,857	324	701	Rheumatology	Arthralgia and/or myalgia
25,072	318	602	Gastro-enterology	Ulcerative colitis
23,263	313	3	Internal medicine	Analysis general malaise/fatigue without diagnosis
21,723	313	222	Internal medicine	Diabetes mellitus with secondary complications
21,076	324	101	Rheumatology	Rheumatoid arthritis
19,013	313	811	Internal medicine	Malignancy mama
18,346	330	531	Neurology	Multiple sclerosis
17,208	313	324	Internal medicine	Chronic kidney insufficiency eGFR 30-60 ml/min
15,050	313	461	Internal medicine	HIV infection with treatment indication
12,718	303	341	General surgery	Morbid obesity BMI <45
12,354	313	76	Internal medicine	Recipient kidney transplant
12,155	324	707	Rheumatology	Fibromyalgia
12,025	313	221	Internal medicine	Diabetes mellitus without secondary complications
11,182	313	211	Internal medicine	Hypothyroidism
10,926	313	311	Internal medicine	Hypertension
9,960	322	1201	Respiratory medicine	Asthma
8,639	318	205	Gastro-enterology	Irritable bowel syndrome, diverticulosis

For peer review only

Supplementary file S5: overview of the raw data and proportions of the total outpatient visits that received a vitamin B12- and D- test for each of the included hospitals over 2019.

Table 1: Proportion (%) of outpatient visits that received a vitamin B12-tests without a clear indication,

No,	Hospital ID	Total no, of outpatient visits recorded in 2019	Total no, of vitamin B12 tests without a clear indication	Proportion (%) of outpatient visits that received a vitamin B12 test without clear indication
1	48	362,887	2	0.00
2	40	580,678	8	0.00
3	66	127,984	52	0.04
4	41	576,063	263	0.05
5	11	345,279	407	0.12
6	23	568,860	716	0.13
7	38	289,822	400	0.14
8	39	565,276	1,094	0.19
9	46	358,602	1,693	0.47
10	1	344,543	1,953	0.57
11	62	227,934	1,324	0.58
12	25	165,640	985	0.59
13	50	436,838	2,808	0.64
14	52	508,460	4,723	0.93
15	35	156,620	1,485	0.95
16	68	133,631	1,387	1.04
17	12	494,601	6,081	1.23
18	67	153,812	1,915	1.25
19	69	318,615	4,021	1.26
20	71	408,355	5,680	1.39
21	55	194,373	2,792	1.44
22	60	421,743	6,422	1.52
23	6	384,801	6,032	1.57
24	59	395,402	6,608	1.67
25	27	389,827	7,214	1.85

For peer review only

26	54	383,123	7,109	1.86
27	36	363,166	7,477	2.06
28	16	163,322	5,133	3.14
29	19	131,553	4,361	3.32
30	57	280,541	9,441	3.37
31	63	427,252	15,000	3.51
32	29	216,550	7,672	3.54
33	4	315,289	11,366	3.60
34	65	237,265	8,670	3.65
35	31	309,391	11,457	3.70
36	28	113,439	4,239	3.74
37	42	268,758	10,220	3.80
38	45	384,873	14,745	3.83
39	64	342,101	13,120	3.84
40	17	155,415	6,008	3.87
41	30	370,550	14,413	3.89
42	7	384,199	14,944	3.89
43	33	606,974	23,806	3.92
44	20	147,262	5,997	4.07
45	49	259,604	10,903	4.20
46	26	182,212	7,660	4.20
47	47	185,195	7,875	4.25
48	56	192,410	8,244	4.28
49	34	429,614	18,618	4.33
50	10	276,454	12,102	4.38
51	8	223,140	9,775	4.38
52	14	567,264	25,389	4.48
53	70	253,998	11,381	4.48
54	24	654,329	29,546	4.52
55	43	536,979	24,622	4.59
56	51	443,539	21,314	4.81
57	44	356,220	17,236	4.84

For peer review only

58	58	154,585	7,521	4.87
59	15	165,151	8,097	4.90
60	13	466,598	23,040	4.94
61	5	401,794	20,260	5.04
62	37	171,709	8,665	5.05
63	32	337,152	19,183	5.69
64	61	174,166	10,466	6.01
65	3	113,329	7,276	6.42
66	22	251,812	17,093	6.79
67	21	186,579	13,004	6.97
68	2	379,408	104,750	27.61

For peer review only

Table 2: Proportion (%) of outpatient visits that received a vitamin D tests without a clear indication,

No,	Hospital ID	Total no, of outpatient visits recorded in 2019	Total no, of vitamin D determinations without a clear indication	Proportion (%) of outpatient visits that received a vitamin D determination without clear indication
1	48	362,887	0	0.00
2	40	580,678	0	0.00
3	38	289,822	0	0.00
4	41	576,063	576,063	0.02
5	66	127,984	127,984	0.02
6	11	345,279	345,279	0.06
7	23	568,860	568,860	0.08
8	39	565,276	565,276	0.20
9	50	436,838	436,838	0.28
10	1	344,543	344,543	0.38
11	68	133,631	133,631	0.48
12	62	227,934	227,934	0.54
13	25	165,640	165,640	0.59
14	52	508,460	508,460	0.64
15	46	358,602	358,602	0.71
16	35	156,620	156,620	0.84
17	67	153,812	153,812	0.89
18	55	194,373	194,373	1.35
19	60	421,743	421,743	1.35
20	6	384,801	384,801	1.49
21	12	494,601	494,601	1.52
22	71	408,355	408,355	1.75
23	59	395,402	395,402	2.36
24	36	363,166	363,166	2.39
25	28	113,439	113,439	2.64
26	30	370,550	370,550	2.65
27	27	389,827	389,827	2.75

For peer review only

28	54	383,123	383,123	2.88
29	64	342,101	342,101	3.04
30	69	318,615	318,615	3.07
31	56	192,410	192,410	3.20
32	20	147,262	147,262	3.20
33	29	216,550	216,550	3.23
34	26	182,212	182,212	3.30
35	8	223,140	223,140	3.39
36	47	185,195	185,195	3.41
37	17	155,415	155,415	3.41
38	45	384,873	384,873	3.45
39	57	280,541	280,541	3.50
40	19	131,553	131,553	3.50
41	33	606,974	606,974	3.67
42	5	401,794	401,794	4.05
43	24	654,329	654,329	4.06
44	34	429,614	429,614	4.30
45	65	237,265	237,265	4.58
46	22	251,812	251,812	4.80
47	58	154,585	154,585	4.97
48	42	268,758	268,758	5.00
49	15	165,151	165,151	5.07
50	3	113,329	113,329	5.07
51	16	163,322	163,322	5.27
52	51	443,539	443,539	5.44
53	13	466,598	466,598	5.46
54	4	315,289	315,289	5.68
55	21	186,579	186,579	5.84
56	49	259,604	259,604	5.91
57	32	337,152	337,152	5.91
58	70	253,998	253,998	6.17
59	63	427,252	427,252	6.31

For peer review only

60	61	174,166	174,166	6.33
61	14	567,264	567,264	6.50
62	10	276,454	276,454	6.83
63	31	309,391	309,391	7.04
64	44	356,220	356,220	7.42
65	37	171,709	171,709	8.37
66	43	536,979	536,979	9.00
67	7	384,199	384,199	9.37
68	2	379,408	379,408	34.79

For peer review only

Supplementary file 6: Density plots and Shapiro-Wilk normality test outcomes and the correlation test outcomes (with and without the outlierincluded)

Table 1: Normality testing outcomes

Variable	Shapiro-Wilk normality test	P-value (significance p<0.05)	Outcome
Rate of non-indicated vitamin-B12 tests	0.58552	1.46e-12	Not normally distributed
Rate of non-indicated vitamin-D tests	0.58001	1.19e -12	Not normally distributed

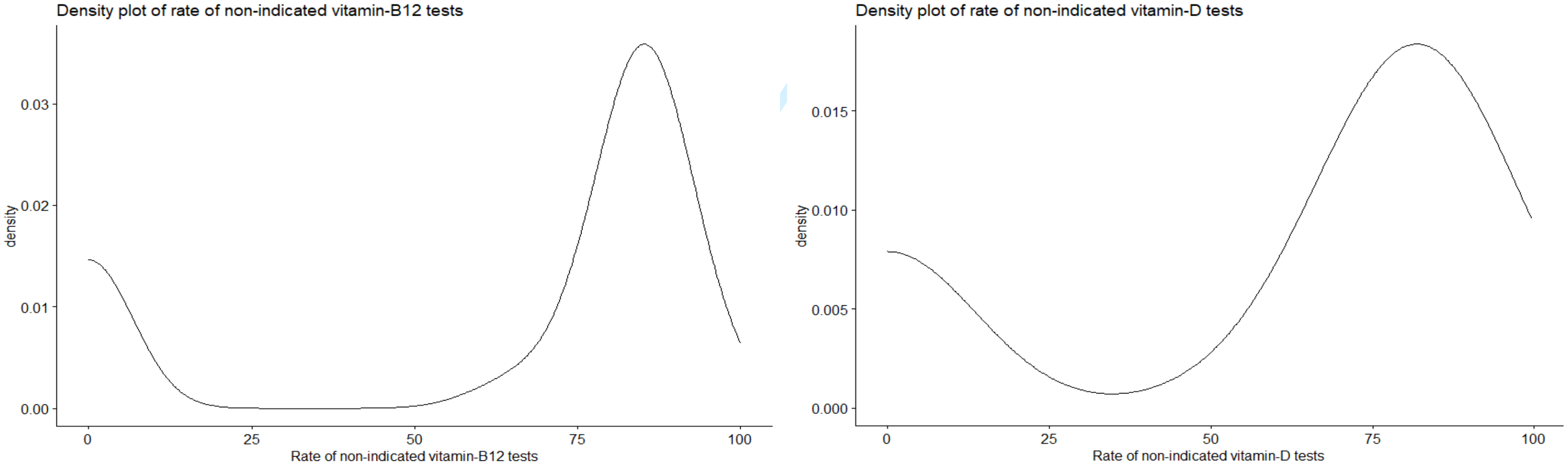

Figure 1: Density plots of rates of vitamin B12- and D-testing among the hospitals included

Table 2: Spearman's rank correlation Rho test outcomes

Rho	P-value
0.8571538	P < 2.2e-16

Correlation analysis outcome before removal of the outlier

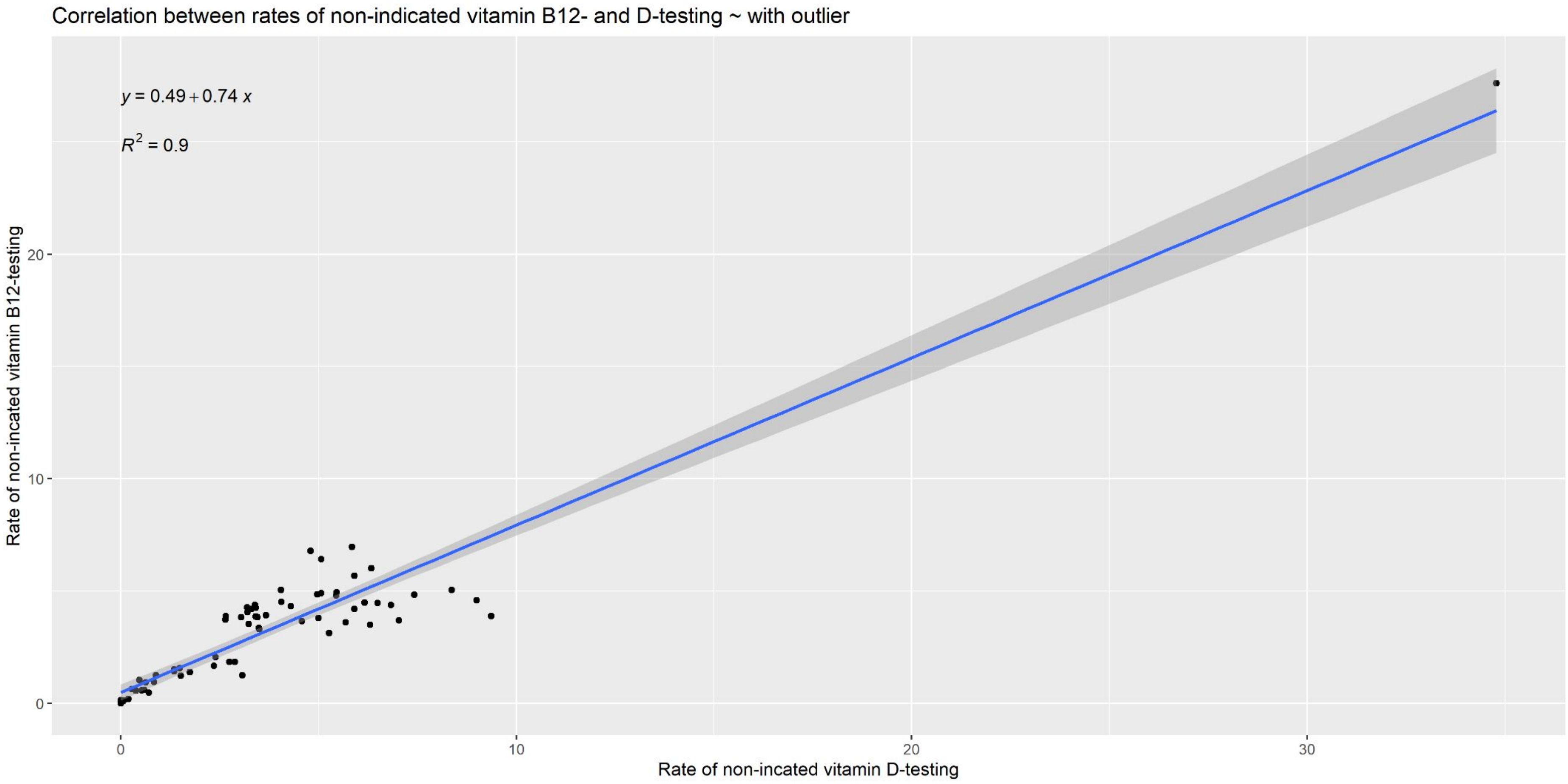

Figure 2: rates of non-indicated vitamin B12- and D-testing before removal of the outlier

STROBE Statement—Checklist of items that should be included in reports of ***cross-sectional studies***

	Item No	Recommendation	Page No
**Title and abstract**	1	(*a*) Indicate the study's design with a commonly used term in the title or the abstract	1
		(*b*) Provide in the abstract an informative and balanced summary of what was done and what was found	2
**Introduction**			
Background/rationale	2	Explain the scientific background and rationale for the investigation being reported	4 - 5
Objectives	3	State specific objectives, including any prespecified hypotheses	4 - 5
**Methods**			
Study design	4	Present key elements of study design early in the paper	5 - 7
Setting	5	Describe the setting, locations, and relevant dates, including periods of recruitment, exposure, follow-up, and data collection	5
Participants	6	(*a*) Give the eligibility criteria, and the sources and methods of selection of participants	5 - 7
Variables	7	Clearly define all outcomes, exposures, predictors, potential confounders, and effect modifiers. Give diagnostic criteria, if applicable	5 - 7
Data sources/ measurement	8*	For each variable of interest, give sources of data and details of methods of assessment (measurement). Describe comparability of assessment methods if there is more than one group	5 - 7
Bias	9	Describe any efforts to address potential sources of bias	N.A.
Study size	10	Explain how the study size was arrived at	N.A.
Quantitative variables	11	Explain how quantitative variables were handled in the analyses. If applicable, describe which groupings were chosen and why	5 - 7
Statistical methods	12	(*a*) Describe all statistical methods, including those used to control for confounding	5 - 7
		(*b*) Describe any methods used to examine subgroups and interactions	5 - 7
		(*c*) Explain how missing data were addressed	5 - 7
		(*d*) If applicable, describe analytical methods taking account of sampling strategy	5 - 7
		(*e*) Describe any sensitivity analyses	5 - 7
**Results**			
Participants	13*	(a) Report numbers of individuals at each stage of study—eg numbers potentially eligible, examined for eligibility, confirmed eligible, included in the study, completing follow-up, and analysed	8 - 11
		(b) Give reasons for non-participation at each stage	N.A.
		(c) Consider use of a flow diagram	N.A.
Descriptive data	14*	(a) Give characteristics of study participants (eg demographic, clinical, social) and information on exposures and potential confounders	8 - 11
		(b) Indicate number of participants with missing data for each variable of interest	8 - 11
Outcome data	15*	Report numbers of outcome events or summary measures	8 - 11
Main results	16	(*a*) Give unadjusted estimates and, if applicable, confounder-adjusted estimates and their precision (eg, 95% confidence interval). Make clear which confounders were adjusted for and why they were included	8 - 11

For peer review only - http://bmjopen.bmj.com/site/about/guidelines.xhtml

		(*b*) Report category boundaries when continuous variables were categorized	N.A.
		(*c*) If relevant, consider translating estimates of relative risk into absolute risk for a meaningful time period	N.A.
Other analyses	17	Report other analyses done—eg analyses of subgroups and interactions, and sensitivity analyses	9 – 10
**Discussion**			
Key results	18	Summarise key results with reference to study objectives	12
Limitations	19	Discuss limitations of the study, taking into account sources of potential bias or imprecision. Discuss both direction and magnitude of any potential bias	13 - 15
Interpretation	20	Give a cautious overall interpretation of results considering objectives, limitations, multiplicity of analyses, results from similar studies, and other relevant evidence	12 - 16
Generalisability	21	Discuss the generalisability (external validity) of the study results	15 - 16
**Other information**			
Funding	22	Give the source of funding and the role of the funders for the present study and, if applicable, for the original study on which the present article is based	17

*Give information separately for exposed and unexposed groups.

Note: An Explanation and Elaboration article discusses each checklist item and gives methodological background and published examples of transparent reporting. The STROBE checklist is best used in conjunction with this article (freely available on the Web sites of PLoS Medicine at http://www.plosmedicine.org/, Annals of Internal Medicine at http://www.annals.org/, and Epidemiology at http://www.epidem.com/). Information on the STROBE Initiative is available at www.strobe-statement.org.

For peer review only

For peer review only - http://bmjopen.bmj.com/site/about/guidelines.xhtml

BMJ Open

Non-indicated vitamin B12- and D-testing among Dutch hospital clinicians: a cross-sectional analysis in data-registries

Journal:	*BMJ Open*
Manuscript ID	bmjopen-2023-075241.R1
Article Type:	Original research
Date Submitted by the Author:	17-Jan-2024
Complete List of Authors:	Müskens, Joris; Radboudumc IQ healthcare, IQ healthcare<br>Kool, Rudolf; Radboud university medical centre, Scientific Institute for Quality of Healthcare (IQ Healthcare)<br>Westert, Gert; Radboud university medical centre, Scientific Institute for Quality of Healthcare (IQ Healthcare)<br>Zaal, Maarten; Information analyst at Dutch Hospital Data<br>Muller, Hein; Dutch Hospital Data<br>Atsma, Femke; Radboud University Nijmegen Medical Centre, Scientific Institute for Quality of Healthcare<br>van Dulmen, SA; Radboud University Medical Center, IQ healthcare
**Primary Subject Heading**:	Health services research
Secondary Subject Heading:	Health services research
Keywords:	Quality in health care < HEALTH SERVICES ADMINISTRATION & MANAGEMENT, Hospitals, Public, Clinical Decision-Making, Retrospective Studies

SCHOLARONE™
Manuscripts

I, the Submitting Author has the right to grant and does grant on behalf of all authors of the Work (as defined in the below author licence), an exclusive licence and/or a non-exclusive licence for contributions from authors who are: i) UK Crown employees; ii) where BMJ has agreed a CC-BY licence shall apply, and/or iii) in accordance with the terms applicable for US Federal Government officers or employees acting as part of their official duties; on a worldwide, perpetual, irrevocable, royalty-free basis to BMJ Publishing Group Ltd ("BMJ") its licensees and where the relevant Journal is co-owned by BMJ to the co-owners of the Journal, to publish the Work in this journal and any other BMJ products and to exploit all rights, as set out in our licence.

The Submitting Author accepts and understands that any supply made under these terms is made by BMJ to the Submitting Author unless you are acting as an employee on behalf of your employer or a postgraduate student of an affiliated institution which is paying any applicable article publishing charge ("APC") for Open Access articles. Where the Submitting Author wishes to make the Work available on an Open Access basis (and intends to pay the relevant APC), the terms of reuse of such Open Access shall be governed by a Creative Commons licence – details of these licences and which Creative Commons licence will apply to this Work are set out in our licence referred to above.

Other than as permitted in any relevant BMJ Author's Self Archiving Policies, I confirm this Work has not been accepted for publication elsewhere, is not being considered for publication elsewhere and does not duplicate material already published. I confirm all authors consent to publication of this Work and authorise the granting of this licence.

For peer review only

Non-indicated vitamin B12- and D-testing among Dutch hospital clinicians: a cross-sectional analysis in data registries

1. Joris L.J.M. Müskens, PhD student at Radboud Institute for Health Sciences, IQ healthcare, Radboud University Medical Centre, Nijmegen, The Netherlands
2. Rudolf B. Kool, PhD MD, Professor of Appropriate Care and program manager of "To do or not to do?" at Radboud Institute for Health Sciences, IQ healthcare, Radboud University Medical Centre, Nijmegen, The Netherlands
3. Gert P. Westert, PhD, Professor of Health services research and director of IQ healthcare at Radboud Institute for Health Sciences, IQ healthcare, Radboud University Medical Centre, Nijmegen, The Netherlands
4. Maarten Zaal, PhD, Information analyst at Dutch Hospital Data, Utrecht, The Netherlands
5. Hein Muller, MD, PhD MD, FACP, independent advisor at the Dutch Hospital Data, Utrecht, The Netherlands.
6. Femke Atsma, PhD, Senior epidemiologist and methodologist, IQ healthcare, Radboud University Medical Centre, Nijmegen, The Netherlands
7. Simone A. van Dulmen, PhD, senior researcher and project manager at Radboud Institute for Health Sciences, IQ healthcare, Radboud University Medical Centre, Nijmegen, The Netherlands

Corresponding author: Joris L.J.M. Müskens, Joris.muskens@radboudumc.nl

Postal address: Radboud Institute for Health Sciences, Scientific Center for Quality of Healthcare (IQ healthcare), Postbus 9101, 6500 HB Nijmegen (route 160), The Netherlands

Word count: 3,518

For peer review only

Abstract

Objectives: to assess the extent of non-indicated vitamin B12- and D-testing among Dutch clinicians and its variation among hospitals.

Design: cross-sectional study using registration data from 2015-2019.

Participants: patients aged 18-70 years between that received a vitamin B12- or D-test.

Primary and secondary outcome measures: the proportion of non-indicated vitamin B12- and D-testing among Dutch clinicians and its variation between hospitals (n=68) over 2015 to 2019.

Results: Between 2015-2019 at least 79.0% of all vitamin B12- and 82.0% of vitamin D-tests lacked a clear indication. The number of vitamin B12-tests increased by 2.0% over the examined period, while the number of D-tests increased by 12.2%. The proportion of the unexplained variation in non-indicated vitamin B12- and D-tests that can be ascribed to differences between hospitals remained low. ICCs ranged between 0.072-0.085 and 0.081-0.096 for non-indicated vitamin-B12 and D tests respectively. The included casemix variables patient age, gender, Socioeconomic Status and hospital size only accounted for a small part of the unexplained variation in non-indicated testing. Additionally, a significant correlation was observed in non-indicated vitamin B12- and D-testing among the included hospitals.

Conclusion: Hospital clinicians order vitamin B12- and D-tests without a clear indication on a large scale. Only a small proportion of the unexplained variation could be attributed to differences between hospitals.

Key words: Low-value care, Diagnostic Techniques and Procedures, quality of healthcare

For peer review only

Strengths and limitations:

- This study has examined the ordering and appropriateness of vitamin B12- and D-testing among hospital clinicians and its variation using data over several consecutive years.
- The use of a detailed nationally representative database, allowed us to generate an accurate and reliable overview of the extent of (non-indicated) vitamin B12- and D-testing among exclusively hospital clinicians within the Dutch healthcare system.
- Additionally, the use of registration data derived directly from hospital registration systems, enabled us to accurately distinguish appropriate from inappropriate vitamin testing using diagnosis codes rather than the proxy of testing interval.

For peer review only

Introduction

Low-value diagnostic testing is a serious problem in most health care systems [1]. Low-value care is defined as care that offers no net benefit for the patient and which can be associated with harmful outcomes and wasteful spending. [2-4] Within diagnostic testing, studies show that both vitamin B12- and D-tests are frequently ordered within medical practice [5-8]. However, there are only a few indications that justify the ordering of a vitamin B12- or D-test which are described in international guidelines. Among healthy adults, almost no indications for vitamin B12- and D-testing exist. [9, 10] International guidelines for hospital clinicians clearly state that vitamin B12- and D-tests should only be ordered in specific scenarios, such as in patients with Coeliac or Crohn disease, and not in patients with only vague complaints. [9, 11-14]. Assessments indicate that as much as 77% of vitamin B12- and 91% of vitamin D-tests could lack an indication. [7, 15-23]

Studies show that between 8% and 28% of vitamin B12-tests, and between 6% and 91% of vitamin D-tests could be non-indicated. Most of these studies have been conducted among general practitioners (GPs). [17, 19, 21, 23] Only a few studies have clearly specified that their assessment of the volume of non-indicated vitamin testing concerned hospital clinicians alone. Two studies from Italy reported that on average 17% and 6% of vitamin B12- and D-tests were potentially non-indicated. [7, 18] However, these assessments used data from a single hospital and therefore do not provide a representative overview. Furthermore, these studies based their assessment of appropriateness on a proxy such as testing interval, and not directly by examining the diagnose for which the test was requested.

In order to address non-indicated vitamin B12- and D-testing, an accurate assessment of the magnitude and variation of non-indicated testing is needed. We therefore aimed to assess both the volume and proportions of patients aged between 18 and 70 years old that potentially received non-indicated vitamin B12- and D-tests among Dutch hospital clinicians between 2015 and 2019 using clinical registration data. We also examined the hospital variation in non-indicated vitamin B12- and

For peer review only

D-testing in order to identify opportunities for improvement. The proportion of outpatient visits receiving a non-indicated vitamin B12- or D-test among the hospitals included, and the diagnose codes often associated with vitamin B12- and D-testing over 2019 were also explored. In doing so, we aimed to gain more insight into the volume of non-indicated vitamin testing among hospital clinicians and the diagnoses underlying such testing.

Methods

Design and database

We conducted a cross-sectional study using registration data among Dutch hospital clinicians between 2015 and 2019. Data were obtained from the Dutch National Basic Hospital Care Registration (*Landelijke Basisregistratie Ziekenhuiszorg, LBZ)*. [24] The LBZ contains medical, financial and administrative information from all patients undergoing treatment in any Dutch hospital. All vitamin B12- and D- tests ordered by clinicians in Dutch hospitals over the examined time period were extracted; including the associated diagnosis codes, patient age, gender and Socioeconomic Status (SES) and hospital size. After consulting paediatricians and geriatricians, we limited our analysis to patients aged 18 to 70 years old. Both paediatricians and geriatricians indicated that for patients below 18 and above 70, there are many regional screenings protocols which also often vary between hospitals. Patients were assigned to one of three age categories; 18-29, 30-49, 50-70. SES scores were derived from a table containing SES-scores on the level of four-digit postal codes as published by the Dutch Institute for Social Research (SCP) in 2017. [25] Patients were also assigned to one of six SES categories, based on quintiles calculated with the SES information from all Dutch neighbourhoods. Hospital size was operationalized by assigning a hospital to one of three categories (small/medium/large) based on tertiles calculated using the amount of outpatient visits encountered over 2019.

Patient and public involvement

For peer review only - http://bmjopen.bmj.com/site/about/guidelines.xhtml

No patients or members of the public were directly involved in the study. Owing to the nature of this study and data privacy constraints, no patients or members of the public were involved in the study design, analysis, interpretation of data, or revision of the manuscript.

Analysis of trends in proportions of non-indicated vitamin B12- and D-testing among hospital clinicians.

For the assessment of the proportion of justified indication versus non-indicated vitamin B12- and D-testing, we used a service lens, as previously described by Chalmers et al. [26] This entails that all vitamin B12- or D-tests ordered were included in our denominator, and all vitamin B12- and D-tests ordered with no indication in our numerator. For our distinction of non-indicated vitamin testing, we followed several steps. First, all recommendations regarding vitamin B12- or D-testing were extracted from the relevant guidelines. Initially, we reviewed the Dutch guidelines of hospital clinicians published by the federation of medical specialists for indications for vitamin B12- and D-testing. [10] We managed to identify little to no recommendations concerning indications regarding the use of vitamin B12- or D-tests. We therefore chose to supplement these with indications derived from Dutch general practitioners guidelines. [13, 14] Second, the ICD10 codes corresponding to the diagnoses which warrant the ordering of a vitamin B12- or D-test were collected from these recommendations. Third, the resulting list of ICD10 codes was reviewed by the involved experts to prevent missing relevant codes or diagnoses. We consulted two expert clinicians (an internal medicine physician and haematologist) in the process of generating the list of indications justifying a vitamin B12- or D-test. Fourth, after completion the list of ICD10 codes, all Clinical Classification Software (CCS) codes associated to these ICD10 codes were extracted from the LBZ database. Subsequently, all Diagnosis-Treatment Combination (DTC) codes associated to the list of relevant CCS codes were extracted. The resulting list was, again, checked for completeness before starting with the assessment of indicated vitamin tests. This process was repeated until all researchers and clinicians agreed on the accuracy of the list of indications justifying vitamin B12- and D-testing.

Supplementary file S1 lists of ICD10 and DTC codes used to determine the appropriateness of the identified vitamin B12- or D-tests. It also contains a description of how we identified and linked the identified ICD10 codes to each of the patients included. In an effort to provide potential handles for the design of interventions, we also examined which diagnosis codes were frequently associated with non-indicated tests.

Assessment of hospital variation in non-indicated vitamin B12- and D-testing.

Hospital variation in non-indicated vitamin B12- and D- testing was assessed using a multilevel logistic regression analysis, with a random effect for hospital. Separate models per year were made to assess whether the variation in non-indicated vitamin B12- and D-testing was robust over time. Generalized variance inflation factors (GVIF) were calculated to test for collinearity among the included variables before multilevel analysis was conducted (supplementary file S2). Models were adjusted for the casemix variables: patient age, gender, Socioeconomic Status (SES), and hospital size. We corrected for patient age, gender and SES, as previous research showed that these affect the amount of care that patients require, receive and have access to. [27-31] We included a proxy for hospital size (e.g. the total number of outpatient visits in each year) while recent evidence shows that larger healthcare providers tend to provide more low-value care. [32] Vitamin tests conducted in patients with a missing SES score or DTC code were excluded from the analysis. Intraclass correlation coefficients (ICCs) were calculated to assess which part of the unexplained variation in non-indicated vitamin B12- and D-testing could be ascribed to differences between the included hospitals, using the method of Snijders and Bosker to assess the error variance. [33]

Correlation in non-indicated vitamin testing over 2019

Additionally, we also examined whether a correlation existed between the proportions of outpatient visits that received a non-indicated vitamin B12- or D-test over 2019 among the hospitals included. Correlations were assessed using the Pearson correlation coefficient for normally distributed

150 variables and the Spearman correlation coefficient for non-normally distributed variables. Normality

151 was assessed using both density plots and the Shapiro-Wilk test.

For peer review only

Results

Volume vitamin tests among Dutch hospital clinicians.

Table 1 provides a general overview of the population characteristics of the population included in our study. Between 2015 and 2019, the number of vitamin B12- and D-tests ordered by clinicians increased by 2.0% (from 275,032 to 280,522) and 12.2% (from 300,013 to 336,736) respectively. A similar trend was also observed in the proportion of patients that received at least one vitamin B12- or D-test, increasing by 2.5% and 11.3% over the examined period. The amount of vitamin B12- and D-tests ordered among women remained almost twice as high compared to men over the entire period examined. Table 2 provides an overview of the outcomes, supplementary file S3 contains a more detailed breakdown by gender, age- and SES-groups on the patient level. The number of patients with at least one vitamin test increases rapidly with age. The included patients, with at least one vitamin B12 or D-test, showed to be more equally distributed over the SES categories. Only in the highest SES category a slight decrease in the number of patients with a vitamin determination was observed.

Table 1: overview of the study population characteristics of the used population over the entire period examined (2015 – 2019).

For peer review only - http://bmjopen.bmj.com/site/about/guidelines.xhtml

Non-indicated testing

Between 2015 and 2019 around 78% of the vitamin B12- tests conducted among patients between age 18 and 70, with an registered DTC code, lacked an indication. In case of vitamin D-testing, around 82% of determinations had no clear indication. Although the number of vitamin tests is higher among women, no large differences in proportions of non-indicated testing were observed between genders. In case of both age and SES, the proportion of patients with a non-indicated vitamin B12- or D-test remains relatively constant across all groups over the study period (supplementary file S3). With the proportion of non-indicated testing remaining around 80.0% for vitamin B12 and 83.5% for vitamin D across the different age and SES. Our analysis of diagnose codes that are most often associated with non-indicated vitamin B12- and D-testing revealed that tests are ordered for various reasons. Similar diagnostic codes were associated with both non-indicated vitamin B12 and D tests, including general malaise, fatigue without diagnosis, and ulcerative colitis. Supplementary file S4 contains an overview of the top 20 diagnose codes for both vitamin B12- and D-tests.

Hospital variation in non-indicated vitamin B12- and D-tests among Dutch hospital clinicians

The ICCs of the models uncorrected for casemix remained around 9% (ranging from 8,3% to 9,5%) and 9,5% (ranging from 8,5% to 10,1%) for the vitamin B12- and D-models over time. The ICCs of the casemix corrected vitamin B12- and D-models remained stable around 8.0% (ranging between 7,2 and 8,5%) and 9% (ranging between 8,1% and 9.6%), respectively, throughout the examined period. Supplementary file S5 contains the ICCs of all models. Casemix correction minimally impacted the calculated ICCs in case of both the vitamin B12- and D-models. The proportion of outpatient visits receiving a non-indicated vitamin B12- or D-test over 2019 varied widely among the hospitals included, ranging from 0.0% to 27.6% for vitamin B12 and 0.02% to 34.8% for vitamin D (see figure 1 and supplementary file S6).

Figure 1: proportion of all outpatient visits at each of the included hospitals that received a low-value vitamin (A) B12- or (B) D- test over 2019 (n=68).

For peer review only

Correlation in proportions of non-indicated vitamin B12- and D-testing

Normality testing (and inspection of density plots) revealed that both the proportions of vitamin B12- and D-testing observed among the hospitals are non-normally distributed. The subsequent correlation analysis revealed the presence of a significant positive correlation (Rho: 0.86, $p < 0.001$) between the proportions of non-indicated vitamin B12- and D-testing among the included hospitals. Supplementary file S7 contains the density plots, normality tests results and correlation analysis outcomes for both non-indicated vitamin B12- and D-tests.

For peer review only

Table 2: Overview of vitamin B12 and D tests performed among hospital clinicians over the period examined (2015 – 2019).

For peer review only

Discussion

Between 2015 and 2019, around 78.0% and 82.0% of vitamin B12- and D-tests ordered by Dutch hospital clinicians in patients aged 18-70 lacked a clear indication. The total number of vitamin B12-tests ordered increased by 2.0%, while the total number of vitamin D-tests increased by 12.2%. Although the total number of vitamin determinations increased, the proportion of patients with at least one vitamin test remained relatively constant (around 80% for B12 and 83% for D). Women received approximately twice as many vitamin B12- and D-tests, as well as non-indicated tests, over the examined period compared to men. Our analysis of hospital variation in non-indicated vitamin B12- and D-testing revealed a moderate hospital variation. Furthermore, only a relatively small part of the unexplained variation in non-indicated vitamin B12- and D- testing could be ascribed to differences between the hospitals included. Suggesting that the problem of non-indicated vitamin testing is present among all hospitals. Correlation analysis over 2019 revealed a fairly strong positive (Rho: 0.86) correlation between the rates of non-indicated vitamin B12- and D-testing among the hospitals included.

Comparison to existing literature.

The proportion of non-indicated vitamin B12- and D-testing among clinicians found in our study (78.0% and 82.0% respectively) are substantially larger than the proportions reported by other studies. For example, two studies from Italy conducted among hospital clinicians report that on average 17.0% and 6.0% of vitamin B12- and D-tests could potentially be non-indicated. [7, 18] Which is substantially lower than the proportions observed in our study. Our findings are more in line with assessments that are not limited to hospital clinicians. Hence, studies conducted among general practitioners indicate that between 8.0% and 28.0% of vitamin B12- [7, 15, 16, 18] and between 7.0% and 91.0% of vitamin D-tests could be considered non-indicated. [7, 15, 19, 21, 23] Among these studies, only Naugler et al. and Gonalez-Chicas reported proportions of non-indicated vitamin D testing which is similar to ours. Naugler et al., found that following an intervention the prevalence of

non-indicated vitamin D-testing decreased by 91.4%. [19] While Gonzalez-Chica reported 76,5% of vitamin D-tests to be non-indicated after introduction of new Medicare criteria for rebates. Besides population differences (e.g. GPs versus hospital clinicians), varying definitions of non-indicated testing and methods used could also account for the large differences in assessment outcomes. For example, both our study and that of Naugler et al. utilized diagnosis codes to distinguish the appropriateness of vitamin testing. [19] Most assessments of the appropriateness of vitamin testing performed to date used testing intervals or laboratory results to discern non-indicated testing, as did Gonzalez-Chica. [23] This methodological difference might explain the large differences in assessment outcomes. Furthermore, the studies that did not limit their assessment to vitamin testing among GPs, were often conducted within a single hospital. [7, 18] Moreover, most studies that assessed vitamin B12- or D-testing do not specify which type of physicians (GP/hospital clinicians) or vitamin B12- and D-tests were included, making comparison to our study challenging.

The absence of clear recommendations regarding the *"appropriate"* use of vitamin testing in clinician guidelines, does not aid clinicians to appropriately order vitamin testing. The current guidelines for hospital clinicians offer little direction on the appropriate use of vitamin B12 or D tests, leaving clinicians with little guidance when deciding whether to order such tests. These difficulties are magnified by the large knowledge gaps regarding the exact roles of vitamin B12 and D within the human body and its metabolism. [34-37] The combination of these factors may provide an explanation for the high proportion of non-indicated testing. Hence, due to the lack of a clear understanding of the roles of vitamin B12 and D within the body and the fear of missing a diagnosis, clinicians may engage in defensive behaviour resulting in the ordering of vitamin tests.

Strengths and limitations.

A strength of this study is its novelty in examining the ordering and appropriateness of vitamin B12- and D-testing among hospital clinicians and its variation over several consecutive years. A second strength is that we used a detailed nationally representative database. This allowed us to generate

an accurate and reliable overview of the extent of vitamin B12- and D-testing among solely hospital clinicians. Furthermore, it also enabled us to accurately distinguish appropriate from inappropriate vitamin testing using diagnose codes rather than the proxy of testing interval.

However, our study is also prone to limitations. First, we based our distinction of appropriateness mainly on expert opinion and indications derived from the Dutch GP guidelines, as no universal guideline regarding vitamin testing among hospital clinicians exists. We therefore might have misclassified some of the tests from clinicians as being non-indicated. However, we tried to minimize the risk of misclassification by closely collaborating with the involved experts with respect to the creation of the list of indications. Additionally, some differences exist between international and Dutch guidelines regarding indications for vitamin B12- and D-testing. For example, guidelines from the US indicate that vitamin B12-testing is considered indicated in patients with cognitive impairments or dementia. [38] However, these are not considered as an indication according to the nationwide guidelines published by the Dutch federation of medical specialists. [10] Potentially, such recommendations could exist in local protocols of Dutch hospitals, which unfortunately are not publicly available. This makes it difficult to compare international assessment outcomes to our study, while subtle differences between guidelines might cause large differences in the used criteria of appropriateness (and subsequently the reported outcomes).

Second, the use of DTC codes enabled us to accurately distinguish the appropriateness of vitamin tests, but also has a drawback. DTC diagnosis codes are generally less specific compared to the ICD10 codes described in the used guidelines. We therefore might have misclassified some vitamin tests as being appropriate. Furthermore, although DTC diagnosis codes provide a lot of insight into the diagnosis associated with practices, their registration is prone to misregistration. [39] Clinicians have a vast amount of (often similar) DTC-codes to choose from when registering a diagnosis code, thereby adding another layer of complexity to the correct registration of diagnoses. Furthermore, DTC codes are updated as the patient passes through the healthcare system. The registered DTC

code therefore does not necessarily represent the initial reason (or diagnosis) for which the vitamin determination was ordered, but rather reflects the final diagnosis.

Third, some Dutch hospitals outsource their tests to external commercial laboratories, which are not registered in the LBZ. Our estimate of vitamin testing by clinicians therefore is not complete. However, according to the registration of the national statistical office, Statistics Netherlands (CBS), 82 hospitals were active in 2019. Since we were able to include data from the majority of the hospitals in the Netherlands in our study (68/82, e.g. 83.0% of all hospitals), we do not expect to have missed much in our analysis. [40]

Implications for research and practice

Our assessment reveals that a large proportion of vitamin B12- and D- tests is ordered without a clear indication justifying their use. The total volumes of vitamin B12- and D-tests have increased over the years and show no inclination of declining. Based on publicly available fares, we estimates that roughly 3.8 million euro has been spent on non-indicated vitamin B12- and D-tests in 2019 by Dutch hospital clinicians alone. [41] This estimate of the potential savings, however, is very rough, while it only accounts for the cost price of a vitamin B12- or D-determination. The observed incidence rates of non-indicated vitamin B12- and D-testing, however, suggest that there is ample opportunity to reduce vitamin testing among Dutch clinicians. Especially since non-indicated vitamin B12- and D-tests are often ordered for similar diagnoses, and a positive correlation exists between the proportions of non-indicated testing within the same hospitals. We know that there are effective interventions to reduce inappropriate vitamin testing among GPs. A study among Dutch GPs showed that providing both education and feedback successfully reduced the amount of vitamin tests ordered with 20-25%. [42] Similar interventions might therefore also be effective among hospital clinicians to reduce (non-indicated) vitamin testing. Alternatively, more emphasis could be placed on the institution of fortification and supplementation guidelines to achieve adequate vitamin B12 and D intake among the Dutch population. Especially since, the implementation of such guidelines in

have shown to positively affect vitamin status among the population rendering vitamin testing obsolete in most cases. [43-45] Future research could focus on further examination of patient and physicians' characteristics associated with non-indicated vitamin B12- and D-testing. Unfortunately, information regarding the requesting physician (age, sex etc.) of non-indicated tests was not available to us in our study. Insight into physician characteristics associated with non-indicated testing could aid in the design of interventions aiming to address the problem of non-indicated vitamin testing.

Conclusion

Our research shows that the number of vitamin B12-tests slightly increased over the examined time period, while the number of vitamin D tests substantially increased among hospital clinicians. Throughout the examined period the proportion of B12- and D-tests without clear indication remained high, and are substantially higher compared to similar (international) assessments. The observed difference in assessment outcome can potentially be explained by differences in methods and definitions used to identify and define non-indicated vitamin B12- and D-tests (e.g. the use of associated diagnose codes instead of test results or proxy's such as testing interval). We also observed the presence of moderate hospital variation, but that this variation could not be explained by the included patient and hospital characteristics age, sex, SES and hospital size. Hospitals hardly differ in the task they have to undertake: bring down the number of non-indicated B12- and D-tests.

For peer review only

Additional information requested at submission

Financial disclosure statement

This work was supported by ZonMw, the Dutch organization for Health Research and Development (grant number 839205002). The funder had no role in the design and conduct of the study.

Conflict of interest interests

None to declare.

Acknowledgements:

The authors would like to thank Prof. dr. P. Lips, Professor Emeritus, APH - Aging & Later Life, Professor Emeritus of Internal medicine, Visiting Fellow Internal medicine from the Amsterdam UMC, dr. L. de Boer and dr. A. Dittrich for their critical review and contributions to the paper.

Related manuscripts

The authors indicate that they do not have a related or duplicate manuscript under consideration (or accepted) for publication elsewhere.

Ethics Approval Statement

The protocol for this research was approved by the Research Ethics Committee of the Radboud University Medical Centre (dossier number 2020-6767). Informed consent of individual patients was not required as anonymised information was obtained from medical records.

Data availability statement

The source data is not freely available. However the data used to conduct our analysis are available upon reasonable request after consultation with the data supplier.

For peer review only

Author contributions:

Conceptualization: Müskens, Kool, van Dulmen, Westert.

Data Curation: Zaal

Formal Analysis: Müskens, Atsma

Funding Acquisition: Kool, van Dulmen

Investigation: Müskens, Kool, van Dulmen, Muller

Methodology: Müskens, Atsma.

Project Administration: Müskens

Resources: Zaal

Software: Müskens

Supervision: Kool, van Dulmen, Westert

Validation: Müskens, Kool, van Dulmen

Visualization: Müskens

Writing – original draft preparation: Müskens, Kool, van Dulmen

Writing – Review & Editing: Müskens, Kool, van Dulmen, Zaal, Muller, Atsma, Westert

For peer review only

References

1. Müskens JLJM, Kool RB, van Dulmen SA, Westert GP. Overuse of diagnostic testing in healthcare: a systematic review. BMJ Qual Saf. 2022;31(1):54-63.
2. Brownlee S, Chalkidou K, Doust J, Elshaug AG, Glasziou P, Heath I, et al. Evidence for overuse of medical services around the world. Lancet. 2017;390(10090):156-68.
3. Colla CH, Mainor AJ, Hargreaves C, Sequist T, Morden N. Interventions Aimed at Reducing Use of Low-Value Health Services: A Systematic Review. Med Care Res Rev. 2017;74(5):507-50.
4. Scott IA, Duckett SJ. In search of professional consensus in defining and reducing low-value care. Med J Aust. 2015;203(4):179-81.
5. Essig S, Merlo C, Reich O, Trottmann M. Potentially inappropriate testing for vitamin D deficiency: a cross-sectional study in Switzerland. BMC Health Services Research. 2020;20(1):1097.
6. Crowe FL, Jolly K, MacArthur C, Manaseki-Holland S, Gittoes N, Hewison M, et al. Trends in the incidence of testing for vitamin D deficiency in primary care in the UK: a retrospective analysis of The Health Improvement Network (THIN), 2005–2015. BMJ Open. 2019;9(6):e028355.
7. Lanzoni M, Fornili M, Felicetta I, Maiavacca R, Biganzoli E, Castaldi S. Three-year analysis of repeated laboratory tests for the markers total cholesterol, ferritin, vitamin D, vitamin B(12) , and folate, in a large research and teaching hospital in Italy. J Eval Clin Pract. 2017;23(3):654-61.
8. O'Sullivan JW, Stevens S, Hobbs FDR, Salisbury C, Little P, Goldacre B, et al. Temporal trends in use of tests in UK primary care, 2000-15: retrospective analysis of 250 million tests. Bmj. 2018;363:k4666.
9. American Society for Clinical Pathology. Thirty Five Things Physicians and Patients Should Question 2021 [Available from: https://www.choosingwisely.org/clinician-lists/ascp-do-not-order-red-blood-cell-folate-levels/].
10. Richtlijnen database [Internet]. Federatie Medisch Specialisten,. 2022 [cited 12-12-2022]. Available from: https://richtlijnendatabase.nl/.
11. Dutch College of Clinical Chemistry and Laboratory Medicine & College of medical immunologists. Verstandige keuzes bij het aanvragen en interpreteren van laboratoriumdiagnostiek. 2015.
12. Canadian Association of Pathologists. Choosingwisely Canada Pathology Recommendations,. 2021.
13. Nederlands Huisartsen Genootschap. Laboratoriumdiagnostiek Vitamine B12-deficiëntie (LESA): NHG; 2018 [Available from: https://www.nhg.org/themas/publicaties/laboratoriumdiagnostiek-vitamine-b12-volledige-tekst.
14. Nederlands Huisartsen Genootschap. Vitamine D-deficiëntie (LESA Laboratoriumdiagnostiek): NHG; 2018 [Available from: https://www.nhg.org/themas/publicaties/vitamine-d-deficientie-samenvatting?tmp-no-mobile=1].
15. Chami N, Simons JE, Sweetman A, Don-Wauchope AC. Rates of inappropriate laboratory test utilization in Ontario. Clin Biochem. 2017;50(15):822-7.
16. Morgen EK, Naugler C. Inappropriate repeats of six common tests in a Canadian city: a population cohort study within a laboratory informatics framework. Am J Clin Pathol. 2015;144(5):704-12.
17. Gill P, Guo M, Lau CK, Naugler C. Vitamin B12 test volume data before and after the implementation of an educational province-wide intervention to reduce redundant testing in Alberta. Data Brief. 2019;27:104785.
18. Tamburrano A, Vallone D, Carrozza C, Urbani A, Sanguinetti M, Nicolotti N, et al. Evaluation and cost estimation of laboratory test overuse in 43 commonly ordered parameters through a

For peer review only

Computerized Clinical Decision Support System (CCDSS) in a large university hospital. PLoS One. 2020;15(8):e0237159.

19. Naugler C, Hemmelgarn B, Quan H, Clement F, Sajobi T, Thomas R, et al. Implementation of an intervention to reduce population-based screening for vitamin D deficiency: a cross-sectional study. CMAJ Open. 2017;5(1):E36-e9.

20. Colla CH, Morden NE, Sequist TD, Schpero WL, Rosenthal MB. Choosing wisely: prevalence and correlates of low-value health care services in the United States. J Gen Intern Med. 2015;30(2):221-8.

21. Mafi JN, Reid RO, Baseman LH, Hickey S, Totten M, Agniel D, et al. Trends in Low-Value Health Service Use and Spending in the US Medicare Fee-for-Service Program, 2014-2018. JAMA Network Open. 2021;4(2):e2037328-e.

22. Zorginstituut Nederland. Screeningsrapport Endocriene Ziekten; voedings- en stofwisselingstoornissen,. Zorginstituut Nederland; 2018.

23. Gonzalez-Chica D, Stocks N. Changes to the frequency and appropriateness of vitamin D testing after the introduction of new Medicare criteria for rebates in Australian general practice: evidence from 1.5 million patients in the NPS MedicineInsight database. BMJ Open. 2019;9(3):e024797.

24. Dutch Hospital Data. Landelijke Basisregistratie Zorg (LBZ). In: Data DH, editor.

25. The Netherlands Institute for Social Research. Statusscores 2017. Netherlands: The Netherlands Institute for Social Research (SCP); 2017.

26. Chalmers K, Pearson SA, Elshaug AG. Quantifying low-value care: a patient-centric versus service-centric lens. BMJ Qual Saf. 2017;26(10):855-8.

27. McMaughan DJ, Oloruntoba O, Smith ML. Socioeconomic Status and Access to Healthcare: Interrelated Drivers for Healthy Aging. Front Public Health. 2020;8:231.

28. Arpey NC, Gaglioti AH, Rosenbaum ME. How Socioeconomic Status Affects Patient Perceptions of Health Care: A Qualitative Study. J Prim Care Community Health. 2017;8(3):169-75.

29. Asch SM, Kerr EA, Keesey J, Adams JL, Setodji CM, Malik S, McGlynn EA. Who is at greatest risk for receiving poor-quality health care? N Engl J Med. 2006;354(11):1147-56.

30. David JL, Kaplan HB. Gender, social roles and health care utilization. Applied Behavioral Science Review. 1995;3(1):39-64.

31. Bertakis KD, Azari R, Helms LJ, Callahan EJ, Robbins JA. Gender differences in the utilization of health care services. J Fam Pract. 2000;49(2):147-52.

32. Segal JB, Sen AP, Glanzberg-Krainin E, Hutfless S. Factors Associated With Overuse of Health Care Within US Health Systems: A Cross-sectional Analysis of Medicare Beneficiaries From 2016 to 2018. JAMA Health Forum. 2022;3(1):e214543-e.

33. Snijders TAB, Bosker RJ. Multilevel Analysis: An Introduction to Basic and Advanced Multilevel Modeling: SAGE Publications; 2011.

34. Holick MF. Vitamin D deficiency. N Engl J Med. 2007;357(3):266-81.

35. Green R, Miller JW. Chapter Fifteen - Vitamin B12 deficiency. In: Litwack G, editor. Vitamins and Hormones. 119: Academic Press; 2022. p. 405-39.

36. Rebelos E, Tentolouris N, Jude E. The Role of Vitamin D in Health and Disease: A Narrative Review on the Mechanisms Linking Vitamin D with Disease and the Effects of Supplementation. Drugs. 2023;83(8):665-85.

37. Halczuk K, Kaźmierczak-Barańska J, Karwowski BT, Karmańska A, Cieślak M. Vitamin B12 — Multifaceted In Vivo Functions and In Vitro Applications. Nutrients. 2023;15(12):2734.

38. Knopman DS, DeKosky ST, Cummings JL, Chui H, Corey-Bloom J, Relkin N, et al. Practice parameter: diagnosis of dementia (an evidence-based review). Report of the Quality Standards Subcommittee of the American Academy of Neurology. Neurology. 2001;56(9):1143-53.

39. Marcotte LM, Schuttner L, Liao JM. Measuring low-value care: learning from the US experience measuring quality. BMJ Qual Saf. 2020;29(2):154-6.

40. Statistics Netherlands (CBS). [Zorginstellingen; kerngetallen]. 2019.

For peer review only

41. Diagnostiek voor u. Fares 2022 - 2023 2023 [Available from: https://diagnostiekvooru.nl/tarieven].

42. Vugt SV, de Schepper E, van Delft S, Zuithoff N, de Wit N, Bindels P. Effectiveness of professional and patient-oriented strategies in reducing vitamin D and B12 test ordering in primary care: a cluster randomised intervention study. BJGP Open. 2021;5(6).

43. Itkonen ST, Andersen R, Björk AK, Brugård Konde Å, Eneroth H, Erkkola M, et al. Vitamin D status and current policies to achieve adequate vitamin D intake in the Nordic countries. Scand J Public Health. 2021;49(6):616-27.

44. Jääskeläinen T, Itkonen ST, Lundqvist A, Erkkola M, Koskela T, Lakkala K, et al. The positive impact of general vitamin D food fortification policy on vitamin D status in a representative adult Finnish population: evidence from an 11-y follow-up based on standardized 25-hydroxyvitamin D data. Am J Clin Nutr. 2017;105(6):1512-20.

45. Das JK, Salam RA, Mahmood SB, Moin A, Kumar R, Mukhtar K, et al. Food fortification with multiple micronutrients: impact on health outcomes in general population. Cochrane Database Syst Rev. 2019;12(12):Cd011400.

For peer review only

470 *Table 3: overview of the study population characteristics of the used population over the entire period examined (2015 – 2019). Abbreviations; Socioeconomic status (SES), Standard deviation*
471 *(SD).*

472

General info regarding the used population between 2015 - 2019	Number or proportion	Min	Max	Median	Interquartile range
Total number of unique patients	9,214,425	N.A.	N.A.	N.A.	N.A.
Gender (% female)	64.50%	N.A.	N.A.	N.A.	N.A.
Average number of patients among the hospitals (+SD)	126,722 (± 169,110)	37	1,244,526	126,722	119,601.8
Average number of unique patients among the hospitals (+SD)	47,561 (± 54317.5)	32	415,673	36,561	38,590
Average age of the patients included (+SD)	47.91 (± 14.60)	18	70	N.A.	N.A.
Average SES category of the patients included (+SD)	2.86 (± 1.43)	1	6	N.A.	N.A.
Average no. of outpatient visits among the hospitals included	1,721,337 (± 819,148)	470,117	4,659,172	N.A.	N.A.

For peer review only

Table 4: Overview of vitamin B12 and D tests performed among hospital clinicians over the period examined (2015 – 2019). Abbreviations: Diagnosis Treatment Combination (DTC)

**Year**	**2015**	**2016**	**2017**	**2018**	**2019**
**No. of hospitals included**	63	64	66	69	68
**Total no. B12-tests**	275,032	265,546	279,524	279,957	280,522
**Total no. B12-tests in hospital with a registered DTC code**	118,126	163,244	188,063	208,012	213,308
(%) of B12-test with a registered DTC without clear indication	79.0%	78.0%	78.2%	78.3%	78.1%
**Total no. of patients with (at least) one vitamin B12-test**	233,541	226,999	239,033	240.063	239.351
**Total no. of patients with (at least) one vitamin B12-test with a registered DTC code**	103,540	141,507	162,900	178,933	183,204
**Total no. of patients with (at least) one non-indicated vitamin B12-test (with a registered DTC code)**	83,549	111,986	128,872	141,914	145,039
(%) patients that received at least one B12-test that was considered non-indicated	80.7%	79.1%	79.1%	79.3%	79.2%
**No. of hospitals included**	62	61	63	65	65
**Total no. D tests**	300,013	300,427	322,703	323,074	336,736
**Total no. D tests in hospital with a registered DTC code**	121,892	179,271	208,361	230,620	242,040
(%) of D-test with a registered DTC without clear indication	82.5%	82.9%	82.7%	82.5%	82.0%
**Total no. of patients with (at least) one vitamin D-test**	244,834	247,186	264,811	265,362	272,380
**Total no. of patients with (at least) one vitamin D-test with a registered DTC code**	101,932	148,660	172,342	189,423	196,452
**Total no. of patients with (at least) one non-indicated vitamin D-test (with a registered DTC code)**	84,414	124,162	143,408	157,431	162,345
(%) patients that received at least one B12-test that was considered non-indicated	82.8%	83.5%	83.2%	83.1%	82.6%

For peer review only

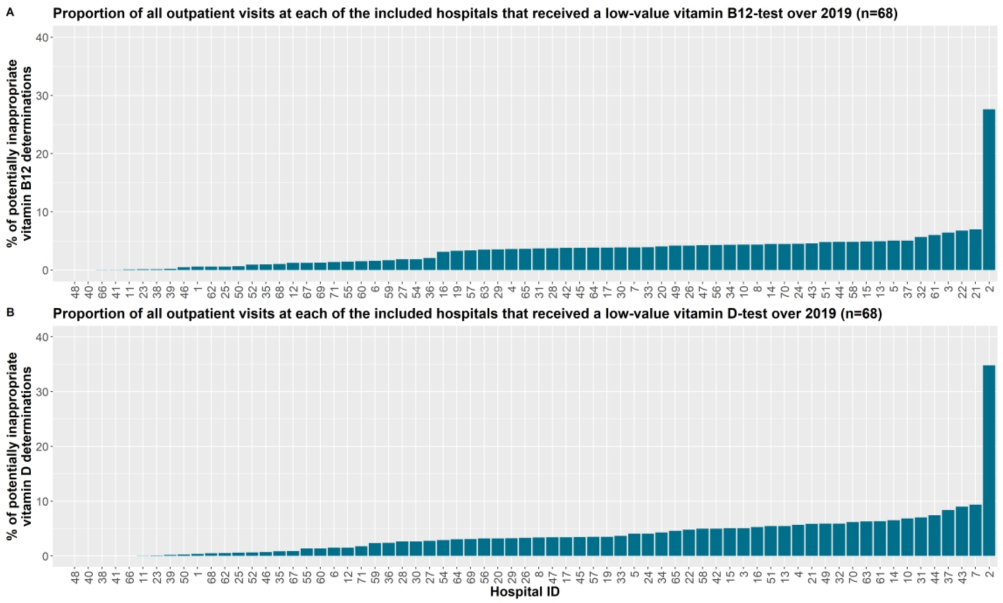

Figure 1: proportion of all outpatient visits at each of the included hospitals that received a low-value vitamin (A) B12- or (B) D- test over 2019 (n=68).

423x254mm (300 x 300 DPI)

Supplementary file S1: Included International classification of disease (ICD10) codes and subsequent diagnose-treatment combination (DTC) codes.

The table below contains the codes which were used to distinguish indicated from non-indicated vitamin B12- and D-tests. In case of vitamin D-testing, tests were considered to be appropriate in patients older than 50 with a fracture. We therefore have also provided a table of the fractures that were included.

Vitamin B12			
**ICD-10 code**	**Description**	**DTC code**	**DTC-Diagnosis description**
D51	**Vitamin B_{12} deficiency anaemia**<br>*Excl.: Vitamin B_{12} deficiency (E53.8)*	- 313 – 701<br>- 313 – 702<br>- 313 – 709<br>- 316 – 6001<br>- 316 – 6003<br>- 335 – 232	- Iron-deficiency anemia (not specified)<br>- Pernicious anemia<br>- Other erytrocital deviations (not specified)<br>- Anemia, Iron-deficiency<br>- Anemia, remaining<br>- Anemia
D52	**Folate deficiency anaemia**		
D53	**Other nutritional anaemias**<br>*Incl.: megaloblastic anaemia unresponsive to vitamin B_{12} or folate therapy*		
E53.8	**Deficiency of other specified B group vitamins**<br>*Deficiency:*<br>• *Biotin*<br>• *Cyanocobalamin*<br>• *Folate*<br>• *Folic acid*<br>• *Panthothenic acid*<br>• *Vitamin B12*	- 318 – 207<br>- 330 – 301	- (mal)nutrition<br>- Vitamin-deficiencies
E56.8	**Deficiency of other vitamins**		
E56.9	**Vitamin deficiency, unspecified**		
G63.4	**Polyneuropathy in nutritional deficiency**	- 327 – 413<br>- 330 – 312<br>- 330 – 812<br>- 335 – 251	- Pheripheral nervedamage, nerve disorder<br>- Neurological complication systemic disease<br>- Polyneuropathy, other<br>- Diseases of the nerve system and senses
R20.2	**Paraesthesia of skin**<br>*Excl.: acroparaesthesia (I73.8)*		
R27.0	**Ataxia, unspecified**		
K29.4	**Chronic atrophic gastritis**	- 313 – 916<br>- 318 – 401	- Erosive gastritis and duodenitis<br>- Gastritis, miscellaneous
K50	**Crohn disease [regional enteritis]**<br>*Incl.: granulomatous enteritis*<br>*Excl.: ulcerative colitis (K51.-)*	- 303 – 326<br>- 313 – 922<br>- 318 – 601<br>- 316 - 3314	- Crohn disease (enteritis regionalis)<br>- Crohn disease<br>- Morbus Crohn<br>- Inflammatory bowldisease (colitis ulcerosa/Morbus crohn)
K52	**Other noninfective gastroenteritis and colitis**	- 313 – 932<br>- 313 – 938	- Chronic diarrhoea without infection<br>- Radiation enteritis
K90	**Intestinal malabsorption**<br>*Excl."following gastrointestinal surgery (K91.2)*	- 318 - 409	- Coeliac disease
Z98.0	**Internal bypass and anastomosis status**	- 303 – 341<br>- 303 – 342	- Morbid obesity BMI < 45<br>- Morbid obesity BMI > 45

For peer review only

Vitamin D			
**ICD-10 code**	**Description**	**DTC code**	**DTC-Diagnosis description**
E20<br>E21	**Hypoparathyroidism**<br>**Hyperparathyroidism and other disorders of parathyroid gland**	- 307 – E00<br>- 307 – E11<br>- 313 – 231<br>- 313 – 232<br>- 316 – 7110<br>- 316 – 7199<br>- 362 – 304	- Emergency consult Endocrinology<br>- Endocrinology<br>- Hyperparathyroidism<br>- Hypoparathyroidism<br>- Small body length/deviant growth curve<br>- Endocrinology, not specified<br>- Parathyroid glands
E83.5	**Disorders of calcium metabolism**	- 313 – 239<br>- 313 – 299<br>- 316 - 7503<br>- 330 - 399	- Calcium metabolic disease, unspecified<br>- Endocrine and metabolic diseases, unspecified<br>- Metabolic disease<br>- Deficiencies, metabolic, nutritional, unspecified
E84.9	**Cystic fibrosis, unspecified**	- 316 – 3205<br>- 322 - 1403	- Cystic fibrosis<br>- Cystic fibrosis
E55	**Vitamin D deficiency**<br>*Excl.: adult osteomalacia (M83.-), osteoporosis (M80-M81), sequelae of rickets (E64.3)*	- 305 – 1062<br>- 335 – 221<br>- 318 – 207<br>- 335 – 224	- Rachitis and/or vitamin D resistance osteomalacia<br>- Endocrine and nutrition disorders<br>- (mal)nutrition<br>- Calory/protein malnutrition
M83	**Adult osteomalacia**<br>*Excl.: osteomalacia:*<br>• *infantile and juvenile (E55.0)*<br>• *vitamin-D-resistant (E83.3)*<br>*Renal osteodystrophy (N25.0)*<br>*Rickets (active) (E55.0)*<br>*Rickets (active)*<br>• *sequelae (E64.3)*<br>*vitamin-D-resistant (E83.3)*		
E83.3	**Disorders of phosphorus metabolism and phosphates**<br>Acid phosphatase deficiency<br>Familial hypophosphataemia<br>Hypophosphatasia<br>Vitamin-D-resistant:<br>• Osteomalacia<br>• rickets<br>*Excl.: adult osteomalacia (M83.-), osteoporosis (M80-M81)*	- 316 – 3328<br>- 316 – 7503	- Food related problems/disorders<br>- Metabolic disease
K50	**Crohn disease [regional enteritis]**<br>*Incl.: granulomatous enteritis*<br>*Excl.: ulcerative colitis (K51.-)*	- 303 – 326<br>- 313 – 922<br>- 318 – 601	- Crohn disease (enteritis regionalis)<br>- Crohn disease<br>- Morbus Crohn
K90	**Intestinal malabsorption**<br>*Excl.”following gastrointestinal surgery (K91.2)*	- 313 – 920<br>- 318 – 409	- Coeliac disease / malabsorption<br>- Coeliac disease
M80	**Osteoporosis with pathological fracture**<br>*Incl.: osteoporotic vertebral collapse and wedging*<br>*Excl.: collapsed vertebra NOS (M48.5), pathological fracture NOS (M84.4), wedging of vertebra NOS (M48.5)*	- 305 – 1395<br>- 316 – 5105	- Osteoperotic collapse<br>- Pathological fractures

For peer review only - http://bmjopen.bmj.com/site/about/guidelines.xhtml

M81	**Osteoporosis without pathological fracture**<br>*Excl.: osteoporosis with pathological fracture (M80.-)*	- 313 – 233	- Osteoperosis, osteomalacia
M82	**Osteoporosis in diseases classified elsewhere**		
N25.0	**Renal osteodystrophy**<br>*Azotaemic osteodystrophy*<br>*Phosphate-losing tubular disorders*<br>*Renal:*<br>• *rickets*<br>• *short stature*	- 313 – 399<br>- 313 – 325	- Renal diseases, miscellaneous<br>- Chronic renal disease (eGFR <30 ml/min)

Additional DTC codes added after checking the resulting indications:

D	0316	3304	Celiac disease	6509
D	0316	4006	Chronic renal failure	1245
D	0318	753	Chronic pancreatitis	3415
B12	0316	3304	Celiac disease	6233
B12	0313	772	Polycythemia vera, essential trombocytosis	5434
B12	0313	763	Myelodysplastic syndrome no specified	4843
B12	0313	920	Celiac disease/malabsorption	4206
B12	0313	773	Chronic myelogenous leukemia (CMMoL)	312

For peer review only

Bone fractures above 50 included in our analysis, that justify the ordering of a vitamin D-test.

DTC specialism code	DTC diagnose code	Fracture description.
0305	3011	Radius head
0305	3014	Carpus
0305	3029	Metatarsal bones
0303	207	Humerus proximal and shaft
0305	3024	Tibia (with or without fibula, excluding ankle)
0303	210	Radius head
0304	451	Fracture of distal radius surgical
0303	219	Femur, remaining
0303	221	Tibial plateau
0303	222	Tibia (± fibula, excluding ankle)
0305	3023	Tibial plateau
0303	217	Pelvis / sacrum
0303	214	Metacarpal bones
0305	3008	Humerus proximal and shaft
0303	224	Ankle
0305	3026	Calcaneus
0305	3030	Phalanges of the feet
0308	2301	Trauma vertebral column: conservative treatment
0303	241	Talus
0305	3020	Femur, remaining
0303	237	Tarsus
0303	215	Phalanges of the hand
0305	3027	Talus
0304	420	Fracture / luxation carpalia conservative
0305	3017	Pelvis
0305	3005	Vertebral column with paraplegia
0303	220	Patella
0303	238	Metatarsal bones
0305	3004	Vertebral column
0305	3009	Distal humerus/(epi)condyl(len)
0305	3015	Metacarpal bones
0305	3022	Fibula
0305	3025	Ankle
0303	239	Phalanges of the feet
0303	209	Olecranon
0303	213	Carpus
0305	3013	Wrist
0308	2311	Trauma vertebral column: Surgical intervention including instrumentation c.q. spinal fusion
0304	450	Distal radius facture conservative
0304	8418	Traumatic Vertebral compression fracture
0303	211	Forearm, no further specification
0305	3021	Patella
0304	421	Fracture / luxation carpalia operative
0303	212	Wrist
0303	208	Distal humerus / (epi)condyl(en)
0303	203	Vertebral column
0303	204	Vertebral column with spinal lesion
0308	2305	Treatment of trauma to the vertebral column with external fixation or traction
0305	3010	Olecranon

For peer review only

0305	3028	Tarsus
0303	236	Calcaneus
0305	3012	Forearm
0305	3016	Phalanges of the hand
0303	218	Fracture of neck of femur (disorder)
0305	3019	Femur proximaal (+ collum)

For peer review only

Supplementary file S2: overview calculated generalized variance inflation factors (GVIF).

Generalized Variation Inflation Factors (GVIF) calculated for the models made using DHD data.

Year	Vitamin test	Variable	GVIF	GVIF^(1/(2*DF))
2015	Vitamin B12	*Gender*	1.038	1.019
		Age category	1.040	1.010
		Socio-economic status category	1.017	1.0021
		Hospital size	1.016	1.0041
	Vitamin D	*Gender*	1.013	1.0065
		Age category	1.016	1.0039
		Socio-economic status category	1.029	1.0035
		Hospital size	1.028	1.0069
2016	Vitamin B12	*Gender*	1.036	1.018
		Age category	1.037	1.0092
		Socio-economic status category	1.01	1.0017
		Hospital size	1.013	1.0033
	Vitamin D	*Gender*	1.012	1.0060
		Age category	1.013	1.0033
		Socio-economic status category	1.019	1.0024
		Hospital size	1.020	1.0050
2017	Vitamin B12	*Gender*	1.035	1.017
		Age category	1.036	1.0089
		Socio-economic status category	1.017	1.0021
		Hospital size	1.017	1.0043
	Vitamin D	*Gender*	1.013	1.0064
		Age category	1.014	1.0037
		Socio-economic status category	1.023	1.0028
		Hospital size	1.023	1.0057
2018	Vitamin B12	*Gender*	1.038	1.019
		Age category	1.040	1.0097
		Socio-economic status category	1.025	1.0031
		Hospital size	1.024	1.0060
	Vitamin D	*Gender*	1.016	1.0078
		Age category	1.018	1.0044
		Socio-economic status category	1.029	1.0036
		Hospital size	1.030	1.0073
2019	Vitamin B12	*Gender*	1.035	1.0175
		Age category	1.037	1.0091
		Socio-economic status category	1.035	1.0043
		Hospital size	1.035	1.0085
	Vitamin D	*Gender*	1.015	1.0072
		Age category	1.017	1.0041
		Socio-economic status category	1.040	1.0049
		Hospital size	1.040	1.0098

For peer review only

Supplementary file S3: Extended table 1; distribution of vitamin B12- and D-testing across the sex, age and SES categories.

Table 1: extended table 1 for vitamin B12.

1. Vitamin B12																
		**2015**			**2016**			**2017**			**2018**			**2019**		
	No. of hospitals included	63			64			66			69			68		
	Total no. of unique patients with (at least) one vitamin B12 test	233,541			226,999			239,033			240,063			239,351		
	Total no. of unique patients with (at least) one vitamin B12 test with an associated DBC code	103,540			141,507			162,900			178,933			183,204		
	Total no. of unique patients with (at least) one non-indicated vitamin B12 test	83,549			111,986			128,872			141,914			145,039		
		**Total**	**Without (clear) indication**	**% without (clear indication**	**Total**	**Without (clear) indication**	**% without (clear indication**	**Total**	**Without (clear) indication**	**% without (clear indication**	**Total**	**Without (clear) indication**	**% without (clear indication**	**Total**	**Without (clear) indication**	**% without (clear indication**
**Male**	**No. of male patients receiving at least one vitamin-B12 test**	38,308	31,731	82.8	52,360	42,758	81.7	60,511	49,222	81.3	66,724	54,567	81.8	67,869	55,148	81.3
Age cat.	**18-29**	3,152	2,461	78.1	4,473	3,421	76.5	5,262	4,094	77.8	5,921	4,579	77.3	6,468	4,886	75.5
	**30-49**	9,785	7,967	81.4	13,346	10,598	79.4	15,247	12,119	79.5	16,397	13,175	80.4	16,864	13,572	80.5
	**50-69**	25,371	21,303	84.0	34,541	28,739	83.2	40,002	33,009	82.5	44,406	36,813	82.9	44,537	36,690	82.4
Neighbou	***Lowest socio-economic status****	8,070	6,693	82.9	11,346	9,271	81.7	13,512	11,082	82.0	14,632	12,008	82.1	15,099	12,346	81.8
**SES cat.**	***Socio-economic status below average***	8,484	7,022	82.8	11,347	9,236	81.4	13,601	11,078	81.4	14,686	12,001	81.7	14,962	12,116	81.0
	Average socio-economic status	7,790	6,472	83.1	10,372	8,472	81.7	11,669	9,447	81.0	13,296	10,908	82.0	13,086	10,593	80.9
	Socio-economic status above average	8,067	6,698	83.0	10,481	8,576	81.8	11,738	9,480	80.8	12,914	10,527	81.5	13,150	10,687	81.3
	Highest socio-economic status	5,639	4,625	82.0	8,426	6,860	81.4	9,564	7,766	81.2	10,666	8,665	81.2	10,985	8,915	81.2
	Missing	258	221	85.7	388	343	88.4	427	369	86.4	530	458	86.4	587	491	83.6
**Female**	**No. of female patients receiving at least one vitamin-B12 test**	65,232	51,818	79.4	89,147	69,228	77.7	102,389	79,650	77.8	112,209	87,347	77.8	115,335	89,891	77.9
Age cat.	**18-29**	9,138	7,155	78.3	12,781	9,751	76.3	14,863	11,415	76.8	16,799	13,011	77.5	17,371	13,487	77.6
	**30-49**	24,905	18,991	76.3	34,030	25,313	74.4	38,672	28,996	75.0	42,186	31,649	75.0	43,276	32,826	75.9
	**50-69**	31,189	25,672	82.3	42,336	34,164	80.7	48,854	39,239	80.3	53,224	42,687	80.2	54,688	43,578	79.7
Neighbou	***Lowest socio-economic status****	14,351	11,223	78.2	19,935	15,267	76.6	23,709	18,370	77.5	25,526	19,832	77.7	26,005	20,190	77.6
**SES cat.**	***Socio-economic status below average***	14,520	11,513	79.3	19,865	15,328	77.2	23,072	17,820	77.2	25,157	19,555	77.7	25,860	20,112	77.8
	Average socio-economic status	13,035	10,453	80.2	17,656	13,737	77.8	19,669	15,213	77.3	21,936	17,000	77.5	22,334	17,366	77.8
	Socio-economic status above average	13,122	10,459	79.7	17,006	13,245	77.9	19,098	14,899	78.0	21,366	16,625	77.8	21,984	17,079	77.7
	Highest socio-economic status	9,850	7,871	79.9	14,226	11,277	79.3	16,294	12,914	79.3	17,601	13,813	78.5	18,532	14,643	79.0
	Missing	354	299	84.5	459	374	81.5	547	434	79.3	623	522	83.8	620	501	80.8

For peer review only

Table 1: extended table 1 for vitamin D.

	c															
		**2015**			**2016**			**2017**			**2018**			**2019**		
	No. of hospitals included	62			61			63			65			65		
	Total no. of patients with (at least) one vitamin D test	244,834			247,186			264,811			265,362			272,380		
	Total no. of patients with (at least) one vitamin D test with an associated DBC code	101,932			148,660			172,342			189,423			196,452		
	Total no. of patients with (at least) one non-indicated vitamin D test	84,414			124,162			143,408			157,431			162,345		
		**Total**	**Without (clear) indication**	**% without (clear indication**	**Total**	**Without (clear) indication**	**% without (clear indication**	**Total**	**Without (clear) indication**	**% without (clear indication**	**Total**	**Without (clear) indication**	**% without (clear indication**	**Total**	**Without (clear) indication**	**% without (clear indication**
**Male**	**No. of male patients receiving at least one vitamin-D test**	31,660	26,363	83.3	47,794	40,211	84.1	56,211	47,313	84.2	61,749	51,845	84.0	63,295	52,656	83.2
Age cat.	**18-29**	2,689	2,220	82.6	4,201	3,426	81.6	5,185	4,179	80.6	5,992	4,779	79.8	6,529	5,052	77.4
	**30-49**	8,684	7,658	88.2	13,086	11,536	88.2	15,614	13,701	87.7	16,964	14,750	86.9	17,483	15,123	86.5
	**50-69**	20,287	16,485	81.3	30,507	25,249	82.8	35,412	29,433	83.1	38,793	32,316	83.3	39,283	32,481	82.7
**Neighbou**	***Lowest socio-economic status****	6,358	5,326	83.8	10,349	8,813	85.2	12,430	10,559	84.9	13,295	11,272	84.8	13,578	11,323	83.4
**SES cat.**	***Socio-economic status below average***	6,758	5,584	82.6	9,964	8,342	83.7	12,199	10,201	83.6	13,249	11,079	83.6	13,818	11,496	83.2
	Average socio-economic status	6,331	5,233	82.7	9,311	7,755	83.3	10,677	8,851	82.9	12,292	10,249	83.4	12,287	10,092	82.1
	Socio-economic status above average	6,855	5,746	83.8	9,476	7,955	83.9	10,833	9,090	83.9	11,678	9,739	83.4	12,134	10,134	83.5
	Highest socio-economic status	5,164	4,309	83.4	8,405	7,092	84.4	9,735	8,315	85.4	10,825	9,151	84.5	11,025	9,238	83.8
	Missing	194	165	85.1	289	254	87.9	337	297	88.1	410	355	86.6	453	373	82.3
**Female**	**No. of female patients receiving at least one vitamin-D test**	70,272	58,051	82.6	100,866	83,951	83.2	116,131	96,095	82.7	127,674	105,586	82.7	133,157	109,689	82.4
Age cat.	**18-29**	8,105	7,198	88.8	11,485	10,030	87.3	14,038	12,120	86.3	16,091	13,864	86.2	17,271	14,855	86.0
	**30-49**	25,045	22,802	91.0	35,377	32,119	90.8	40,982	37,028	90.4	45,932	41,407	90.1	47,884	42,896	89.6
	**50-69**	37,122	28,051	75.6	54,004	41,802	77.4	61,111	46,947	76.8	65,651	50,315	76.6	68,002	51,938	76.4
**Neighbou**	***Lowest socio-economic status****	15,024	12,710	84.6	22,104	18,873	85.4	26,254	22,286	84.9	28,219	23,903	84.7	29,182	24,613	84.3
**SES cat.**	***Socio-economic status below average***	15,087	12,344	81.8	21,509	17,770	82.6	25,087	20,641	82.3	27,646	22,868	82.7	28,971	23,760	82.0
	Average socio-economic status	13,987	11,419	81.6	19,561	16,032	82.0	22,374	18,184	81.3	24,947	20,169	80.8	25,582	20,690	80.9
	Socio-economic status above average	14,302	11,739	82.1	19,405	15,976	82.3	21,663	17,576	81.1	24,007	19,542	81.4	25,303	20,528	81.1
	Highest socio-economic status	11,581	9,608	83.0	17,823	14,923	83.7	20,248	17,011	84.0	22,235	18,618	83.7	23,500	19,606	83.4
	Missing	291	231	79.4	464	377	81.3	505	397	78.6	620	486	78.4	619	492	79.5

Supplementary file S4: top 20 diagnose codes associated with non-indicated vitamin B12- and D-testing.

Table 1: Top 20 of diagnose codes associated with non-indicated vitamin B12-tests.

No. of non-indicated vitamin B12-tests	DTC specialism code	DTC diagnose code	DTC specialism description	DTC diagnose description
31,055	313	3	Internal medicine	Analysis general malaise/fatigue without diagnosis
30,807	313	222	Internal medicine	Diabetes mellitus with secondary complications
26,591	313	283	Internal medicine	Adiposity (obesity)
25,268	318	602	Gastro-enterology	Ulcerative colitis
20,667	313	221	Internal medicine	Diabetes mellitus without secondary complications
20,118	307	Z41	Obstetrics and gynaecology	Miscellaneous counselling during pregnancy
18,747	318	205	Gastro-enterology	Irritable bowel syndrome, diverticulosis
12,136	313	211	Internal medicine	Hypothyroidism
11,765	330	531	Neurology	Multiple sclerosis
11,168	313	6	Internal medicine	Analysis stomach complaints without diagnosis
10,003	313	223	Internal medicine	Diabetes mellitus chronic pump therapy
8,358	318	203	Gastro-enterology	Blood loss Gastrointestinal tract, unknown cause
8,057	324	701	Rheumatology	Arthralgia and/or myalgia
7,822	313	4	Internal medicine	Analysis anorexia, emaciation without diagnosis
7,770	318	204	Gastro-enterology	Chronic stomach-ache
7,425	330	402	Neurology	Miscellaneous cognitive and memory impairments
6,841	313	311	Internal medicine	Hypertension
6,758	324	101	Rheumatology	Rheumatoid arthritis
6,688	313	324	Internal medicine	Chronic kidney insufficiency eGFR 30-60 ml/min
6,339	313	299	Internal medicine	Miscellaneous endocrine and metabolic conditions

For peer review only

Table 2: Top 20 of diagnose codes associated with non-indicated vitamin D-tests

**No. of non-indicated vitamin D-tests**	**DTC specialism code**	**DTC diagnose code**	**DTC specialism description**	**DTC diagnose description**
38,213	303	342	General surgery	Morbid obesity BMI >45
34,842	313	283	Internal medicine	Adiposity (obesity)
30,175	307	Z41	Obstetrics and gynaecology	Miscellaneous counselling during pregnancy
29,857	324	701	Rheumatology	Arthralgia and/or myalgia
25,072	318	602	Gastro-enterology	Ulcerative colitis
23,263	313	3	Internal medicine	Analysis general malaise/fatigue without diagnosis
21,723	313	222	Internal medicine	Diabetes mellitus with secondary complications
21,076	324	101	Rheumatology	Rheumatoid arthritis
19,013	313	811	Internal medicine	Malignancy mama
18,346	330	531	Neurology	Multiple sclerosis
17,208	313	324	Internal medicine	Chronic kidney insufficiency eGFR 30-60 ml/min
15,050	313	461	Internal medicine	HIV infection with treatment indication
12,718	303	341	General surgery	Morbid obesity BMI <45
12,354	313	76	Internal medicine	Recipient kidney transplant
12,155	324	707	Rheumatology	Fibromyalgia
12,025	313	221	Internal medicine	Diabetes mellitus without secondary complications
11,182	313	211	Internal medicine	Hypothyroidism
10,926	313	311	Internal medicine	Hypertension
9,960	322	1201	Respiratory medicine	Asthma
8,639	318	205	Gastro-enterology	Irritable bowel syndrome, diverticulosis

For peer review only

Supplementary file S5: Hospital variation; overview of Intraclass correlation coefficient across years (ICC) of both the casemix adjusted and unadjusted models for vitamin B12- and D-testing included in our analysis (2015 – 2019).

Table 3: Hospital variation; overview of Intraclass correlation coefficient across years (ICC) of both the casemix adjusted and unadjusted models for vitamin B12- and D-testing included in our analysis (2015 – 2019). Patient age, gender, socioeconomic status and an proxy for hospital size were included as casemix variables in the adjusted models.

	2015		2016		2017		2018		2019	
	Unadjusted	*Adjusted*	*Unadjusted*	*Adjusted*	*Unadjusted*	*Adjusted*	*Unadjusted*	*Adjusted*	*Unadjusted*	*Adjusted*
**Vitamin-B12**	0.083	0.072	0.094	0.085	0.088	0.081	0.089	0.079	0.095	0.082
**Vitamin-D**	0.101	0.095	0.096	0.083	0.085	0.081	0.095	0.090	0.099	0.095

For peer review only

Supplementary file S6: overview of the raw data and proportions of the total outpatient visits that received a vitamin B12- and D- test for each of the included hospitals over 2019.

Table 1: Proportion (%) of outpatient visits that received a vitamin B12-tests without a clear indication,

No,	Hospital ID	Total no, of outpatient visits recorded in 2019	Total no, of vitamin B12 tests without a clear indication	Proportion (%) of outpatient visits that received a vitamin B12 test without clear indication
1	48	362,887	2	0.00
2	40	580,678	8	0.00
3	66	127,984	52	0.04
4	41	576,063	263	0.05
5	11	345,279	407	0.12
6	23	568,860	716	0.13
7	38	289,822	400	0.14
8	39	565,276	1,094	0.19
9	46	358,602	1,693	0.47
10	1	344,543	1,953	0.57
11	62	227,934	1,324	0.58
12	25	165,640	985	0.59
13	50	436,838	2,808	0.64
14	52	508,460	4,723	0.93
15	35	156,620	1,485	0.95
16	68	133,631	1,387	1.04
17	12	494,601	6,081	1.23
18	67	153,812	1,915	1.25
19	69	318,615	4,021	1.26
20	71	408,355	5,680	1.39
21	55	194,373	2,792	1.44
22	60	421,743	6,422	1.52
23	6	384,801	6,032	1.57
24	59	395,402	6,608	1.67
25	27	389,827	7,214	1.85

For peer review only

26	54	383,123	7,109	1.86
27	36	363,166	7,477	2.06
28	16	163,322	5,133	3.14
29	19	131,553	4,361	3.32
30	57	280,541	9,441	3.37
31	63	427,252	15,000	3.51
32	29	216,550	7,672	3.54
33	4	315,289	11,366	3.60
34	65	237,265	8,670	3.65
35	31	309,391	11,457	3.70
36	28	113,439	4,239	3.74
37	42	268,758	10,220	3.80
38	45	384,873	14,745	3.83
39	64	342,101	13,120	3.84
40	17	155,415	6,008	3.87
41	30	370,550	14,413	3.89
42	7	384,199	14,944	3.89
43	33	606,974	23,806	3.92
44	20	147,262	5,997	4.07
45	49	259,604	10,903	4.20
46	26	182,212	7,660	4.20
47	47	185,195	7,875	4.25
48	56	192,410	8,244	4.28
49	34	429,614	18,618	4.33
50	10	276,454	12,102	4.38
51	8	223,140	9,775	4.38
52	14	567,264	25,389	4.48
53	70	253,998	11,381	4.48
54	24	654,329	29,546	4.52
55	43	536,979	24,622	4.59
56	51	443,539	21,314	4.81
57	44	356,220	17,236	4.84

For peer review only

58	58	154,585	7,521	4.87
59	15	165,151	8,097	4.90
60	13	466,598	23,040	4.94
61	5	401,794	20,260	5.04
62	37	171,709	8,665	5.05
63	32	337,152	19,183	5.69
64	61	174,166	10,466	6.01
65	3	113,329	7,276	6.42
66	22	251,812	17,093	6.79
67	21	186,579	13,004	6.97
68	2	379,408	104,750	27.61

For peer review only

Table 2: Proportion (%) of outpatient visits that received a vitamin D tests without a clear indication,

No,	Hospital ID	Total no, of outpatient visits recorded in 2019	Total no, of vitamin D determinations without a clear indication	Proportion (%) of outpatient visits that received a vitamin D determination without clear indication
1	48	362,887	0	0.00
2	40	580,678	0	0.00
3	38	289,822	0	0.00
4	41	576,063	576,063	0.02
5	66	127,984	127,984	0.02
6	11	345,279	345,279	0.06
7	23	568,860	568,860	0.08
8	39	565,276	565,276	0.20
9	50	436,838	436,838	0.28
10	1	344,543	344,543	0.38
11	68	133,631	133,631	0.48
12	62	227,934	227,934	0.54
13	25	165,640	165,640	0.59
14	52	508,460	508,460	0.64
15	46	358,602	358,602	0.71
16	35	156,620	156,620	0.84
17	67	153,812	153,812	0.89
18	55	194,373	194,373	1.35
19	60	421,743	421,743	1.35
20	6	384,801	384,801	1.49
21	12	494,601	494,601	1.52
22	71	408,355	408,355	1.75
23	59	395,402	395,402	2.36
24	36	363,166	363,166	2.39
25	28	113,439	113,439	2.64
26	30	370,550	370,550	2.65
27	27	389,827	389,827	2.75

For peer review only

28	54	383,123	383,123	2.88
29	64	342,101	342,101	3.04
30	69	318,615	318,615	3.07
31	56	192,410	192,410	3.20
32	20	147,262	147,262	3.20
33	29	216,550	216,550	3.23
34	26	182,212	182,212	3.30
35	8	223,140	223,140	3.39
36	47	185,195	185,195	3.41
37	17	155,415	155,415	3.41
38	45	384,873	384,873	3.45
39	57	280,541	280,541	3.50
40	19	131,553	131,553	3.50
41	33	606,974	606,974	3.67
42	5	401,794	401,794	4.05
43	24	654,329	654,329	4.06
44	34	429,614	429,614	4.30
45	65	237,265	237,265	4.58
46	22	251,812	251,812	4.80
47	58	154,585	154,585	4.97
48	42	268,758	268,758	5.00
49	15	165,151	165,151	5.07
50	3	113,329	113,329	5.07
51	16	163,322	163,322	5.27
52	51	443,539	443,539	5.44
53	13	466,598	466,598	5.46
54	4	315,289	315,289	5.68
55	21	186,579	186,579	5.84
56	49	259,604	259,604	5.91
57	32	337,152	337,152	5.91
58	70	253,998	253,998	6.17
59	63	427,252	427,252	6.31

For peer review only

60	61	174,166	174,166	6.33
61	14	567,264	567,264	6.50
62	10	276,454	276,454	6.83
63	31	309,391	309,391	7.04
64	44	356,220	356,220	7.42
65	37	171,709	171,709	8.37
66	43	536,979	536,979	9.00
67	7	384,199	384,199	9.37
68	2	379,408	379,408	34.79

For peer review only

Supplementary file S7: Density plots and Shapiro-Wilk normality test outcomes and the correlation test outcomes (with and without the outlierincluded)

Table 1: Normality testing outcomes

Variable	Shapiro-Wilk normality test	P-value (significance p<0.05)	Outcome
Rate of non-indicated vitamin-B12 tests	0.59	1.46e-12	Not normally distributed
Rate of non-indicated vitamin-D tests	0.58	1.19e -12	Not normally distributed

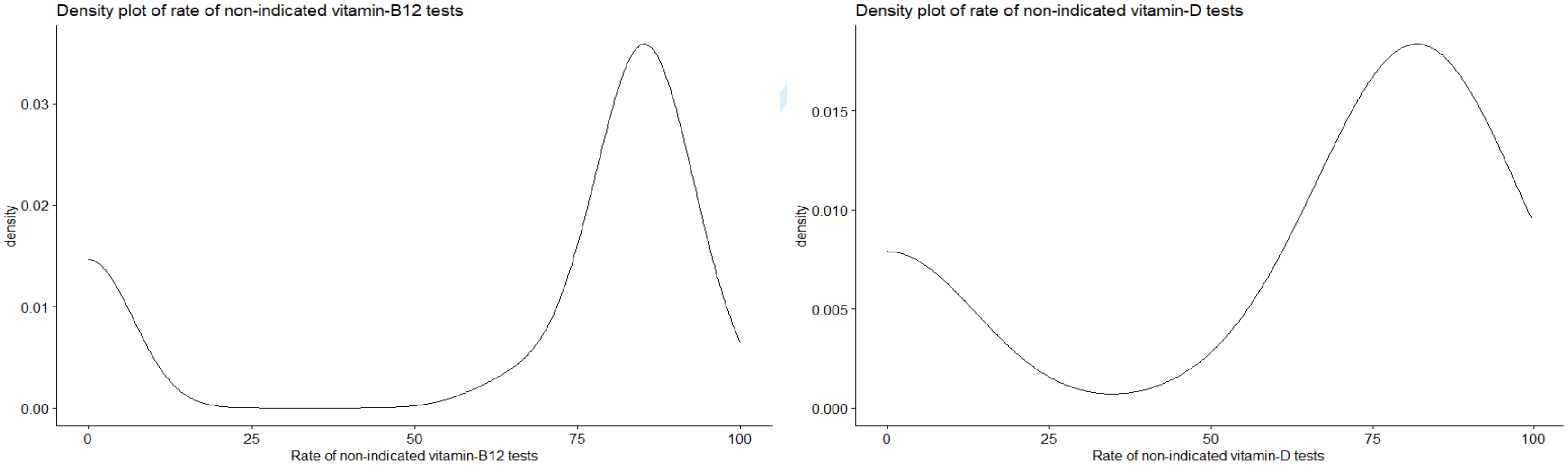

Figure 1: Density plots of rates of vitamin B12- and D-testing among the hospitals included

Table 2: Spearman's rank correlation Rho test outcomes

Rho	P-value
0.86	P < 2.2e-16

Correlation analysis outcome before removal of the outlier

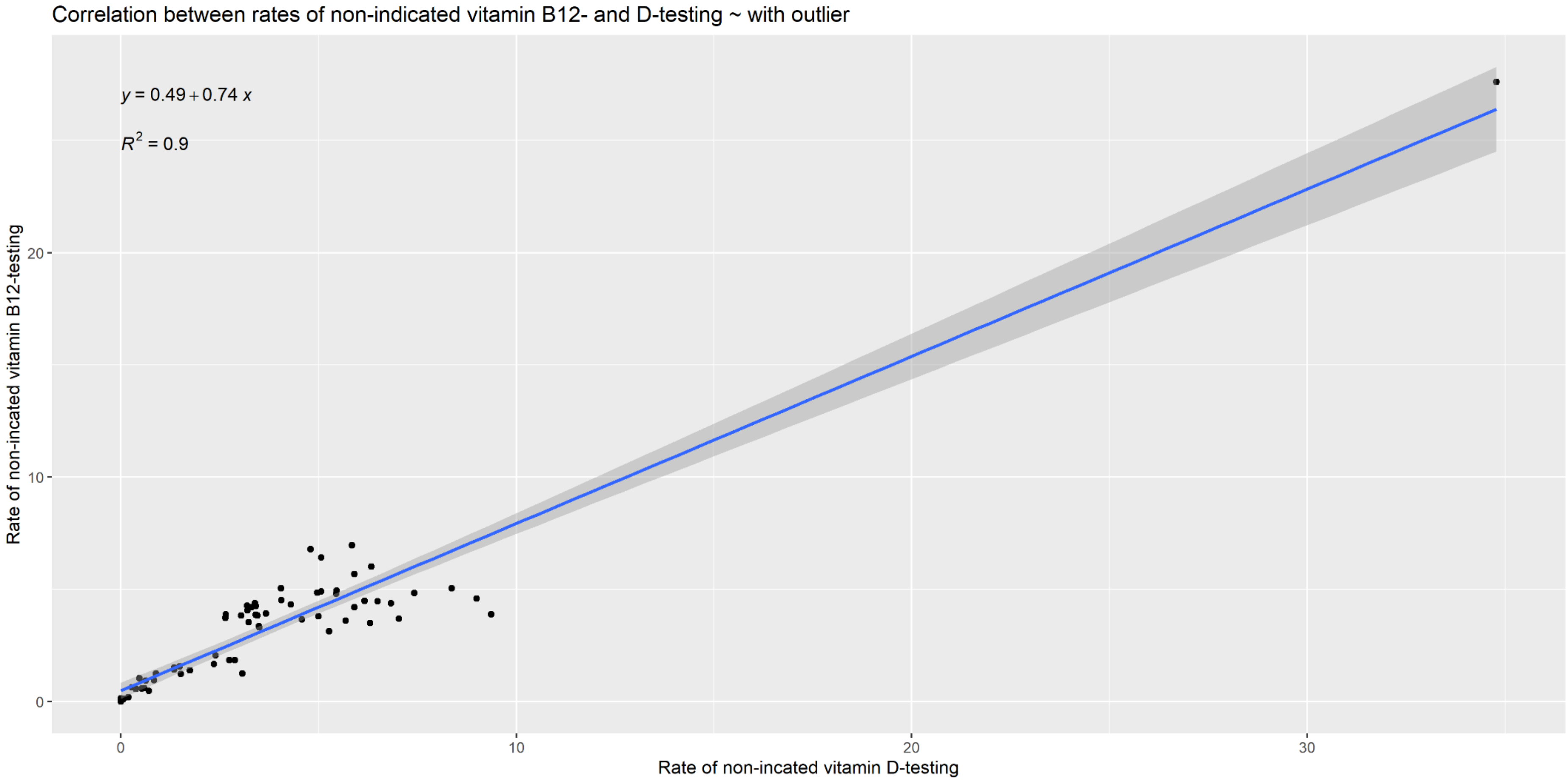

Figure 2: rates of non-indicated vitamin B12- and D-testing before removal of the outlier

STROBE Statement—Checklist of items that should be included in reports of ***cross-sectional studies***

	Item No	Recommendation	Page No
**Title and abstract**	1	(*a*) Indicate the study's design with a commonly used term in the title or the abstract	1
		(*b*) Provide in the abstract an informative and balanced summary of what was done and what was found	2
**Introduction**			
Background/rationale	2	Explain the scientific background and rationale for the investigation being reported	4 - 5
Objectives	3	State specific objectives, including any prespecified hypotheses	4 - 5
**Methods**			
Study design	4	Present key elements of study design early in the paper	5 - 8
Setting	5	Describe the setting, locations, and relevant dates, including periods of recruitment, exposure, follow-up, and data collection	5
Participants	6	(*a*) Give the eligibility criteria, and the sources and methods of selection of participants	5 - 8
Variables	7	Clearly define all outcomes, exposures, predictors, potential confounders, and effect modifiers. Give diagnostic criteria, if applicable	5 - 8
Data sources/ measurement	8*	For each variable of interest, give sources of data and details of methods of assessment (measurement). Describe comparability of assessment methods if there is more than one group	5 - 8
Bias	9	Describe any efforts to address potential sources of bias	N.A.
Study size	10	Explain how the study size was arrived at	N.A.
Quantitative variables	11	Explain how quantitative variables were handled in the analyses. If applicable, describe which groupings were chosen and why	5 - 8
Statistical methods	12	(*a*) Describe all statistical methods, including those used to control for confounding	5 - 8
		(*b*) Describe any methods used to examine subgroups and interactions	5 - 8
		(*c*) Explain how missing data were addressed	5 - 8
		(*d*) If applicable, describe analytical methods taking account of sampling strategy	5 - 8
		(*e*) Describe any sensitivity analyses	5 - 8
**Results**			
Participants	13*	(a) Report numbers of individuals at each stage of study—eg numbers potentially eligible, examined for eligibility, confirmed eligible, included in the study, completing follow-up, and analysed	9 - 11
		(b) Give reasons for non-participation at each stage	N.A.
		(c) Consider use of a flow diagram	N.A.
Descriptive data	14*	(a) Give characteristics of study participants (eg demographic, clinical, social) and information on exposures and potential confounders	9 - 11
		(b) Indicate number of participants with missing data for each variable of interest	9 - 11
Outcome data	15*	Report numbers of outcome events or summary measures	9 - 11
Main results	16	(*a*) Give unadjusted estimates and, if applicable, confounder-adjusted estimates and their precision (eg, 95% confidence interval). Make clear which confounders were adjusted for and why they were included	9 - 11

For peer review only - http://bmjopen.bmj.com/site/about/guidelines.xhtml

		(*b*) Report category boundaries when continuous variables were categorized	N.A.
		(*c*) If relevant, consider translating estimates of relative risk into absolute risk for a meaningful time period	N.A.
Other analyses	17	Report other analyses done—eg analyses of subgroups and interactions, and sensitivity analyses	9 – 11
**Discussion**			
Key results	18	Summarise key results with reference to study objectives	13 - 14
Limitations	19	Discuss limitations of the study, taking into account sources of potential bias or imprecision. Discuss both direction and magnitude of any potential bias	14 - 16
Interpretation	20	Give a cautious overall interpretation of results considering objectives, limitations, multiplicity of analyses, results from similar studies, and other relevant evidence	12 - 17
Generalisability	21	Discuss the generalisability (external validity) of the study results	15 - 17
**Other information**			
Funding	22	Give the source of funding and the role of the funders for the present study and, if applicable, for the original study on which the present article is based	17

*Give information separately for exposed and unexposed groups.

Note: An Explanation and Elaboration article discusses each checklist item and gives methodological background and published examples of transparent reporting. The STROBE checklist is best used in conjunction with this article (freely available on the Web sites of PLoS Medicine at http://www.plosmedicine.org/, Annals of Internal Medicine at http://www.annals.org/, and Epidemiology at http://www.epidem.com/). Information on the STROBE Initiative is available at www.strobe-statement.org.

For peer review only

For peer review only - http://bmjopen.bmj.com/site/about/guidelines.xhtml
